# Supplementary material for: Predictive value of abnormal blood tests for detecting cancer in primary care patients with nonspecific abdominal symptoms: A population-based cohort study of 477,870 patients in England
Source: PLoS Med. 2024 Jul 30;21(7):e1004426. doi: 10.1371/journal.pmed.1004426 (PMC11288431; doi:10.1371/journal.pmed.1004426)
Supplement: S1 Supplementary File — Table A. Read codes for abdominal pain and bloating. Table B. Evidence and rationale for blood tests included in the study. *readterms assigned to each code were manually reviewed by a clinician and irrelevant terms were removed. Table C. ICD-10 codes for Cancer. Fig A. Creation of the final tested symptomatic population by combining 2 subgroups of patients who had a blood test request around the time of abdominal symptom presentation identified by 2 different presentation scenarios. Fig B. Monthly cancer incidence following new GP presentation of abdominal pain or abdominal bloating. Statistically estimated inflection point is 10 months post symptom presentation. Table D. Risk ratios (observed/expected) for cancer in the 12 months following abdominal pain or bloating compared to expected rates in the population. Population rates use ONS cancer incidence and population size estimates for mid-2011; (excess risk = observed risk-expected risk). Table E. Predictive value for cancer diagnosis by sex at different time points after presenting to primary care with abdominal pain or bloating. Males, n = 156,590 for abdominal pain and n = 14,255 for abdominal bloating; females, n = 268,959 for abdominal pain and n = 38,066 for abdominal bloating. PPV, positive predictive value; m, month. Table F. Breakdown of cancer diagnosis by site and sex in patients presenting to primary care with abdominal pain or bloating who develop cancer in the following 12 months. Top section = males (n = 4,372 for abdominal pain and n = 367 for abdominal bloating). Bottom section = females (n = 5,055 for abdominal pain and n = 781 for abdominal bloating); P-value from chi squared test. Table G. Blood test use in the 3 months pre/post GP presentation with new onset abdominal pain or bloating in patients with ≥1 blood test, by sex. SD, standard deviation; IQR, interquartile range; *for 16 prespecified blood tests in the 3 months before the index date. Table H. Baseline characteristics of patients pre [file pmed.1004426.s001.docx]

## Supplementary Data

##### Table A: Read codes for abdominal pain and bloating

| Read Code | Term |
| --- | --- |
| 1969.00 | Abdominal pain |
| 1972.00 | Epigastric pain |
| 197..12 | Iliac fossa pain |
| R090500 | [D]Epigastric pain |
| R090700 | [D]Hypochondrial pain |
| 1975.00 | Left flank pain |
| R090400 | [D]Abdominal cramps |
| R090F00 | [D]Acute abdomen |
| 1977.00 | Right iliac fossa pain |
| 1829.00 | Retrosternal pain |
| R090200 | [D]Colic NOS |
| R090.00 | [D]Abdominal pain |
| 196..11 | Abdominal pain type |
| R090100 | [D]Abdominal colic |
| R090E00 | [D]Recurrent acute abdominal pain |
| 1968.00 | Abdominal discomfort |
| 1978.00 | Left iliac fossa pain |
| 1976.00 | Right flank pain |
| R090z00 | [D]Abdominal pain NOS |
| 197B.00 | Upper abdominal pain |
| 1971.00 | Central abdominal pain |
| R090600 | [D]Umbilical pain |
| 1963.00 | Non-colicky abdominal pain |
| 25C..15 | O/E - abdomen tender |
| 2I18.12 | O/E - tenderness |
| 1A53.12 | C/O - lumbar pain |
| 197..13 | Site of abdominal pain |
| 197..14 | Subcostal pain |
| 196..12 | Type of GIT pain - symptom |
| 1979.00 | Suprapubic pain |
| 197..11 | Flank pain |
| R090J00 | [D]Right upper quadrant pain |
| 1962.00 | Colicky abdominal pain |
| R090K00 | [D]Left upper quadrant pain |
| R090H00 | [D]Upper abdominal pain |
| R090L00 | [D]Left lower quadrant pain |
| 197D.00 | Right upper quadrant pain |
| 197A.11 | General abdominal pain-symptom |
| 25C8.00 | O/E - abd. pain - R.iliac |
| 25C2.00 | O/E - abd.pain-R.hypochondrium |
| 2I18100 | Tenderness of epigastrium |
| 25CZ.00 | O/E -abd.pain on palpation NOS |
| 196..00 | Type of GIT pain |
| 25C..00 | O/E - abdo. pain on palpation |
| 2I18.00 | O/E - tenderness/pain |
| R090C00 | [D]Loin pain |
| R090000 | [D]Abdominal tenderness |
| R090900 | [D]Pain in right iliac fossa |
| R090A00 | [D]Pain in left iliac fossa |
| 25C..12 | O/E - iliac pain on palpation |
| 1DC5.00 | Griping pain |
| 25E..00 | O/E - rebound tenderness |
| 25C..14 | O/E - umbilical pain on palp. |
| 25C..13 | O/E - lumbar pain on palpation |
| 25C3.00 | O/E - abd. pain - epigastrium |
| R090N00 | [D]Nonspecific abdominal pain |
| R090M00 | [D]Right lower quadrant pain |
| R090800 | [D]Suprapubic pain |
| 25C..11 | O/E - epigastric pain on palp. |
| 25CA.00 | O/E - abd. pain - L.iliac |
| 197C.00 | Lower abdominal pain |
| 1973.00 | Left subcostal pain |
| 25D..11 | O/E - guarding of abdomen |
| 25C6.00 | O/E - abd. pain - umbilical |
| 197A.00 | Generalised abdominal pain |
| 197..00 | Site of GIT pain |
| 25C7.00 | O/E - abd. pain - L.lumbar |
| R073200 | [D]Gas pain (abdominal) |
| 1969000 | Abdominal wall pain |
| 197Z.00 | Site of GIT pain NOS |
| R090y00 | [D]Other specified abdominal pain |
| 25D..00 | O/E - guarding on palpation |
| 25D8.00 | O/E - guarding - R.iliac |
| 25C5.00 | O/E - abd. pain - R.lumbar |
| 25C4.00 | O/E - abd.pain-L.hypochondrium |
| 25D2.00 | O/E - guarding-R.hypochondrium |
| 25C9.00 | O/E - abd. pain - hypogastrium |
| 25D3.00 | O/E - guarding - epigastrium |
| 196Z.00 | Type of GIT pain NOS |
| 25F..00 | O/E - abdominal rigidity |
| Ryu1000 | [X]Pain localized to other parts of lower abdomen |
| Ryu1100 | [X]Other and unspecified abdominal pain |
| E278000 | Psychogenic pain unspecified |
| R096.00 | [D]Acute abdomen |
| 25D9.00 | O/E - guarding - hypogastrium |
| 25DA.00 | O/E - guarding - L.iliac |
| 25EZ.00 | O/E - rebound tenderness NOS |
| 25D4.00 | O/E - guarding-L.hypochondrium |
| 25D6.00 | O/E - guarding - umbilical |
| 25DZ.00 | O/E -guarding on palpation NOS |
| R090P00 | [D]Functional abdominal pain syndrome |
| 1974.00 | Right subcostal pain |
| 25F2.00 | O/E - board like abd. rigidity |
| R090311 | [D]Evening colic |
| R094.00 | [D]Abdominal rigidity |
| 19A3.00 | Abdomen feels distended |
| R073400 | [D]Bloating |
| 19B..12 | Bloating symptom |
| 19A..00 | Abdominal distension symptom |
| R073300 | [D]Abdominal distension, gaseous |
| 19A2.00 | Abdomen feels bloated |
| 19AZ.00 | Abd. distension symptom NOS |
| R073.00 | [D]Flatulence, eructation and gas pain |
| R073z00 | [D]Flatulence, eructation and gas pain NOS |
| 19B3.00 | Excessive belching |
| 19B2.00 | Excessive flatulence |
| 19B4.00 | Excessive eructation |
| R073000 | [D]Flatulence |
| R073100 | [D]Eructation |

##### Table B: Evidence and rationale for blood tests included in the study

*readterms assigned to each code were manually reviewed by a clinician and irrelevant terms were removed.

| **Blood test category** | **Individual blood tests** | **Rationale for inclusion** | **Medcode** | **CPRD test entity number*** |
| --- | --- | --- | --- | --- |
| Tumour markers | Prostate specific antigen (PSA) and CA125 | Established tumour markers used in primary care (21, 23). | 5013 14054 17671 22598  108070 18455 108374  96961 98487 10363 7977  9228 14565 108230 | 436, 276 |
| Acute phase reactants | Platelets, erythrocyte sedimentation rate (ESR), C reactive protein (CRP), ferritin and total white blood cell count (WBC) | Can increase as part of a cancer-related inflammatory response (25). | 7 26927 26926 4415 4006 3320 46 25450 57305 27038  14924 27037 14068 14066 14067 19809 18384 8491  19761 4566 18405 18698 15  13817 13818 26948 26325  22293 4996 26947 26946  48015 1955 18516 | 189, 273, 280, 169, 207 |
| Markers of iron deficiency or anaemia | ferritin and haemoglobin | Can be features of many cancers as a result of tumour related blood loss, bone marrow invasion, nutritional deficiencies and anaemia of chronic disease (26) | 4 10404 33284 13755 35749 3942  26910 26909 26272 26913 41531  26908 26912 13 13596 23817 16387  13860 45241 18384 8491 19761 4566 18405 18698 | 169, 173 |
| Liver or bone profile tests | Bilirubin, AST, ALT, ALP and calcium | Can be markers of cancer metastasis, as well as of multiple myeloma or hepatobiliary cancers. | 59 26898 1183 13724 37205  13725 66 44363 101070  101501 13718 13719 40896  13720 18368 18502 13721  51280 13722 6417 10565  14346 35 13716 26020  13717 38197 13710 24  13712 35544 13711 18542  23107 18091 13729 19757  19758 101211 77 13728  26900 7578 98168 41072  57209 27096 106610 | 158, 156, 155, 153, 159, 160 |
| Albumin | Albumin | Synthesis can be decreased due to cancer-related malnutrition, liver dysfunction and inflammation (27, 28) | 23 26892 26893 13708 | 152 |
| Glycosylated haemoglobin (HbA1c) | Glycosylated haemoglobin (HbA1c) | raised levels have been associated with increased risk of cancer, particularly of pancreatic origin (29-32), possibly due to direct invasion of the pancreatic parenchyma. | 14050 42360 40463 46079 19807 9958 5717 14052 13597 29218 13604 14053  96968 14049 14051 27040  39205 | 275 |
| Renal function tests | Creatinine | Raised creatinine is associated with some cancers (33, 34). Postulated mechanisms include obstruction and reduced renal function in urological cancers (35), or malignancy related rises in creatinine due to increased cell or protein turnover. | 5 31277 35545 26903  3927 42345 62062  45096 13736 27095 | 165 |

##### Table C: ICD-10 codes for Cancer

| ICD10 Code | Term |
| --- | --- |
| C000 | Non-specific head and neck |
| C001 | Non-specific head and neck |
| C002 | Non-specific head and neck |
| C003 | Non-specific head and neck |
| C004 | Non-specific head and neck |
| C005 | Non-specific head and neck |
| C006 | Non-specific head and neck |
| C008 | Non-specific head and neck |
| C009 | Non-specific head and neck |
| C01 | Oropharynx |
| C020 | Oral cavity |
| C021 | Oral cavity |
| C022 | Oral cavity |
| C023 | Oral cavity |
| C024 | Oral cavity |
| C028 | Oral cavity |
| C029 | Oral cavity |
| C030 | Oral cavity |
| C031 | Oral cavity |
| C039 | Oral cavity |
| C040 | Oral cavity |
| C041 | Oral cavity |
| C048 | Oral cavity |
| C049 | Oral cavity |
| C050 | Other head and neck |
| C051 | Other head and neck |
| C052 | Other head and neck |
| C058 | Other head and neck |
| C059 | Other head and neck |
| C060 | Oral cavity |
| C061 | Oral cavity |
| C062 | Oral cavity |
| C068 | Oral cavity |
| C069 | Oral cavity |
| C07 | Other head and neck |
| C080 | Other head and neck |
| C081 | Other head and neck |
| C088 | Other head and neck |
| C089 | Other head and neck |
| C090 | Oropharynx |
| C091 | Oropharynx |
| C098 | Oropharynx |
| C099 | Oropharynx |
| C100 | Oropharynx |
| C101 | Oropharynx |
| C102 | Oropharynx |
| C103 | Oropharynx |
| C104 | Oropharynx |
| C108 | Oropharynx |
| C109 | Oropharynx |
| C110 | Other head and neck |
| C111 | Other head and neck |
| C112 | Other head and neck |
| C113 | Other head and neck |
| C118 | Other head and neck |
| C119 | Other head and neck |
| C12 | Other head and neck |
| C130 | Other head and neck |
| C131 | Other head and neck |
| C132 | Other head and neck |
| C138 | Other head and neck |
| C139 | Other head and neck |
| C140 | Non-specific head and neck |
| C142 | Non-specific head and neck |
| C148 | Non-specific head and neck |
| C150 | Oesophagus |
| C151 | Oesophagus |
| C152 | Oesophagus |
| C153 | Oesophagus |
| C154 | Oesophagus |
| C155 | Oesophagus |
| C158 | Oesophagus |
| C159 | Oesophagus |
| C160 | Stomach |
| C161 | Stomach |
| C162 | Stomach |
| C163 | Stomach |
| C164 | Stomach |
| C165 | Stomach |
| C166 | Stomach |
| C168 | Stomach |
| C169 | Stomach |
| C170 | Other malignant neoplasms |
| C171 | Other malignant neoplasms |
| C172 | Other malignant neoplasms |
| C173 | Other malignant neoplasms |
| C178 | Other malignant neoplasms |
| C179 | Other malignant neoplasms |
| C180 | Colon |
| C181 | Colon |
| C182 | Colon |
| C183 | Colon |
| C184 | Colon |
| C185 | Colon |
| C186 | Colon |
| C187 | Colon |
| C188 | Colon |
| C189 | Colon |
| C19 | Colon |
| C20 | Rectum |
| C210 | Other malignant neoplasms |
| C211 | Other malignant neoplasms |
| C212 | Other malignant neoplasms |
| C218 | Other malignant neoplasms |
| C220 | Liver |
| C221 | Liver |
| C222 | Liver |
| C223 | Liver |
| C224 | Liver |
| C227 | Liver |
| C229 | Liver |
| C23 | Other malignant neoplasms |
| C240 | Other malignant neoplasms |
| C241 | Other malignant neoplasms |
| C248 | Other malignant neoplasms |
| C249 | Other malignant neoplasms |
| C250 | Pancreas |
| C251 | Pancreas |
| C252 | Pancreas |
| C253 | Pancreas |
| C254 | Pancreas |
| C257 | Pancreas |
| C258 | Pancreas |
| C259 | Pancreas |
| C260 | Other malignant neoplasms |
| C261 | Other malignant neoplasms |
| C268 | Other malignant neoplasms |
| C269 | Other malignant neoplasms |
| C300 | Other malignant neoplasms |
| C301 | Other malignant neoplasms |
| C310 | Non-specific head and neck |
| C311 | Non-specific head and neck |
| C312 | Non-specific head and neck |
| C313 | Non-specific head and neck |
| C318 | Non-specific head and neck |
| C319 | Non-specific head and neck |
| C320 | Larynx |
| C321 | Larynx |
| C322 | Larynx |
| C323 | Larynx |
| C328 | Larynx |
| C329 | Larynx |
| C33 | Lung |
| C340 | Lung |
| C341 | Lung |
| C342 | Lung |
| C343 | Lung |
| C348 | Lung |
| C349 | Lung |
| C37 | Other malignant neoplasms |
| C380 | Other malignant neoplasms |
| C381 | Other malignant neoplasms |
| C382 | Other malignant neoplasms |
| C383 | Other malignant neoplasms |
| C384 | Other malignant neoplasms |
| C388 | Other malignant neoplasms |
| C390 | Other malignant neoplasms |
| C398 | Other malignant neoplasms |
| C399 | Other malignant neoplasms |
| C400 | Bone sarcoma |
| C401 | Bone sarcoma |
| C402 | Bone sarcoma |
| C403 | Bone sarcoma |
| C408 | Bone sarcoma |
| C409 | Bone sarcoma |
| C410 | Bone sarcoma |
| C411 | Bone sarcoma |
| C412 | Bone sarcoma |
| C413 | Bone sarcoma |
| C414 | Bone sarcoma |
| C418 | Bone sarcoma |
| C419 | Bone sarcoma |
| C430 | Melanoma |
| C431 | Melanoma |
| C432 | Melanoma |
| C433 | Melanoma |
| C434 | Melanoma |
| C435 | Melanoma |
| C436 | Melanoma |
| C437 | Melanoma |
| C438 | Melanoma |
| C439 | Melanoma |
| C450 | Mesothelioma |
| C451 | Mesothelioma |
| C452 | Mesothelioma |
| C457 | Mesothelioma |
| C459 | Mesothelioma |
| C460 | Other malignant neoplasms |
| C461 | Other malignant neoplasms |
| C462 | Other malignant neoplasms |
| C463 | Other malignant neoplasms |
| C467 | Other malignant neoplasms |
| C468 | Other malignant neoplasms |
| C469 | Other malignant neoplasms |
| C470 | Other malignant neoplasms |
| C471 | Other malignant neoplasms |
| C472 | Other malignant neoplasms |
| C473 | Other malignant neoplasms |
| C474 | Other malignant neoplasms |
| C475 | Other malignant neoplasms |
| C476 | Other malignant neoplasms |
| C478 | Other malignant neoplasms |
| C479 | Other malignant neoplasms |
| C480 | Connective and soft tissue sarcoma |
| C481 | Connective and soft tissue sarcoma |
| C482 | Connective and soft tissue sarcoma |
| C488 | Connective and soft tissue sarcoma |
| C490 | Connective and soft tissue sarcoma |
| C491 | Connective and soft tissue sarcoma |
| C492 | Connective and soft tissue sarcoma |
| C493 | Connective and soft tissue sarcoma |
| C494 | Connective and soft tissue sarcoma |
| C495 | Connective and soft tissue sarcoma |
| C496 | Connective and soft tissue sarcoma |
| C498 | Connective and soft tissue sarcoma |
| C499 | Connective and soft tissue sarcoma |
| C500 | Breast |
| C501 | Breast |
| C502 | Breast |
| C503 | Breast |
| C504 | Breast |
| C505 | Breast |
| C506 | Breast |
| C508 | Breast |
| C509 | Breast |
| C510 | Vulva |
| C511 | Vulva |
| C512 | Vulva |
| C518 | Vulva |
| C519 | Vulva |
| C52 | Other malignant neoplasms |
| C530 | Cervix |
| C531 | Cervix |
| C538 | Cervix |
| C539 | Cervix |
| C540 | Uterus |
| C541 | Uterus |
| C542 | Uterus |
| C543 | Uterus |
| C548 | Uterus |
| C549 | Uterus |
| C55 | Uterus |
| C56 | Ovary |
| C570 | Ovary |
| C571 | Ovary |
| C572 | Ovary |
| C573 | Ovary |
| C574 | Ovary |
| C577 | Ovary |
| C578 | Ovary |
| C579 | Ovary |
| C58 | Other malignant neoplasms |
| C600 | Other malignant neoplasms |
| C601 | Other malignant neoplasms |
| C602 | Other malignant neoplasms |
| C608 | Other malignant neoplasms |
| C609 | Other malignant neoplasms |
| C61 | Prostate |
| C620 | Testis |
| C621 | Testis |
| C629 | Testis |
| C630 | Other malignant neoplasms |
| C631 | Other malignant neoplasms |
| C632 | Other malignant neoplasms |
| C637 | Other malignant neoplasms |
| C638 | Other malignant neoplasms |
| C639 | Other malignant neoplasms |
| C64 | Kidney |
| C65 | Other and unspecified urinary |
| C66 | Other and unspecified urinary |
| C670 | Bladder |
| C671 | Bladder |
| C672 | Bladder |
| C673 | Bladder |
| C674 | Bladder |
| C675 | Bladder |
| C676 | Bladder |
| C677 | Bladder |
| C678 | Bladder |
| C679 | Bladder |
| C680 | Other and unspecified urinary |
| C681 | Other and unspecified urinary |
| C688 | Other and unspecified urinary |
| C689 | Other and unspecified urinary |
| C690 | Other malignant neoplasms |
| C691 | Other malignant neoplasms |
| C692 | Other malignant neoplasms |
| C693 | Other malignant neoplasms |
| C694 | Other malignant neoplasms |
| C695 | Other malignant neoplasms |
| C696 | Other malignant neoplasms |
| C698 | Other malignant neoplasms |
| C699 | Other malignant neoplasms |
| C700 | Meninges |
| C701 | Meninges |
| C709 | Meninges |
| C710 | Brain |
| C711 | Brain |
| C712 | Brain |
| C713 | Brain |
| C714 | Brain |
| C715 | Brain |
| C716 | Brain |
| C717 | Brain |
| C718 | Brain |
| C719 | Brain |
| C720 | Other CNS and intracranial |
| C721 | Other CNS and intracranial |
| C722 | Other CNS and intracranial |
| C723 | Other CNS and intracranial |
| C724 | Other CNS and intracranial |
| C725 | Other CNS and intracranial |
| C728 | Other malignant neoplasms |
| C729 | Other malignant neoplasms |
| C73 | Thyroid |
| C740 | Other malignant neoplasms |
| C741 | Other malignant neoplasms |
| C749 | Other malignant neoplasms |
| C750 | Other malignant neoplasms |
| C751 | Other CNS and intracranial |
| C752 | Other CNS and intracranial |
| C753 | Other CNS and intracranial |
| C754 | Other malignant neoplasms |
| C755 | Other malignant neoplasms |
| C758 | Other malignant neoplasms |
| C759 | Other malignant neoplasms |
| C760 | Other malignant neoplasms |
| C761 | Other malignant neoplasms |
| C762 | Other malignant neoplasms |
| C763 | Other malignant neoplasms |
| C764 | Other malignant neoplasms |
| C765 | Other malignant neoplasms |
| C767 | Other malignant neoplasms |
| C768 | Other malignant neoplasms |
| C770 | Unknown primary |
| C771 | Unknown primary |
| C772 | Unknown primary |
| C773 | Unknown primary |
| C774 | Unknown primary |
| C775 | Unknown primary |
| C778 | Unknown primary |
| C779 | Unknown primary |
| C780 | Unknown primary |
| C781 | Unknown primary |
| C782 | Unknown primary |
| C783 | Unknown primary |
| C784 | Unknown primary |
| C785 | Unknown primary |
| C786 | Unknown primary |
| C787 | Unknown primary |
| C788 | Unknown primary |
| C790 | Unknown primary |
| C791 | Unknown primary |
| C792 | Unknown primary |
| C793 | Unknown primary |
| C794 | Unknown primary |
| C795 | Unknown primary |
| C796 | Unknown primary |
| C797 | Unknown primary |
| C798 | Unknown primary |
| C80 | Unknown primary |
| C800 | Unknown primary |
| C809 | Unknown primary |
| C810 | Hodgkin lymphoma |
| C811 | Hodgkin lymphoma |
| C812 | Hodgkin lymphoma |
| C813 | Hodgkin lymphoma |
| C817 | Hodgkin lymphoma |
| C819 | Hodgkin lymphoma |
| C820 | Non-hodgkin lymphoma |
| C821 | Non-hodgkin lymphoma |
| C822 | Non-hodgkin lymphoma |
| C827 | Non-hodgkin lymphoma |
| C829 | Non-hodgkin lymphoma |
| C830 | Non-hodgkin lymphoma |
| C831 | Non-hodgkin lymphoma |
| C832 | Non-hodgkin lymphoma |
| C833 | Non-hodgkin lymphoma |
| C834 | Non-hodgkin lymphoma |
| C835 | Non-hodgkin lymphoma |
| C836 | Non-hodgkin lymphoma |
| C837 | Non-hodgkin lymphoma |
| C838 | Non-hodgkin lymphoma |
| C839 | Non-hodgkin lymphoma |
| C840 | Non-hodgkin lymphoma |
| C841 | Non-hodgkin lymphoma |
| C842 | Non-hodgkin lymphoma |
| C843 | Non-hodgkin lymphoma |
| C844 | Non-hodgkin lymphoma |
| C845 | Non-hodgkin lymphoma |
| C850 | Non-hodgkin lymphoma |
| C851 | Non-hodgkin lymphoma |
| C857 | Non-hodgkin lymphoma |
| C859 | Non-hodgkin lymphoma |
| C880 | Other haematological |
| C881 | Other haematological |
| C882 | Other haematological |
| C883 | Other haematological |
| C887 | Other haematological |
| C889 | Other haematological |
| C900 | Multiple myeloma |
| C901 | Multiple myeloma |
| C902 | Multiple myeloma |
| C910 | Other leukaemia |
| C911 | Chronic lymphocytic leukaemia |
| C912 | Other haematological |
| C913 | Other haematological |
| C914 | Other haematological |
| C915 | Other haematological |
| C917 | Other haematological |
| C919 | Other haematological |
| C920 | Acute myeloid leukaemia |
| C921 | Other leukaemia |
| C922 | Other haematological |
| C923 | Other haematological |
| C924 | Acute myeloid leukaemia |
| C925 | Acute myeloid leukaemia |
| C927 | Other haematological |
| C929 | Other haematological |
| C930 | Acute myeloid leukaemia |
| C931 | Other haematological |
| C932 | Other haematological |
| C937 | Other haematological |
| C939 | Other haematological |
| C940 | Acute myeloid leukaemia |
| C942 | Acute myeloid leukaemia |
| C943 | Other haematological |
| C944 | Other haematological |
| C945 | Other haematological |
| C947 | Other haematological |
| C950 | Other haematological |
| C951 | Other haematological |
| C952 | Other haematological |
| C957 | Other haematological |
| C959 | Other haematological |
| C960 | Other haematological |
| C961 | Other haematological |
| C962 | Other haematological |
| C963 | Other haematological |
| C967 | Other haematological |
| C969 | Other haematological |
| C97 | Other malignant neoplasms |
| D050 | Breast (in-situ) |
| D051 | Breast (in-situ) |
| D057 | Breast (in-situ) |
| D059 | Breast (in-situ) |
| D060 | Cervix (in-situ) |
| D061 | Cervix (in-situ) |
| D067 | Cervix (in-situ) |
| D069 | Cervix (in-situ) |
| D090 | Bladder (in-situ) |
| D320 | Meninges |
| D321 | Meninges |
| D329 | Meninges |
| D330 | Brain |
| D331 | Brain |
| D332 | Brain |
| D333 | Other CNS and intracranial |
| D334 | Other CNS and intracranial |
| D352 | Other CNS and intracranial |
| D353 | Other CNS and intracranial |
| D354 | Other CNS and intracranial |
| D420 | Meninges |
| D421 | Meninges |
| D429 | Meninges |
| D430 | Brain |
| D431 | Brain |
| D432 | Brain |
| D433 | Other CNS and intracranial |
| D434 | Other CNS and intracranial |
| D443 | Other CNS and intracranial |
| D444 | Other CNS and intracranial |
| D445 | Other CNS and intracranial |

##### Figure A: Creation of the final tested symptomatic population by combining two subgroups of patients who had a blood test request around the time of abdominal symptom presentation identified by two different presentation scenarios.

##### Figure B: Monthly cancer incidence following new GP presentation of abdominal pain or abdominal bloating. Statistically estimated inflection point is 10 months post symptom presentation.

**INCIDENCE OF CANCER FOLLOWING ABDOMINAL PAIN:**


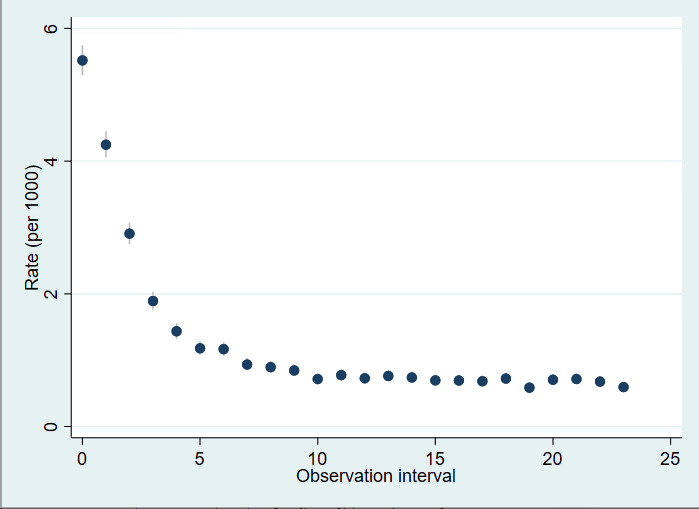


**INCIDENCE OF CANCER FOLLOWING ABDOMINAL BLOATING:**


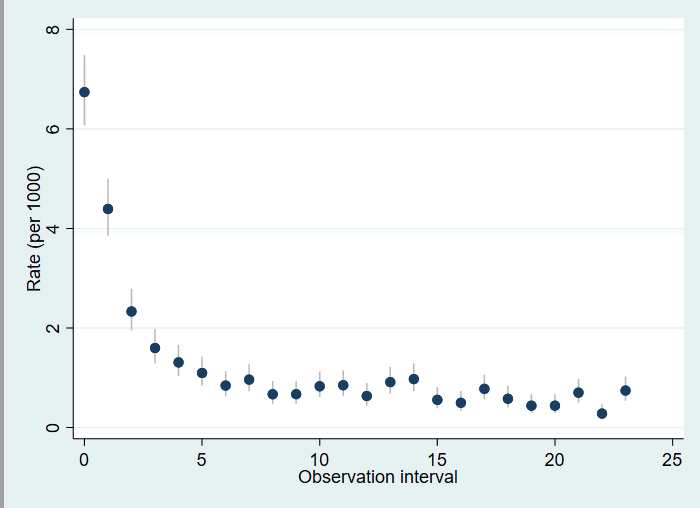


##### Table D: Risk Ratios (observed/expected) for cancer in the 12 months following abdominal pain or bloating compared to expected rates in the population

Population rates use ONS cancer incidence and population size estimates for mid-2011; (excess risk = observed risk-expected risk).

| **Age group (years)** | **Risk Ratios for cancer in the next 12 months** | | **Excess Risk of cancer compared to general population** | |
| --- | --- | --- | --- | --- |
|  | **Abdominal Pain** | **Abdominal Bloating** | **Abdominal Pain** | **Abdominal Bloating** |
| **Men** | N=156,590 | N=14,255 | N=156,590 | N=14,255 |
| 30-39 | 4.03 (3.22 – 5.05) | 4.73 (2.13 – 10.53) | 0.20% (0.14 – 0.26) | 0.25% (0.00 – 0.51) |
| 40-49 | 3.78 (3.30 – 4.33) | 3.18 (1.92 – 5.26) | 0.43% (0.35 – 0.51) | 0.34% (0.09 – 0.59) |
| 50-59 | 3.77 (3.49 – 4.08) | 3.23 (2.46 – 4.23) | 1.42% (1.27 – 1.56) | 1.14% (0.69 – 1.58) |
| 60-69 | 3.05 (2.88 – 3.22) | 2.19 (1.80 – 2.67) | 2.84% (2.61 – 3.07) | 1.65% (1.05 – 2.26) |
| 70-79 | 2.72 (2.58 – 2.86) | 2.51 (2.13 – 2.96) | 4.34% (3.99 – 4.70) | 3.81% (2.76 – 4.86) |
| ≥80 | 2.63 (2.47 – 2.81) | 2.07 (1.65 – 2.60) | 5.30% (4.75 – 5.85) | 3.48% (1.95 – 5.02) |
| **Women** | N=268,959 | N=38,066 | N=268,959 | N=38,066 |
| 30-39 | 3.43 (3.04 – 3.86) | 3.62 (2.54 – 5.15) | 0.30% (0.25 – 0.35) | 0.32% (0.17 – 0.48) |
| 40-49 | 2.42 (2.21 – 2.65) | 2.33 (1.86 – 2.93) | 0.45% (0.38 – 0.52) | 0.42% (0.26 – 0.59) |
| 50-59 | 2.37 (2.20 – 2.55) | 2.53 (2.12 – 3.01) | 0.86% (0.75 – 0.97) | 0.96% (0.68 – 1.23) |
| 60-69 | 2.82 (2.67 – 2.98) | 3.19 (2.79 – 3.64) | 2.01% (1.84 – 2.17) | 2.41% (1.95 – 2.87) |
| 70-79 | 2.75 (2.60 – 2.91) | 3.00 (2.62 – 3.43) | 2.77% (2.54 – 3.01) | 3.16% (2.52 – 3.80) |
| ≥80 | 2.51 (2.36 – 2.67) | 2.27 (1.92 – 2.68) | 3.09% (2.78 – 3.41) | 2.60% (1.83 – 3.37) |

##### Table E: Predictive value for cancer diagnosis by sex at different time points after presenting to primary care with abdominal pain or bloating

Males n=156590 for abdominal pain and n=14255 for abdominal bloating; Females n=268959 for abdominal pain and n=38066 for abdominal bloating. PPV, positive predictive value; m, month.

|  | **Males with abdominal pain** | | | **Males with abdominal bloating** | | |
| --- | --- | --- | --- | --- | --- | --- |
| **Cancer site** | 6m PPV | 12m PPV | 24m PPV | 6m PPV | 12m PPV | 24m PPV |
| **Any Cancer** | **2.2** | **2.8** | **3.7** | **2.0** | **2.6** | **3.6** |
| Colon | 0.5 | 0.6 | 0.7 | 0.5 | 0.5 | 0.7 |
| Prostate | 0.3 | 0.4 | 0.6 | 0.2 | 0.3 | 0.6 |
| Lung | 0.2 | 0.2 | 0.3 | 0.1 | 0.2 | 0.3 |
| Pancreas | 0.2 | 0.3 | 0.3 | 0.2 | 0.3 | 0.3 |
| Stomach | 0.1 | 0.2 | 0.2 | 0.1 | 0.2 | 0.2 |
| Lymphoma | 0.1 | 0.1 | 0.2 | 0.1 | 0.2 | 0.2 |
| Oesophagus | 0.1 | 0.1 | 0.2 | 0.1 | 0.1 | 0.2 |
| Unknown Primary | 0.1 | 0.1 | 0.2 | 0.1 | 0.1 | 0.1 |
| Kidney and Urinary Tract | 0.1 | 0.1 | 0.2 | 0.1 | 0.1 | 0.1 |
| Bladder | 0.1 | 0.1 | 0.2 | 0.1 | 0.1 | 0.1 |
| Rectum | 0.1 | 0.1 | 0.1 | 0.1 | 0.1 | 0.1 |
| Liver | 0.1 | 0.1 | 0.1 | 0.2 | 0.2 | 0.2 |
| Leukaemia | 0.0 | 0.0 | 0.1 | 0.0 | 0.0 | 0.1 |
| Head and Neck | 0.0 | 0.0 | 0.1 | 0.0 | 0.0 | 0.0 |
| Multiple Myeloma | 0.0 | 0.0 | 0.1 | 0.0 | 0.1 | 0.1 |
| Melanoma | 0.0 | 0.0 | 0.1 | 0.0 | 0.0 | 0.0 |
| Other | 0.1 | 0.2 | 0.3 | 0.2 | 0.2 | 0.3 |
| **Total Number Cancers** | **3,407** | **4,372** | **5,745** | **287** | **367** | **516** |
|  | **Females with abdominal pain** | | | **Females with abdominal bloating** | | |
| **Cancer site** | 6m PPV | 12m PPV | 24m PPV | 6m PPV | 12m PPV | 24m PPV |
| **Any Cancer** | **1.4** | **1.9** | **2.6** | **1.6** | **2.1** | **2.6** |
| Colon | 0.3 | 0.4 | 0.4 | 0.2 | 0.2 | 0.2 |
| Breast | 0.1 | 0.2 | 0.4 | 0.2 | 0.3 | 0.5 |
| Ovary | 0.2 | 0.2 | 0.3 | 0.6 | 0.6 | 0.6 |
| Pancreas | 0.1 | 0.2 | 0.2 | 0.1 | 0.1 | 0.1 |
| Lung | 0.1 | 0.1 | 0.2 | 0.1 | 0.1 | 0.1 |
| Cervix | 0.1 | 0.1 | 0.2 | 0.1 | 0.1 | 0.1 |
| Unknown Primary | 0.1 | 0.1 | 0.1 | 0.1 | 0.1 | 0.2 |
| Lymphoma | 0.1 | 0.1 | 0.1 | 0.0 | 0.0 | 0.1 |
| Uterus | 0.1 | 0.1 | 0.1 | 0.0 | 0.1 | 0.1 |
| Stomach | 0.0 | 0.1 | 0.1 | 0.0 | 0.0 | 0.1 |
| Kidney and Urinary Tract | 0.0 | 0.1 | 0.1 | 0.0 | 0.0 | 0.1 |
| Melanoma | 0.0 | 0.0 | 0.1 | 0.0 | 0.0 | 0.1 |
| Oesophagus | 0.0 | 0.0 | 0.1 | 0.0 | 0.0 | 0.0 |
| Rectum | 0.0 | 0.0 | 0.1 | 0.0 | 0.0 | 0.1 |
| Liver | 0.0 | 0.0 | 0.0 | 0.0 | 0.0 | 0.0 |
| Sarcoma | 0.0 | 0.0 | 0.0 | 0.1 | 0.1 | 0.1 |
| Other | 0.1 | 0.2 | 0.3 | 0.1 | 0.2 | 0.2 |
| **Total Number Cancers** | **3816** | **5055** | **7074** | **616** | **781** | **1010** |

##### Table F: Breakdown of cancer diagnosis by site and sex in patients presenting to primary care with abdominal pain or bloating who develop cancer in the following 12 months.

Top section = males (n=4372 for abdominal pain and n=367 for abdominal bloating). Bottom section = females (n=5055 for abdominal pain and n=781 for abdominal bloating); P value from chi squared test.

| **Male patients** | | | | | |
| --- | --- | --- | --- | --- | --- |
|  | **Abdominal pain** | | **Abdominal bloating** | |  |
| **Cancer site** | Number | % | Number | % | P |
| Colon | 951 | 21.8 | 76 | 20.7 | 0.64 |
| Prostate | 650 | 14.9 | 45 | 12.3 | 0.18 |
| Pancreas | 405 | 9.3 | 35 | 9.5 | 0.86 |
| Lung | 348 | 8.0 | 21 | 5.7 | 0.12 |
| Stomach | 264 | 6.0 | 22 | 6.0 | 0.97 |
| Lymphoma | 221 | 5.1 | 22 | 6.0 | 0.43 |
| Oesophagus | 206 | 4.7 | 18 | 4.9 | 0.87 |
| Unknown Primary | 200 | 4.6 | 17 | 4.6 | 0.96 |
| Kidney and Urinary Tract | 179 | 4.1 | 12 | 3.3 | 0.44 |
| Bladder | 152 | 3.5 | 11 | 3.0 | 0.74 |
| Other Malignant Neoplasm | 148 | 3.4 | 13 | 3.5 | 0.87 |
| Liver | 142 | 3.3 | 23 | 6.3 | **0.002** |
| Rectum | 135 | 3.1 | 12 | 3.3 | 0.85 |
| Leukaemia | 68 | 1.6 | 4 | 1.1 | 0.48 |
| Head and Neck | 50 | 1.1 | 3 | 0.8 | 0.10 |
| Multiple Myeloma | 48 | 1.1 | 8 | 2.2 | 0.07 |
| Melanoma | 46 | 1.1 | 5 | 1.4 | 0.58 |
| Mesothelioma | 44 | 1.0 | 6 | 1.6 | 0.26 |
| Brain and CNS | 38 | 0.9 | 5 | 1.4 | 0.34 |
| Sarcoma | 33 | 0.8 | 5 | 1.4 | 0.21 |
| Testis | 19 | 0.4 | 2 | 0.5 | 0.76 |
| Other Haematological Malignancy | 15 | 0.3 | 1 | 0.3 | 0.82 |
| Thyroid | 7 | 0.2 | 0 | 0.0 | - |
| Breast | 2 | 0.1 | 1 | 0.3 | 0.10 |
| Cervix | 1 | 0.0 | 0 | 0.0 | - |
| **Total** | **4,372** | **100.0** | **367** | **100.0** |  |
| **Female patients** | | | | | |
|  | **Abdominal pain** | | **Abdominal bloating** | |  |
| **Cancer site** | Number | % | Number | % | P |
| Colon | 982 | 19.4 | 80 | 10.2 | **<0.001** |
| Breast | 578 | 11.4 | 97 | 12.4 | 0.42 |
| Ovary | 576 | 11.4 | 233 | 29.8 | **<0.001** |
| Pancreas | 437 | 8.6 | 44 | 5.6 | **0.004** |
| Lung | 333 | 6.6 | 35 | 4.5 | **0.02** |
| Unknown Primary | 289 | 5.7 | 55 | 7.0 | 0.14 |
| Cervix | 264 | 5.2 | 40 | 5.1 | 0.91 |
| Lymphoma | 210 | 4.2 | 16 | 2.1 | **0.005** |
| Other Malignant Neoplasm | 186 | 3.7 | 19 | 2.4 | 0.08 |
| Uterus | 164 | 3.2 | 24 | 3.1 | 0.80 |
| Stomach | 145 | 2.9 | 17 | 2.2 | 0.27 |
| Kidney and Urinary Tract | 132 | 2.6 | 12 | 1.5 | 0.07 |
| Oesophagus | 106 | 2.1 | 7 | 0.9 | **0.02** |
| Rectum | 98 | 1.9 | 17 | 2.2 | 0.66 |
| Liver | 96 | 1.9 | 14 | 1.8 | 0.84 |
| Melanoma | 94 | 1.9 | 11 | 1.4 | 0.38 |
| Sarcoma | 78 | 1.5 | 23 | 2.9 | **0.005** |
| Bladder | 67 | 1.3 | 10 | 1.3 | 0.92 |
| Brain and CNS | 49 | 1.0 | 5 | 0.6 | 0.37 |
| Multiple Myeloma | 45 | 0.9 | 6 | 0.8 | 0.73 |
| Leukaemia | 36 | 0.7 | 6 | 0.8 | 0.86 |
| Thyroid | 24 | 0.5 | 2 | 0.3 | 0.39 |
| Head and Neck | 22 | 0.4 | 1 | 0.1 | 0.20 |
| Mesothelioma | 21 | 0.4 | 4 | 0.5 | 0.70 |
| Other Haematological Malignancy | 13 | 0.3 | 2 | 0.3 | 1.00 |
| Vulva | 10 | 0.2 | 1 | 0.1 | 0.68 |
| **Total** | **5,055** | **100.0** | **781** | **100.0** |  |

##### Table G: Blood test use in the 3 months pre / post GP presentation with new onset abdominal pain or bloating in patients with ≥1 blood test, by sex

SD, standard deviation; IQR, Interquartile range; *for 16 pre-specified blood tests in the 3 months before the index date

|  | **Abdominal pain patients** | | **Abdominal bloating patients** | |
| --- | --- | --- | --- | --- |
|  | **Tested males**  **N= 98,687** | **Tested females**  **N= 174,468** | **Tested males**  **N= 9,667** | **Tested females**  **N= 26,966** |
| Number of blood tests* |  |  |  |  |
| 1-4 | 17,706 (18%) | 38,956 (22%) | 1,645 (17%) | 5,204 (19%) |
| 5-8 | 26,078 (26%) | 43,090 (25%) | 2,539 (26%) | 5,951 (22%) |
| 9-12 | 47,359 (48%) | 79,141 (45%) | 4,635 (48%) | 13,241 (49%) |
| ≥13 | 7,544 (8%) | 13,281 (8%) | 848 (9%) | 2,570 (10%) |
| Mean (SD, range) | 8.4 (4.4, 1-112) | 8.3 (4.5, 1-114) | 8.6 (4.4, 1-60) | 8.6 (4.4, 1-70) |
| Median (IQR) | 9 (6-11) | 9 (5-10) | 9 (6-11) | 9 (5-11) |

##### Table H: Baseline characteristics of patients presenting to their GP with new onset abdominal pain with a blood test in the 3 months pre versus post presentation

Total n=273,155; patients can belong to both groups; P value compares both cohorts using chi squared test, SD, standard deviation; IQR, Interquartile range; *for 16 pre-specified blood tests

|  | **Blood test before symptom**  **N= 169,744** | **Blood test after symptom**  **N=209,860** | **P value** |
| --- | --- | --- | --- |
| Male sex | 59,351 (35%) | 75,389 (36%) | <0.001 |
| Age at presentation (years) |  |  |  |
| 30–39 | 26,642 (16%) | 35,135 (17%) | <0.001 |
| 40–49 | 33,343 (20%) | 44,749 (21%) |  |
| 50–59 | 33,106 (20%) | 42,591 (20%) |  |
| 60–69 | 33,906 (20%) | 40,631 (19%) |  |
| 70–79 | 26,725 (16%) | 29,710 (14%) |  |
| 80 and over | 16,022 (9%) | 17,044 (8%) |  |
| Mean (SD, range) | 57 (15.8, 30-104) | 56 (15.5, 30-103) |  |
| Median (IQR) | 57 (44-70) | 55 (44-68) |  |
| Year of Presentation |  |  | 0.001 |
| 2007–2008 | 41,352 (24%) | 50.166 (24%) |  |
| 2009–2010 | 41,529 (24%) | 51,009 (24%) |  |
| 2011–2012 | 38,023 (22%) | 47,163 (22%) |  |
| 2013–2014 | 30,974 (18%) | 38,911 (19%) |  |
| 2015–2016 | 17,866 (11%) | 22,611 (11%) |  |
| IMD quintile |  |  | <0.001 |
| 1 (least deprived) | 38,486 (23%) | 49,563 (24%) |  |
| 2 | 36,794 (22%) | 46,327 (22%) |  |
| 3 | 36,024 (21%) | 44,446 (21%) |  |
| 4 | 30,825 (18%) | 37,128 (18%) |  |
| 5 (most deprived) | 27,517 (16%) | 32,296 (15%) |  |
| Missing | 98 (0.1%) | 100 (0.1%) |  |
| Cancer within 12m of symptom | 5,071 (3.0%) | 5,428 (2.6%) | <0.001 |
| Number of blood tests* in 3m window |  |  | <0.001 |
| 1-4 | 37,075 (22%) | 34,814 (17%) |  |
| 5-8 | 42,088 (25%) | 46,664 (22%) |  |
| 9-12 | 70,784 (42%) | 97,301 (46%) |  |
| ≥13 | 19,797 (12%) | 31,081 (15%) |  |
| Mean (SD, range) | 8.6 (5.3, 1-114) | 9.4 (5.3, 1-117) |  |
| Median (IQR) | 9 (5-11) | 9 (7-11) |  |

##### Table I: Baseline characteristics of patients presenting to their GP with new onset abdominal bloating with a blood test in the 3 months pre versus post presentation

Total n=36,633; patients can belong to both groups; P value compares both cohorts using chi squared test, SD, standard deviation; IQR, Interquartile range; *for 16 pre-specified blood tests

|  | **Blood test before symptom**  **N= 19,553** | **Blood test after symptom**  **N=27,598** | **P value** |
| --- | --- | --- | --- |
| Male sex | 5,308 (27%) | 7,103 (26%) | 0.001 |
| Age at presentation (years) |  |  |  |
| 30–39 | 2,158 (11%) | 3,816 (14%) | <0.001 |
| 40–49 | 4,152 (21%) | 6,624 (24%) |  |
| 50–59 | 4,115 (21%) | 6,010 (22%) |  |
| 60–69 | 4,010 (21%) | 5,209 (19%) |  |
| 70–79 | 3,195 (16%) | 3,737 (14%) |  |
| 80 and over | 1,923 (10%) | 2,202 (8%) |  |
| Mean (SD, range) | 58 (15.1, 30-101) | 56 (14.9, 30-101) |  |
| Median (IQR) | 58 (46-70) | 55 (45-68) |  |
| Year of Presentation |  |  | <0.001 |
| 2007–2008 | 3,546 (18%) | 4,585 (17%) |  |
| 2009–2010 | 4,435 (23%) | 6,103 (22%) |  |
| 2011–2012 | 4,909 (25%) | 6,943 (25%) |  |
| 2013–2014 | 4,234 (22%) | 6,275 (23%) |  |
| 2015–2016 | 2,429 (12%) | 3,692 (13%) |  |
| IMD quintile |  |  | <0.001 |
| 1 (least deprived) | 4,451 (23%) | 6,882 (25%) |  |
| 2 | 4,294 (22%) | 6,156 (22%) |  |
| 3 | 4,035 (21%) | 5,723 (21%) |  |
| 4 | 3,509 (18%) | 4,753 (17%) |  |
| 5 (most deprived) | 3,254 (17%) | 4,071 (15%) |  |
| Missing | 10 (0.1%) | 13 (0.1%) |  |
| Cancer within 12m of symptom | 558 (2.9%) | 657 (2.4%) | 0.001 |
| Number of blood tests* in 3m window |  |  | <0.001 |
| 1-4 | 4,030 (21%) | 4,121 (15%) |  |
| 5-8 | 4,623 (24%) | 5,561 (20%) |  |
| 9-12 | 8,314 (43%) | 13,455 (49%) |  |
| ≥13 | 2,586 (13%) | 4,461 (16%) |  |
| Mean (SD, range) | 8.8 (5.2, 1-70) | 9.6 (5.0, 1-71) |  |
| Median (IQR) | 9 (5-11) | 10 (7-11) |  |

##### Table J: Positive predictive values (PPVs) for cancer in 12 months by sex in patients with abdominal pain and a blood test abnormality

Red = PPV of ≥10%; Orange = PPV of ≥3% (NICE threshold for recommending cancer investigation); Yellow = PPV ≥2%. n/N = number of patients with an abnormal test/all patients tested; LR, likelihood ratio; TM, tumour markers (PSA or CA125)

| **Patients presenting with abdominal pain** | | | | | | | | | | | | | |
| --- | --- | --- | --- | --- | --- | --- | --- | --- | --- | --- | --- | --- | --- |
|  | n/N | Sensitivity | | Specificity | | PPV abnormal result | | NPV normal result | | Positive LR | | Negative LR | |
| MALES |  |  |  |  |  |  |  |  |  |  |  |  |  |
| **Any blood test abnormality** | 56,897/98,687 | 77.88 | (76.47 - 79.24) | 43.10 | (42.78 - 43.41) | 4.83 | (4.66 - 5.01) | 98.13 | (98.00 - 98.26) | 1.37 | (1.34 – 1.39) | 0.51 | (0.48 – 0.55) |
| **Any abnormality except TM** | 55,076/97,179 | 74.36 | (72.86 – 75.81) | 43.98 | (43.66 – 44.29) | 4.66 | (4.48 – 4.84) | 97.90 | (97.76 – 98.03) | 1.33 | (1.30 – 1.35) | 0.58 | (0.55 – 0.62) |
| Low albumin | 2,981/77,115 | 14.03 | (12.75 - 15.39) | 96.51 | (96.37 - 96.64) | 12.92 | (11.73 - 14.17) | 96.82 | (96.69 - 96.94) | 4.02 | (3.64 - 4.44) | 0.89 | (0.88 -0.90) |
| Raised PSA | 3,329/13,448 | 53.53 | (49.92 - 57.12) | 76.98 | (76.24 - 77.71) | 12.29 | (11.19 - 13.45) | 96.49 | (96.11 - 96.84) | 2.33 | (2.16 - 2.50) | 0.60 | (0.56 -0.65) |
| Raised platelets | 2,656/77,207 | 11.39 | (10.24 - 12.62) | 96.86 | (96.73 - 96.99) | 12.09 | (10.87 - 13.39) | 96.72 | (96.59 - 96.85) | 3.63 | (3.25 - 4.05) | 0.89 | (0.88 -0.91) |
| Anaemia | 8,512/77,807 | 33.15 | (31.42 - 34.91) | 89.90 | (89.68 - 90.12) | 11.08 | (10.42 - 11.76) | 97.26 | (97.13 - 97.38) | 3.28 | (3.10 - 3.47) | 0.74 | (0.72 -0.76) |
| Raised calcium | 372/29,113 | 3.48 | (2.50 - 4.71) | 98.81 | (98.68 - 98.94) | 10.75 | (7.79 - 14.35) | 96.14 | (95.91 - 96.36) | 2.93 | (2.12 - 4.05) | 0.98 | (0.97 -0.99) |
| Raised ALP | 6,859/77,265 | 25.07 | (23.46 - 26.73) | 91.72 | (91.52 - 91.92) | 10.09 | (9.39 - 10.83) | 97.06 | (96.94 - 97.19) | 3.03 | (2.83 - 3.24) | 0.82 | (0.80 -0.83) |
| Low ferritin | 944/9,451 | 20.83 | (17.10 - 24.97) | 90.53 | (89.91 - 91.13) | 9.53 | (7.74 - 11.59) | 96.30 | (95.84 - 96.72) | 2.20 | (1.81 - 2.67) | 0.80 | (0.74 -0.86) |
| Raised ESR | 8,545/27,602 | 64.85 | (61.93 - 67.70) | 70.43 | (69.87 - 70.98) | 8.23 | (7.65 - 8.83) | 98.00 | (97.79 - 98.19) | 2.19 | (2.09 - 2.30) | 0.50 | (0.46 -0.54) |
| Raised CRP | 10,881/30,769 | 68.46 | (65.67 - 71.15) | 65.90 | (65.36 - 66.44) | 7.14 | (6.66 - 7.64) | 98.20 | (98.01 - 98.38) | 2.01 | (1.92 - 2.10) | 0.48 | (0.44 -0.52) |
| Raised WBC count | 6,138/77,380 | 14.66 | (13.38 - 16.02) | 92.32 | (92.13 - 92.51) | 6.76 | (6.15 - 7.42) | 96.60 | (96.46 - 96.73) | 1.91 | (1.74 - 2.09) | 0.93 | (0.91 -0.94) |
| Raised creatinine | 6,636/84,299 | 14.32 | (13.09 - 15.61) | 92.37 | (92.19 - 92.55) | 6.59 | (6.00 - 7.21) | 96.63 | (96.5 - 96.76) | 1.88 | (1.72 - 2.05) | 0.93 | (0.91 -0.94) |
| Raised ferritin | 1,272/9,451 | 17.13 | (13.70 - 21.02) | 86.72 | (86.00 - 87.41) | 5.82 | (4.60 - 7.25) | 96.30 | (95.84 - 96.72) | 1.29 | (1.04 - 1.60) | 0.80 | (0.74 -0.86) |
| Raised AST | 1,443/12,910 | 16.86 | (13.45 - 20.72) | 89.02 | (88.46 - 89.56) | 5.06 | (3.99 - 6.32) | 96.86 | (96.52 - 97.17) | 1.54 | (1.24 - 1.90) | 0.93 | (0.89 -0.97) |
| Low platelets | 3,346/77,207 | 5.82 | (4.98 - 6.75) | 95.72 | (95.57 - 95.87) | 4.90 | (4.19 - 5.69) | 96.72 | (96.59 - 96.85) | 1.36 | (1.17 - 1.58) | 0.89 | (0.88 -0.91) |
| Raised bilirubin | 6,489/77,280 | 9.96 | (8.87 - 11.14) | 91.66 | (91.46 - 91.86) | 4.24 | (3.76 - 4.76) | 96.49 | (96.35 - 96.62) | 1.19 | (1.07 - 1.34) | 0.98 | (0.97 -0.99) |
| Raised HbA1c | 10,424/16,083 | 76.90 | (73.20 - 80.31) | 35.63 | (34.87 - 36.39) | 4.18 | (3.81 - 4.58) | 97.69 | (97.26 - 98.06) | 1.19 | (1.14 - 1.25) | 0.65 | (0.56 -0.75) |
| Low WBC count | 1,826/77,380 | 1.94 | (1.47 - 2.52) | 97.62 | (97.51 - 97.73) | 3.01 | (2.28 - 3.90) | 96.60 | (96.46 - 96.73) | 0.82 | (0.63 - 1.07) | 0.93 | (0.91 -0.94) |
| Raised ALT | 17,060/68,928 | 19.32 | (17.78 - 20.93) | 75.05 | (74.72 - 75.38) | 2.81 | (2.56 - 3.07) | 96.14 | (95.97 - 96.31) | 0.77 | (0.71 - 0.84) | 1.08 | (1.05 -1.10) |
| FEMALES |  |  |  |  |  |  |  |  |  |  |  |  |  |
| **Any blood test abnormality** | 86,208/174,468 | 71.27 | (69.81 - 72.71) | 51.08 | (50.84 - 51.31) | 3.15 | (3.03 - 3.26) | 98.76 | (98.69 - 98.83) | 1.46 | (1.43 – 1.49) | 0.56 | (0.53 – 0.59) |
| **Any abnormality except TM** | 85,759/173,360 | 70.29 | (68.81 – 71.74) | 51.00 | (50.76 – 51.23) | 3.10 | (2.98 – 3.22) | 98.72 | (98.64 – 98.79) | 1.43 | (1.40 – 1.47) | 0.58 | (0.55 – 0.61) |
| Raised CA125 | 982/13,460 | 52.07 | (47.12 - 56.99) | 94.11 | (93.70 - 94.51) | 21.79 | (19.25 - 24.51) | 98.42 | (98.19 - 98.63) | 8.85 | (7.88 - 9.93) | 0.51 | (0.46 -0.56) |
| Low albumin | 5,191/126,572 | 13.88 | (12.66 - 15.18) | 96.13 | (96.02 - 96.24) | 7.90 | (7.18 - 8.67) | 97.90 | (97.82 - 97.98) | 3.59 | (3.27 - 3.94) | 0.90 | (0.88 -0.91) |
| Raised platelets | 8,831/146,014 | 20.11 | (18.74 - 21.54) | 94.27 | (94.15 - 94.39) | 7.35 | (6.81 - 7.91) | 98.15 | (98.07 - 98.22) | 3.51 | (3.27 - 3.77) | 0.84 | (0.82 -0.85) |
| Raised ferritin | 721/24,937 | 9.00 | (6.79 - 11.63) | 97.25 | (97.04 - 97.46) | 7.21 | (5.43 - 9.35) | 97.86 | (97.64 - 98.06) | 3.28 | (2.50 - 4.29) | 0.92 | (0.87 – 0.98) |
| Raised ALP | 11,012/126,539 | 22.59 | (21.09 - 24.14) | 91.63 | (91.47 - 91.78) | 6.08 | (5.64 - 6.54) | 98.02 | (97.93 - 98.09) | 2.70 | (2.52 - 2.89) | 0.84 | (0.83 -0.86) |
| Anaemia | 13,399/147,234 | 23.58 | (22.13 - 25.08) | 91.23 | (91.08 - 91.37) | 5.71 | (5.32 - 6.12) | 98.15 | (98.07 - 98.22) | 2.69 | (2.52 - 2.87) | 0.84 | (0.82 -0.85) |
| Raised calcium | 1,017/50,519 | 3.93 | (2.94 - 5.14) | 98.04 | (97.91 - 98.16) | 5.01 | (3.76 - 6.54) | 97.48 | (97.34 - 97.62) | 2.00 | (1.52 - 2.64) | 0.98 | (0.97 -0.99) |
| Raised creatinine | 3,377/137,510 | 5.01 | (4.29 - 5.82) | 97.61 | (97.52 - 97.69) | 4.83 | (4.13 - 5.60) | 97.70 | (97.62 - 97.78) | 2.09 | (1.80 - 2.44) | 0.97 | (0.97 -0.98) |
| Raised AST | 1,437/21,039 | 13.96 | (11.01 - 17.36) | 93.34 | (92.99 - 93.68) | 4.73 | (3.69 - 5.96) | 97.86 | (97.65 - 98.06) | 2.10 | (1.67 - 2.63) | 0.92 | (0.89 -0.96) |
| Raised CRP | 19,210/55,605 | 64.83 | (62.19 - 67.40) | 66.19 | (65.79 - 66.59) | 4.47 | (4.18 - 4.77) | 98.72 | (98.60 - 98.83) | 1.92 | (1.84 – 2.00) | 0.53 | (0.49 -0.57) |
| Raised WBC count | 10,284/146,265 | 13.63 | (12.47 - 14.87) | 93.12 | (92.99 - 93.25) | 4.28 | (3.90 - 4.69) | 97.93 | (97.85 – 98.00) | 1.98 | (1.81 - 2.17) | 0.94 | (0.92 -0.95) |
| Raised bilirubin | 4,627/125,722 | 5.69 | (4.88 - 6.58) | 96.37 | (96.26 - 96.47) | 3.63 | (3.11 - 4.21) | 97.70 | (97.61 - 97.78) | 1.57 | (1.35 - 1.82) | 0.98 | (0.97 -0.99) |
| Raised ESR | 25,598/51,030 | 71.78 | (69.22 - 74.24) | 50.39 | (49.95 - 50.83) | 3.57 | (3.34 - 3.80) | 98.59 | (98.44 - 98.73) | 1.45 | (1.40 - 1.50) | 0.56 | (0.51 -0.61) |
| Raised HbA1c | 11,503/20,141 | 76.91 | (72.90 - 80.59) | 43.38 | (42.68 - 44.07) | 3.24 | (2.93 - 3.58) | 98.70 | (98.44 - 98.93) | 1.36 | (1.29 - 1.43) | 0.53 | (0.45 -0.63) |
| Low platelets | 2,605/146,014 | 2.54 | (2.03 - 3.14) | 98.23 | (98.16 - 98.30) | 3.15 | (2.51 - 3.89) | 98.15 | (98.07 - 98.22) | 1.44 | (1.16 - 1.79) | 0.84 | (0.82 -0.85) |
| Raised ALT | 12,398/111,843 | 14.50 | (13.18 - 15.91) | 89.00 | (88.81 - 89.18) | 3.08 | (2.78 - 3.40) | 97.74 | (97.64 - 97.83) | 1.32 | (1.20 - 1.45) | 0.96 | (0.95 -0.98) |
| Low ferritin | 5,758/24,937 | 22.49 | (19.15 – 26.12) | 76.90 | (76.36 – 77.42) | 2.26 | (1.89 – 2.68) | 97.86 | (97.64 - 98.06) | 0.97 | (0.84 – 1.13) | 0.92 | (0.87 – 0.98) |
| Low WBC count | 4,736/146,265 | 2.05 | (1.59 - 2.59) | 96.74 | (96.64 - 96.83) | 1.39 | (1.08 - 1.77) | 97.93 | (97.85 – 98.00) | 0.63 | (0.49 - 0.80) | 0.94 | (0.92 -0.95) |

##### Table K: Positive predictive values (PPVs) for cancer in 12 months by sex in patients with abdominal bloating and a blood test abnormality

Red = PPV of ≥10%; Orange = PPV of ≥3% (NICE threshold for recommending cancer investigation); Yellow = PPV ≥2%. n/N = number of patients with an abnormal test/all patients tested; LR, likelihood ratio; TM, tumour markers (PSA or CA125)

| **Patients presenting with abdominal bloating** | | | | | | | | | | | | | |
| --- | --- | --- | --- | --- | --- | --- | --- | --- | --- | --- | --- | --- | --- |
|  | n/N | Sensitivity | | Specificity | | PPV abnormal result | | NPV normal result | | Positive LR | | Negative LR | |
| MALES |  |  |  |  |  |  |  |  |  |  |  |  |  |
| **Any blood test abnormality** | 5,600/9,667 | 76.06 | (70.87 – 80.74) | 42.66 | (41.66 – 43.67) | 4.14 | (3.64 – 4.70) | 98.21 | (97.75 – 98.59) | 1.33 | (1.24 – 1.42) | 0.56 | (0.46 – 0.69) |
| **Any abnormality except TM** | 5,432/9,536 | 72.33 | (66.90 – 77.32) | 43.54 | (42.52 – 44.55) | 3.99 | (3.49 – 4.55) | 97.98 | (97.50 – 98.39) | 1.28 | (1.19 – 1.38) | 0.64 | (0.53 – 0.76) |
| Raised calcium | 35/2,804 | 4.95 | (1.63 - 11.18) | 98.89 | (98.42 - 99.25) | 14.29 | (4.81 - 30.26) | 96.53 | (95.78 - 97.18) | 4.46 | (1.77 - 11.26) | 0.96 | (0.92 - 1.01) |
| Raised platelets | 175/7,603 | 8.24 | (5.17 - 12.31) | 97.90 | (97.55 - 98.22) | 12.00 | (7.58 - 17.76) | 96.92 | (96.48 - 97.31) | 3.93 | (2.53 - 6.09) | 0.92 | (0.87 – 0.97) |
| Low albumin | 339/7,614 | 16.00 | (11.68 - 21.14) | 95.94 | (95.46 - 96.38) | 11.80 | (8.56 - 15.72) | 97.11 | (96.70 - 97.49) | 3.94 | (2.90 - 5.35) | 0.88 | (0.83 - 0.92) |
| Raised PSA | 329/1,270 | 47.89 | (35.88 - 60.08) | 75.40 | (72.86 - 77.81) | 10.33 | (7.26 - 14.14) | 96.07 | (94.62 - 97.22) | 1.95 | (1.50 - 2.53) | 0.69 | (0.55 - 0.87) |
| Anaemia | 887/7,649 | 29.69 | (24.16 - 35.69) | 89.03 | (88.30 - 89.73) | 8.57 | (6.81 - 10.61) | 97.34 | (96.93 - 97.71) | 2.71 | (2.22 - 3.30) | 0.79 | (0.73 - 0.86) |
| Raised ALP | 640/7,601 | 20.08 | (15.29 - 25.60) | 91.97 | (91.33 - 92.59) | 7.81 | (5.85 - 10.17) | 97.14 | (96.72 - 97.52) | 2.50 | (1.93 - 3.24) | 0.87 | (0.82 - 0.93) |
| Raised WBC count | 436/7,605 | 13.23 | (9.34 - 17.99) | 94.53 | (93.98 - 95.04) | 7.80 | (5.46 - 10.73) | 96.87 | (96.43 – 97.27) | 2.42 | (1.74 - 3.35) | 0.92 | (0.88 – 0.97) |
| Raised CRP | 847/2,879 | 67.37 | (56.98 - 76.64) | 71.88 | (70.16 - 73.54) | 7.56 | (5.87 - 9.55) | 98.47 | (97.84 - 98.96) | 2.40 | (2.06 - 2.79) | 0.45 | (0.34 - 0.61) |
| Raised AST | 147/1,255 | 24.44 | (12.88 - 39.54) | 88.76 | (86.84 - 90.49) | 7.48 | (3.79 - 12.99) | 96.93 | (95.74 - 97.87) | 2.17 | (1.27 - 3.72) | 0.85 | (0.72 - 1.01) |
| Raised ESR | 789/2,892 | 58.62 | (47.55 - 69.08) | 73.69 | (72.02 - 75.31) | 6.46 | (4.85 - 8.41) | 98.29 | (97.64 - 98.80) | 2.23 | (1.85 - 2.69) | 0.56 | (0.44 - 0.72) |
| Raised creatinine | 682/8,262 | 13.01 | (9.23 - 17.63) | 91.91 | (91.29 - 92.49) | 5.13 | (3.60 - 7.07) | 96.91 | (96.50 - 97.29) | 1.61 | (1.17 - 2.21) | 0.95 | (0.90 - 0.99) |
| Raised bilirubin | 632/7,614 | 13.01 | (9.07 - 17.86) | 91.86 | (91.21 - 92.47) | 5.06 | (3.49 - 7.07) | 96.93 | (96.50 - 97.33) | 1.60 | (1.15 - 2.23) | 0.95 | (0.90 - 0.99) |
| Low platelets | 425/7,603 | 7.06 | (4.24 - 10.93) | 94.46 | (93.91 - 94.97) | 4.24 | (2.53 - 6.61) | 96.92 | (96.48 - 97.31) | 1.27 | (0.81 - 2.01) | 0.92 | (0.87 – 0.97) |
| Low ferritin | 128/1,219 | 17.24 | (5.85 - 35.77) | 89.66 | (87.79 - 91.34) | 3.91 | (1.28 - 8.88) | 97.80 | (96.65 - 98.63) | 1.67 | (0.74 - 3.77) | 0.92 | (0.74 - 1.16) |
| Raised HbA1c | 1,195/1,789 | 79.25 | (65.89 - 89.16) | 33.58 | (31.36 - 35.86) | 3.51 | (2.54 - 4.72) | 98.15 | (96.71 - 99.07) | 1.19 | (1.04 - 1.37) | 0.62 | (0.36 - 1.05) |
| Low WBC count | 204/7,605 | 1.95 | (0.63 - 4.48) | 97.29 | (96.89 - 97.65) | 2.45 | (0.80 - 5.63) | 96.87 | (96.43 – 97.27) | 0.72 | (0.30 - 1.73) | 0.92 | (0.88 – 0.97) |
| Raised ferritin | 138/1,219 | 10.34 | (2.19 - 27.35) | 88.66 | (86.72 - 90.40) | 2.17 | (0.45 - 6.22) | 97.80 | (96.65 - 98.63) | 0.91 | (0.31 - 2.69) | 0.92 | (0.74 - 1.16) |
| Raised ALT | 1,680/6,739 | 14.76 | (10.26 - 20.29) | 74.74 | (73.67 - 75.79) | 1.85 | (1.26 - 2.61) | 96.46 | (95.92 - 96.95) | 0.58 | (0.42 - 0.81) | 1.14 | (1.08 - 1.21) |
| FEMALES |  |  |  |  |  |  |  |  |  |  |  |  |  |
| **Any blood test abnormality** | 12,291/26,966 | 70.47 | (66.69 – 74.06) | 55.00 | (54.40 – 55.60) | 3.51 | (3.20 – 3.86) | 98.77 | (98.56 – 98.94) | 1.57 | (1.49 – 1.65) | 0.54 | (0.47 – 0.61) |
| **Any abnormality except TM** | 12,106/26,384 | 67.17 | (63.25 – 70.92) | 54.61 | (54.00 – 55.22) | 3.33 | (3.02 – 3.66) | 98.62 | (98.42 – 98.81) | 1.48 | (1.40 – 1.57) | 0.60 | (0.54 – 0.67) |
| Raised CA125 | 421/6,972 | 55.91 | (48.46 - 63.17) | 95.33 | (94.80 - 95.82) | 24.70 | (20.65 - 29.11) | 98.75 | (98.45 – 99.00) | 11.97 | (10.13 - 14.14) | 0.46 | (0.39 - 0.54) |
| Low albumin | 690/20,131 | 16.60 | (13.41 - 20.20) | 96.90 | (96.65 - 97.14) | 11.74 | (9.43 - 14.38) | 97.91 | (97.70 - 98.10) | 5.35 | (4.32 - 6.63) | 0.86 | (0.83 - 0.90) |
| Raised platelets | 1,090/22,740 | 21.46 | (17.96 - 25.29) | 95.59 | (95.31 - 95.85) | 10.00 | (8.28 - 11.94) | 98.18 | (97.99 - 98.35) | 4.86 | (4.07 - 5.81) | 0.81 | (0.77 – 0.85) |
| Raised ferritin | 88/4,742 | 6.82 | (2.54 - 14.25) | 98.24 | (97.82 - 98.60) | 6.82 | (2.54 - 14.25) | 98.18 | (97.69 - 98.59) | 3.87 | (1.74 - 8.63) | 0.98 | (0.87 - 1.11) |
| Raised ALP | 1,403/19,954 | 17.47 | (14.16 - 21.19) | 93.22 | (92.86 - 93.57) | 5.92 | (4.74 - 7.28) | 97.89 | (97.67 - 98.09) | 2.58 | (2.11 - 3.16) | 0.89 | (0.85 - 0.92) |
| Raised WBC count | 1,051/22,728 | 11.94 | (9.26 - 15.07) | 95.54 | (95.26 - 95.81) | 5.80 | (4.47 - 7.39) | 97.88 | (97.67 - 98.07) | 2.68 | (2.10 - 3.42) | 0.94 | (0.91 – 0.98) |
| Raised CRP | 2,262/8,529 | 64.22 | (57.22 - 70.79) | 74.40 | (73.45 - 75.34) | 5.79 | (4.86 - 6.83) | 98.84 | (98.54 - 99.09) | 2.51 | (2.25 - 2.80) | 0.48 | (0.40 - 0.58) |
| Anaemia | 1,593/22,861 | 17.45 | (14.26 - 21.03) | 93.27 | (92.93 - 93.60) | 5.59 | (4.51 - 6.83) | 98.02 | (97.82 - 98.20) | 2.59 | (2.13 - 3.15) | 0.89 | (0.85 - 0.92) |
| Raised creatinine | 384/21,777 | 3.76 | (2.31 - 5.75) | 98.29 | (98.10 - 98.46) | 5.21 | (3.21 - 7.93) | 97.61 | (97.39 - 97.81) | 2.19 | (1.41 - 3.41) | 0.98 | (0.96 – 1.00) |
| Raised ESR | 3,891/8,791 | 75.73 | (69.28 - 81.42) | 56.49 | (55.44 - 57.55) | 4.01 | (3.41 - 4.67) | 98.98 | (98.66 - 99.24) | 1.74 | (1.61 - 1.89) | 0.43 | (0.34 - 0.55) |
| Raised ALT | 1,521/17,785 | 11.78 | (8.90 - 15.19) | 91.53 | (91.10 - 91.94) | 3.35 | (2.51 - 4.39) | 97.65 | (97.41 - 97.88) | 1.39 | (1.07 - 1.81) | 0.96 | (0.93 – 1.00) |
| Raised AST | 179/3,154 | 8.33 | (3.12 - 17.26) | 94.39 | (93.51 - 95.17) | 3.35 | (1.24 - 7.15) | 97.78 | (97.19 - 98.28) | 1.48 | (0.68 - 3.24) | 0.97 | (0.91 - 1.04) |
| Raised HbA1c | 1,840/3,587 | 68.60 | (57.7 - 78.19) | 49.13 | (47.46 - 50.80) | 3.21 | (2.45 - 4.12) | 98.45 | (97.76 - 98.98) | 1.35 | (1.16 - 1.56) | 0.64 | (0.47 - 0.87) |
| Raised calcium | 164/8,438 | 2.33 | (0.76 - 5.34) | 98.07 | (97.75 - 98.35) | 3.05 | (1.00 - 6.97) | 97.46 | (97.10 - 97.79) | 1.20 | (0.50 - 2.90) | 1.00 | (0.98 - 1.02) |
| Raised bilirubin | 605/19,910 | 3.52 | (2.06 - 5.58) | 96.97 | (96.72 - 97.21) | 2.81 | (1.65 - 4.46) | 97.59 | (97.36 - 97.80) | 1.16 | (0.72 - 1.87) | 0.99 | (0.98 - 1.01) |
| Low platelets | 444/22,740 | 2.36 | (1.23 - 4.09) | 98.06 | (97.87 - 98.23) | 2.70 | (1.40 - 4.67) | 98.18 | (97.99 - 98.35) | 1.22 | (0.69 - 2.14) | 0.81 | (0.77 – 0.85) |
| Low ferritin | 1,026/4,742 | 18.18 | (10.76 - 27.84) | 78.30 | (77.09 - 79.48) | 1.56 | (0.89 - 2.52) | 98.18 | (97.69 - 98.59) | 0.84 | (0.54 - 1.31) | 0.98 | (0.87 - 1.11) |
| Low WBC count | 798/22,728 | 1.37 | (0.55 - 2.80) | 96.44 | (96.19 - 96.68) | 0.88 | (0.35 - 1.80) | 97.88 | (97.67 - 98.07) | 0.38 | (0.18 - 0.81) | 0.94 | (0.91 – 0.98) |

##### Table L: Positive predictive values (PPVs) for different cancer sites within 12 months by sex in tested patients with abdominal pain or bloating and a blood test abnormality: males with abdominal pain, females with abdominal pain, males with abdominal bloating, females with abdominal bloating. PPVs >10% highlighted in red, >3% highlighted in orange, >2% highlighted in yellow, >1% highlighted in blue.

| **PPVs by cancer site in males with abdominal pain** | | | | | | | | | | | | | | | | | | |
| --- | --- | --- | --- | --- | --- | --- | --- | --- | --- | --- | --- | --- | --- | --- | --- | --- | --- | --- |
| **cancer_site_final** | **Anaemia** | **Low albumin** | **Low ferritin** | **Low platelets** | **Low WBC count** | **Raised ALP** | **Raised ALT** | **Raised AST** | **Raised bilirubin** | **Raised calcium** | **Raised creatinine** | **Raised CRP** | **Raised ESR** | **Raised ferritin** | **Raised HbA1c** | **Raised platelets** | **Raised PSA** | **Raised WBC count** |
| **Urinary** | 0.82 | 0.64 | 0.32 | 0.33 | 0.05 | 0.45 | 0.13 | 0.28 | 0.20 | 0.54 | 0.95 | 0.43 | 0.40 | 0.39 | 0.24 | 0.79 | 0.51 | 0.34 |
| **Brain & CNS** | 0.01 | 0.10 | 0.00 | 0.00 | 0.00 | 0.04 | 0.03 | 0.07 | 0.05 | 0.00 | 0.05 | 0.03 | 0.04 | 0.00 | 0.00 | 0.00 | 0.06 | 0.02 |
| **CUP** | 0.74 | 1.21 | 0.21 | 0.15 | 0.00 | 1.06 | 0.22 | 0.28 | 0.37 | 1.34 | 0.51 | 0.53 | 0.64 | 0.94 | 0.27 | 0.68 | 0.33 | 0.52 |
| **Bowel** | 3.41 | 3.05 | 5.93 | 0.30 | 0.44 | 1.72 | 0.35 | 0.55 | 0.51 | 1.88 | 1.22 | 1.89 | 2.20 | 0.39 | 1.06 | 4.48 | 1.50 | 1.78 |
| **Head and Neck** | 0.07 | 0.13 | 0.00 | 0.00 | 0.00 | 0.07 | 0.03 | 0.00 | 0.00 | 0.00 | 0.05 | 0.07 | 0.04 | 0.00 | 0.03 | 0.08 | 0.03 | 0.03 |
| **Thyroid** | 0.01 | 0.00 | 0.00 | 0.00 | 0.05 | 0.01 | 0.01 | 0.00 | 0.02 | 0.00 | 0.00 | 0.00 | 0.00 | 0.00 | 0.00 | 0.00 | 0.00 | 0.00 |
| **Lymphoma** | 0.69 | 0.77 | 0.21 | 0.45 | 0.38 | 0.39 | 0.11 | 0.21 | 0.18 | 0.54 | 0.39 | 0.60 | 0.47 | 0.31 | 0.15 | 0.79 | 0.27 | 0.36 |
| **Leukaemia** | 0.28 | 0.07 | 0.00 | 0.45 | 0.33 | 0.04 | 0.05 | 0.00 | 0.05 | 0.27 | 0.14 | 0.09 | 0.11 | 0.24 | 0.02 | 0.08 | 0.00 | 0.39 |
| **Other haem** | 0.07 | 0.03 | 0.00 | 0.21 | 0.05 | 0.01 | 0.02 | 0.00 | 0.03 | 0.00 | 0.02 | 0.01 | 0.00 | 0.08 | 0.01 | 0.00 | 0.00 | 0.05 |
| **Liver** | 0.40 | 1.01 | 0.11 | 0.48 | 0.11 | 1.04 | 0.35 | 0.83 | 0.59 | 0.81 | 0.29 | 0.31 | 0.40 | 0.16 | 0.13 | 0.38 | 0.15 | 0.16 |
| **Lung** | 0.82 | 1.64 | 0.11 | 0.33 | 0.05 | 1.01 | 0.22 | 0.35 | 0.26 | 2.15 | 0.69 | 0.77 | 0.94 | 0.86 | 0.39 | 1.39 | 0.57 | 0.83 |
| **Melanoma** | 0.02 | 0.03 | 0.00 | 0.03 | 0.00 | 0.06 | 0.01 | 0.00 | 0.02 | 0.00 | 0.03 | 0.06 | 0.04 | 0.00 | 0.06 | 0.00 | 0.06 | 0.00 |
| **Myeloma** | 0.19 | 0.20 | 0.11 | 0.12 | 0.27 | 0.09 | 0.02 | 0.00 | 0.03 | 0.54 | 0.11 | 0.03 | 0.06 | 0.39 | 0.01 | 0.00 | 0.12 | 0.05 |
| **Oesophago-gastric** | 1.27 | 1.27 | 1.17 | 0.36 | 0.22 | 0.80 | 0.23 | 0.62 | 0.39 | 0.54 | 0.66 | 0.52 | 0.87 | 0.24 | 0.39 | 1.20 | 0.36 | 0.70 |
| **Other** | 0.48 | 0.70 | 0.53 | 0.18 | 0.00 | 0.58 | 0.18 | 0.49 | 0.29 | 0.54 | 0.24 | 0.26 | 0.28 | 0.24 | 0.14 | 0.56 | 0.21 | 0.34 |
| **Pancreatic** | 0.69 | 0.97 | 0.32 | 0.81 | 0.33 | 1.49 | 0.51 | 0.90 | 0.71 | 0.54 | 0.23 | 0.71 | 0.83 | 0.71 | 0.71 | 0.72 | 0.48 | 0.65 |
| **Prostate** | 0.99 | 0.97 | 0.53 | 0.72 | 0.60 | 1.12 | 0.31 | 0.42 | 0.51 | 1.08 | 0.98 | 0.71 | 0.82 | 0.86 | 0.54 | 0.79 | 7.57 | 0.47 |
| **Sarcoma** | 0.08 | 0.07 | 0.00 | 0.00 | 0.05 | 0.04 | 0.02 | 0.07 | 0.03 | 0.00 | 0.03 | 0.06 | 0.08 | 0.00 | 0.01 | 0.08 | 0.03 | 0.05 |
| **Testicular** | 0.02 | 0.03 | 0.00 | 0.00 | 0.05 | 0.03 | 0.02 | 0.00 | 0.02 | 0.00 | 0.00 | 0.05 | 0.02 | 0.00 | 0.01 | 0.08 | 0.03 | 0.00 |

| **PPVs by cancer site in females with abdominal pain** | | | | | | | | | | | | | | | | | | |
| --- | --- | --- | --- | --- | --- | --- | --- | --- | --- | --- | --- | --- | --- | --- | --- | --- | --- | --- |
| **cancer_site_final** | **Anaemia** | **Low albumin** | **Low ferritin** | **Low platelets** | **Low WBC count** | **Raised ALP** | **Raised ALT** | **Raised AST** | **Raised bilirubin** | **Raised CA125** | **Raised calcium** | **Raised creatinine** | **Raised CRP** | **Raised ESR** | **Raised ferritin** | **Raised HbA1c** | **Raised platelets** | **Raised WBC count** |
| **Urinary** | 0.25 | 0.37 | 0.03 | 0.08 | 0.00 | 0.20 | 0.06 | 0.07 | 0.11 | 0.31 | 0.39 | 0.59 | 0.15 | 0.16 | 0.14 | 0.11 | 0.22 | 0.14 |
| **Brain & CNS** | 0.02 | 0.02 | 0.03 | 0.04 | 0.00 | 0.01 | 0.02 | 0.00 | 0.00 | 0.00 | 0.00 | 0.03 | 0.01 | 0.03 | 0.00 | 0.04 | 0.01 | 0.02 |
| **Breast** | 0.25 | 0.29 | 0.26 | 0.19 | 0.30 | 0.34 | 0.24 | 0.21 | 0.28 | 0.20 | 0.20 | 0.30 | 0.21 | 0.23 | 0.55 | 0.37 | 0.34 | 0.28 |
| **CUP** | 0.39 | 0.83 | 0.05 | 0.19 | 0.04 | 0.70 | 0.30 | 0.28 | 0.28 | 1.32 | 0.98 | 0.62 | 0.40 | 0.26 | 0.83 | 0.17 | 0.60 | 0.41 |
| **Cervical** | 0.11 | 0.13 | 0.12 | 0.12 | 0.04 | 0.04 | 0.08 | 0.00 | 0.04 | 0.10 | 0.00 | 0.03 | 0.12 | 0.06 | 0.28 | 0.03 | 0.14 | 0.16 |
| **Bowel** | 2.31 | 2.49 | 1.11 | 0.08 | 0.15 | 1.17 | 0.44 | 0.63 | 0.32 | 2.04 | 0.88 | 0.89 | 1.16 | 0.96 | 1.11 | 0.63 | 2.74 | 1.07 |
| **Head and Neck** | 0.02 | 0.00 | 0.00 | 0.00 | 0.00 | 0.02 | 0.01 | 0.07 | 0.02 | 0.00 | 0.00 | 0.03 | 0.01 | 0.00 | 0.00 | 0.01 | 0.01 | 0.02 |
| **Thyroid** | 0.00 | 0.00 | 0.00 | 0.00 | 0.00 | 0.00 | 0.01 | 0.00 | 0.02 | 0.00 | 0.00 | 0.00 | 0.01 | 0.01 | 0.00 | 0.00 | 0.01 | 0.00 |
| **Lymphoma** | 0.28 | 0.44 | 0.00 | 0.58 | 0.17 | 0.24 | 0.13 | 0.21 | 0.06 | 0.41 | 0.39 | 0.50 | 0.20 | 0.20 | 0.28 | 0.10 | 0.18 | 0.17 |
| **Leukaemia** | 0.07 | 0.04 | 0.00 | 0.27 | 0.11 | 0.03 | 0.02 | 0.00 | 0.04 | 0.00 | 0.10 | 0.00 | 0.04 | 0.02 | 0.00 | 0.03 | 0.00 | 0.15 |
| **Other haem** | 0.01 | 0.00 | 0.00 | 0.04 | 0.04 | 0.00 | 0.00 | 0.00 | 0.00 | 0.00 | 0.00 | 0.06 | 0.01 | 0.00 | 0.00 | 0.00 | 0.00 | 0.03 |
| **Liver** | 0.06 | 0.21 | 0.00 | 0.23 | 0.00 | 0.40 | 0.19 | 0.56 | 0.41 | 0.20 | 0.29 | 0.12 | 0.10 | 0.09 | 0.00 | 0.03 | 0.10 | 0.08 |
| **Lung** | 0.33 | 0.60 | 0.05 | 0.00 | 0.08 | 0.48 | 0.21 | 0.49 | 0.09 | 0.51 | 0.39 | 0.38 | 0.34 | 0.25 | 0.69 | 0.28 | 0.54 | 0.49 |
| **Melanoma** | 0.03 | 0.00 | 0.03 | 0.08 | 0.06 | 0.04 | 0.02 | 0.00 | 0.02 | 0.10 | 0.00 | 0.09 | 0.03 | 0.02 | 0.00 | 0.04 | 0.05 | 0.03 |
| **Myeloma** | 0.13 | 0.15 | 0.00 | 0.27 | 0.06 | 0.05 | 0.03 | 0.00 | 0.00 | 0.10 | 0.29 | 0.09 | 0.06 | 0.04 | 0.14 | 0.05 | 0.05 | 0.04 |
| **Oesophago-gastric** | 0.30 | 0.37 | 0.24 | 0.04 | 0.02 | 0.28 | 0.11 | 0.28 | 0.15 | 0.51 | 0.29 | 0.27 | 0.21 | 0.17 | 0.42 | 0.14 | 0.37 | 0.19 |
| **Other** | 0.25 | 0.35 | 0.09 | 0.12 | 0.06 | 0.32 | 0.20 | 0.35 | 0.35 | 0.81 | 0.00 | 0.15 | 0.17 | 0.13 | 0.69 | 0.10 | 0.32 | 0.21 |
| **Ovarian** | 0.42 | 1.06 | 0.09 | 0.12 | 0.15 | 0.45 | 0.21 | 0.42 | 0.15 | 11.81 | 0.29 | 0.36 | 0.58 | 0.41 | 0.55 | 0.30 | 1.03 | 0.34 |
| **Pancreatic** | 0.31 | 0.39 | 0.10 | 0.61 | 0.02 | 1.14 | 0.71 | 1.11 | 1.19 | 1.93 | 0.39 | 0.21 | 0.47 | 0.37 | 1.11 | 0.63 | 0.36 | 0.33 |
| **Sarcoma** | 0.04 | 0.08 | 0.00 | 0.08 | 0.04 | 0.06 | 0.03 | 0.00 | 0.02 | 0.51 | 0.00 | 0.03 | 0.08 | 0.04 | 0.14 | 0.04 | 0.09 | 0.04 |
| **Uterine** | 0.12 | 0.10 | 0.03 | 0.04 | 0.02 | 0.12 | 0.06 | 0.07 | 0.06 | 0.92 | 0.10 | 0.09 | 0.11 | 0.08 | 0.28 | 0.10 | 0.18 | 0.09 |
| **Vulval** | 0.01 | 0.00 | 0.00 | 0.00 | 0.02 | 0.01 | 0.00 | 0.00 | 0.00 | 0.00 | 0.00 | 0.00 | 0.01 | 0.00 | 0.00 | 0.02 | 0.01 | 0.01 |

| **PPVs by cancer site in males with abdominal bloating** | | | | | | | | | | | | | | | | | | |
| --- | --- | --- | --- | --- | --- | --- | --- | --- | --- | --- | --- | --- | --- | --- | --- | --- | --- | --- |
| **cancer_site_final** | **Anaemia** | **Low albumin** | **Low ferritin** | **Low platelets** | **Low WBC count** | **Raised ALP** | **Raised ALT** | **Raised AST** | **Raised bilirubin** | **Raised calcium** | **Raised creatinine** | **Raised CRP** | **Raised ESR** | **Raised ferritin** | **Raised HbA1c** | **Raised platelets** | **Raised PSA** | **Raised WBC count** |
| **Urinary** | 0.34 | 0.29 | 0.00 | 0.24 | 0.00 | 0.47 | 0.18 | 0.00 | 0.16 | 0.00 | 0.59 | 0.59 | 0.38 | 0.00 | 0.33 | 0.00 | 0.61 | 0.23 |
| **Brain & CNS** | 0.23 | 0.29 | 0.00 | 0.00 | 0.00 | 0.31 | 0.06 | 0.00 | 0.32 | 0.00 | 0.00 | 0.00 | 0.00 | 0.00 | 0.00 | 0.00 | 0.00 | 0.23 |
| **CUP** | 0.68 | 0.59 | 0.00 | 0.00 | 0.00 | 0.47 | 0.30 | 0.68 | 0.16 | 2.86 | 0.44 | 0.59 | 0.38 | 0.72 | 0.25 | 1.71 | 0.30 | 0.69 |
| **Bowel** | 1.80 | 1.77 | 1.56 | 0.00 | 0.00 | 0.78 | 0.24 | 0.68 | 0.47 | 0.00 | 0.73 | 1.53 | 1.52 | 0.00 | 0.67 | 2.86 | 1.22 | 2.52 |
| **Head and Neck** | 0.00 | 0.00 | 0.00 | 0.00 | 0.00 | 0.00 | 0.00 | 0.00 | 0.16 | 2.86 | 0.15 | 0.12 | 0.00 | 0.00 | 0.00 | 0.00 | 0.00 | 0.00 |
| **Thyroid** | 0.00 | 0.00 | 0.00 | 0.00 | 0.00 | 0.00 | 0.00 | 0.00 | 0.00 | 0.00 | 0.00 | 0.00 | 0.00 | 0.00 | 0.00 | 0.00 | 0.00 | 0.00 |
| **Lymphoma** | 0.79 | 0.88 | 0.00 | 0.71 | 0.49 | 0.31 | 0.06 | 0.00 | 0.32 | 0.00 | 0.44 | 0.83 | 0.51 | 0.72 | 0.08 | 1.14 | 0.30 | 0.23 |
| **Leukaemia** | 0.11 | 0.00 | 0.00 | 0.00 | 0.00 | 0.16 | 0.06 | 0.00 | 0.00 | 0.00 | 0.00 | 0.00 | 0.00 | 0.00 | 0.00 | 0.00 | 0.00 | 0.69 |
| **Other haem** | 0.11 | 0.29 | 0.00 | 0.00 | 0.00 | 0.00 | 0.00 | 0.00 | 0.00 | 0.00 | 0.00 | 0.12 | 0.00 | 0.00 | 0.00 | 0.57 | 0.00 | 0.23 |
| **Liver** | 1.13 | 2.65 | 0.00 | 1.41 | 0.98 | 1.88 | 0.36 | 0.68 | 1.42 | 0.00 | 0.29 | 0.35 | 0.51 | 0.00 | 0.33 | 0.57 | 0.00 | 0.92 |
| **Lung** | 0.34 | 1.18 | 0.00 | 0.00 | 0.00 | 0.31 | 0.06 | 0.00 | 0.47 | 2.86 | 0.29 | 0.59 | 0.76 | 0.00 | 0.25 | 1.71 | 0.00 | 0.46 |
| **Melanoma** | 0.11 | 0.00 | 0.00 | 0.00 | 0.00 | 0.00 | 0.00 | 0.00 | 0.00 | 0.00 | 0.00 | 0.00 | 0.00 | 0.00 | 0.00 | 0.00 | 0.00 | 0.00 |
| **Myeloma** | 0.11 | 0.88 | 0.00 | 0.00 | 0.00 | 0.31 | 0.00 | 0.00 | 0.00 | 2.86 | 0.00 | 0.00 | 0.13 | 0.00 | 0.08 | 0.00 | 0.30 | 0.00 |
| **Oesophago-gastric** | 1.01 | 0.88 | 1.56 | 0.24 | 0.00 | 0.63 | 0.18 | 0.68 | 0.16 | 0.00 | 0.44 | 0.94 | 0.51 | 0.00 | 0.08 | 1.14 | 0.61 | 0.69 |
| **Other** | 0.00 | 0.29 | 0.24 | 0.00 | 0.00 | 0.00 | 0.00 | 0.00 | 0.00 | 0.00 | 0.29 | 0.12 | 0.13 | 0.00 | 0.08 | 1.14 | 0.30 | 0.23 |
| **Pancreatic** | 0.79 | 1.18 | 0.00 | 0.94 | 0.49 | 1.25 | 0.30 | 3.40 | 0.95 | 0.00 | 0.73 | 0.83 | 0.76 | 0.72 | 0.92 | 0.00 | 0.61 | 0.00 |
| **Prostate** | 0.90 | 0.00 | 0.78 | 0.47 | 0.49 | 0.94 | 0.06 | 1.36 | 0.47 | 2.86 | 0.73 | 0.71 | 0.89 | 0.00 | 0.42 | 0.57 | 6.08 | 0.69 |
| **Sarcoma** | 0.11 | 0.29 | 0.00 | 0.00 | 0.00 | 0.00 | 0.00 | 0.00 | 0.00 | 0.00 | 0.00 | 0.12 | 0.00 | 0.00 | 0.00 | 0.57 | 0.00 | 0.00 |
| **Testicular** | 0.00 | 0.29 | 0.00 | 0.00 | 0.00 | 0.00 | 0.00 | 0.00 | 0.00 | 0.00 | 0.00 | 0.12 | 0.00 | 0.00 | 0.00 | 0.00 | 0.00 | 0.00 |

| **PPVs by cancer site in females with abdominal bloating** | | | | | | | | | | | | | | | | | | |
| --- | --- | --- | --- | --- | --- | --- | --- | --- | --- | --- | --- | --- | --- | --- | --- | --- | --- | --- |
| **cancer_site_final** | **Anaemia** | **Low albumin** | **Low ferritin** | **Low platelets** | **Low WBC count** | **Raised ALP** | **Raised ALT** | **Raised AST** | **Raised bilirubin** | **Raised CA125** | **Raised calcium** | **Raised creatinine** | **Raised CRP** | **Raised ESR** | **Raised ferritin** | **Raised HbA1c** | **Raised platelets** | **Raised WBC count** |
| **Urinary** | 0.13 | 0.29 | 0.00 | 0.45 | 0.13 | 0.00 | 0.07 | 0.00 | 0.00 | 0.24 | 0.00 | 0.00 | 0.22 | 0.13 | 0.00 | 0.11 | 0.09 | 0.19 |
| **Brain & CNS** | 0.00 | 0.00 | 0.00 | 0.00 | 0.00 | 0.00 | 0.00 | 0.00 | 0.00 | 0.00 | 0.00 | 0.00 | 0.00 | 0.05 | 0.00 | 0.00 | 0.00 | 0.00 |
| **Breast** | 0.31 | 0.43 | 0.49 | 0.23 | 0.00 | 0.43 | 0.13 | 0.00 | 0.66 | 0.71 | 0.61 | 0.26 | 0.18 | 0.21 | 0.00 | 0.38 | 0.37 | 0.10 |
| **CUP** | 0.63 | 1.30 | 0.10 | 0.00 | 0.00 | 0.78 | 0.26 | 0.56 | 0.66 | 1.19 | 0.61 | 1.04 | 0.35 | 0.23 | 1.14 | 0.27 | 0.73 | 0.95 |
| **Cervical** | 0.00 | 0.00 | 0.19 | 0.45 | 0.00 | 0.07 | 0.07 | 0.00 | 0.33 | 0.24 | 0.00 | 0.00 | 0.09 | 0.13 | 0.00 | 0.16 | 0.00 | 0.19 |
| **Bowel** | 1.26 | 1.88 | 0.49 | 0.00 | 0.13 | 0.57 | 0.33 | 0.00 | 0.17 | 1.19 | 0.00 | 0.52 | 0.84 | 0.75 | 0.00 | 0.27 | 1.56 | 0.76 |
| **Head and Neck** | 0.00 | 0.00 | 0.00 | 0.00 | 0.00 | 0.00 | 0.00 | 0.00 | 0.00 | 0.00 | 0.00 | 0.00 | 0.00 | 0.03 | 0.00 | 0.00 | 0.00 | 0.10 |
| **Thyroid** | 0.00 | 0.00 | 0.00 | 0.00 | 0.00 | 0.00 | 0.00 | 0.00 | 0.00 | 0.00 | 0.00 | 0.00 | 0.00 | 0.00 | 0.00 | 0.00 | 0.00 | 0.00 |
| **Lymphoma** | 0.25 | 0.43 | 0.10 | 0.00 | 0.00 | 0.14 | 0.00 | 0.56 | 0.00 | 0.48 | 0.00 | 0.78 | 0.27 | 0.10 | 0.00 | 0.11 | 0.00 | 0.10 |
| **Leukaemia** | 0.06 | 0.00 | 0.00 | 0.45 | 0.13 | 0.00 | 0.00 | 0.00 | 0.00 | 0.00 | 0.00 | 0.00 | 0.00 | 0.00 | 0.00 | 0.11 | 0.09 | 0.10 |
| **Other haem** | 0.13 | 0.14 | 0.00 | 0.45 | 0.13 | 0.00 | 0.00 | 0.00 | 0.00 | 0.00 | 0.00 | 0.26 | 0.04 | 0.00 | 0.00 | 0.00 | 0.00 | 0.00 |
| **Liver** | 0.25 | 0.14 | 0.00 | 0.23 | 0.00 | 0.50 | 0.33 | 0.56 | 0.50 | 0.24 | 0.00 | 0.26 | 0.04 | 0.05 | 0.00 | 0.05 | 0.00 | 0.19 |
| **Lung** | 0.38 | 0.72 | 0.10 | 0.23 | 0.13 | 0.57 | 0.20 | 0.00 | 0.17 | 0.48 | 0.61 | 0.00 | 0.40 | 0.26 | 0.00 | 0.22 | 0.55 | 0.57 |
| **Melanoma** | 0.00 | 0.00 | 0.00 | 0.00 | 0.00 | 0.07 | 0.00 | 0.00 | 0.00 | 0.00 | 0.00 | 0.26 | 0.04 | 0.03 | 0.00 | 0.00 | 0.00 | 0.00 |
| **Myeloma** | 0.06 | 0.00 | 0.00 | 0.00 | 0.13 | 0.07 | 0.00 | 0.00 | 0.00 | 0.24 | 0.61 | 0.00 | 0.00 | 0.08 | 0.00 | 0.00 | 0.00 | 0.00 |
| **Oesophago-gastric** | 0.13 | 0.14 | 0.00 | 0.00 | 0.00 | 0.21 | 0.20 | 0.00 | 0.00 | 0.24 | 0.61 | 0.00 | 0.18 | 0.10 | 0.00 | 0.11 | 0.28 | 0.00 |
| **Other** | 0.13 | 0.29 | 0.00 | 0.23 | 0.00 | 0.14 | 0.13 | 0.56 | 0.17 | 0.48 | 0.00 | 0.26 | 0.09 | 0.13 | 0.00 | 0.00 | 0.18 | 0.10 |
| **Ovarian** | 1.19 | 5.51 | 0.10 | 0.00 | 0.13 | 1.64 | 0.99 | 1.12 | 0.00 | 15.91 | 0.00 | 1.04 | 2.48 | 1.21 | 4.55 | 0.71 | 5.32 | 2.09 |
| **Pancreatic** | 0.25 | 0.14 | 0.00 | 0.00 | 0.00 | 0.50 | 0.53 | 0.00 | 0.17 | 0.71 | 0.00 | 0.26 | 0.31 | 0.28 | 0.00 | 0.49 | 0.37 | 0.19 |
| **Sarcoma** | 0.31 | 0.29 | 0.00 | 0.00 | 0.00 | 0.07 | 0.13 | 0.00 | 0.00 | 1.66 | 0.00 | 0.00 | 0.13 | 0.18 | 1.14 | 0.11 | 0.28 | 0.10 |
| **Uterine** | 0.13 | 0.00 | 0.00 | 0.00 | 0.00 | 0.14 | 0.00 | 0.00 | 0.00 | 0.71 | 0.00 | 0.26 | 0.13 | 0.08 | 0.00 | 0.11 | 0.18 | 0.10 |
| **Vulval** | 0.00 | 0.00 | 0.00 | 0.00 | 0.00 | 0.00 | 0.00 | 0.00 | 0.00 | 0.00 | 0.00 | 0.00 | 0.00 | 0.00 | 0.00 | 0.00 | 0.00 | 0.00 |

##### Table M: Risk ratios (RR) comparing 1 year cancer risk in patient with abdominal pain and a normal versus abnormal test result for 19 different blood test abnormalities. *adjusted for age

|  | Crude RR | | | Adjusted RR* | | | Age-stratified RR  (30-39 years) | | | Age-stratified RR  (40-49 years) | | | Age-stratified RR  (50-59 years) | | | Age-stratified RR  (60-69 years) | | | Age-stratified RR  (70-79 years) | | | Age-stratified RR  (≥80 years) | | |
| --- | --- | --- | --- | --- | --- | --- | --- | --- | --- | --- | --- | --- | --- | --- | --- | --- | --- | --- | --- | --- | --- | --- | --- | --- |
| Blood test abnormality | **RR** | **95%CI** | | **RR** | **95% CI** | | **RR** | **95% CI** | | **RR** | **95% CI** | | **RR** | **95% CI** | | **RR** | **95% CI** | | **RR** | **95% CI** | | **RR** | **95% CI** | |
| MALES | | | | | | | | | | | | | | | | | | | | | | | | |
| Low albumin | 4.06 | 3.67 | 4.49 | 2.53 | 2.28 | 2.81 | 10.45 | 3.80 | 28.75 | 2.48 | 1.02 | 6.01 | 4.00 | 2.91 | 5.51 | 3.21 | 2.64 | 3.90 | 2.32 | 1.93 | 2.77 | 1.96 | 1.62 | 2.36 |
| Raised ALT | 0.73 | 0.66 | 0.80 | 1.11 | 1.00 | 1.22 | 0.63 | 0.30 | 1.33 | 0.88 | 0.59 | 1.31 | 0.77 | 0.60 | 0.98 | 1.14 | 0.96 | 1.35 | 1.19 | 0.98 | 1.43 | 1.82 | 1.44 | 2.30 |
| Raised AST | 1.61 | 1.26 | 2.06 | 1.87 | 1.47 | 2.38 | 0.82 | 0.10 | 6.55 | 1.40 | 0.48 | 4.10 | 1.37 | 0.70 | 2.67 | 2.20 | 1.47 | 3.28 | 1.64 | 1.00 | 2.70 | 2.59 | 1.53 | 4.37 |
| Raised bilirubin | 1.21 | 1.07 | 1.36 | 1.14 | 1.01 | 1.29 | 1.46 | 0.58 | 3.71 | 1.44 | 0.83 | 2.50 | 0.99 | 0.69 | 1.42 | 1.29 | 1.04 | 1.60 | 1.11 | 0.89 | 1.37 | 1.01 | 0.77 | 1.33 |
| Raised CRP | 3.97 | 3.51 | 4.49 | 2.98 | 2.62 | 3.37 | 3.45 | 1.55 | 7.68 | 6.17 | 3.69 | 10.32 | 3.49 | 2.59 | 4.70 | 3.06 | 2.45 | 3.82 | 2.43 | 1.94 | 3.05 | 2.36 | 1.74 | 3.19 |
| Raised ESR | 4.12 | 3.64 | 4.65 | 2.68 | 2.35 | 3.04 | 4.92 | 2.00 | 12.06 | 5.94 | 3.61 | 9.78 | 3.01 | 2.21 | 4.11 | 2.61 | 2.10 | 3.23 | 2.28 | 1.82 | 2.86 | 2.16 | 1.59 | 2.95 |
| Raised HbA1c | 1.81 | 1.49 | 2.19 | 1.30 | 1.08 | 1.58 | 0.00 | 0.00 | 0.00 | 1.45 | 0.41 | 5.13 | 1.58 | 0.97 | 2.57 | 1.59 | 1.10 | 2.28 | 1.08 | 0.78 | 1.51 | 1.15 | 0.74 | 1.78 |
| Anaemia | 4.04 | 3.75 | 4.35 | 2.39 | 2.19 | 2.60 | 13.09 | 6.96 | 24.64 | 8.04 | 5.50 | 11.75 | 4.34 | 3.48 | 5.42 | 2.67 | 2.29 | 3.10 | 1.99 | 1.74 | 2.28 | 1.67 | 1.42 | 1.95 |
| Raised ALP | 3.43 | 3.16 | 3.73 | 2.72 | 2.50 | 2.95 | 5.43 | 2.75 | 10.71 | 5.51 | 3.75 | 8.09 | 3.11 | 2.46 | 3.92 | 3.29 | 2.84 | 3.81 | 2.47 | 2.13 | 2.85 | 1.86 | 1.55 | 2.23 |
| Raised calcium | 2.78 | 2.07 | 3.75 | 2.65 | 1.96 | 3.56 | 8.50 | 1.95 | 36.96 | 6.12 | 1.95 | 19.17 | 4.10 | 1.89 | 8.90 | 3.36 | 2.06 | 5.48 | 1.98 | 1.08 | 3.63 | 1.41 | 0.67 | 2.98 |
| Raised creatinine | 1.96 | 1.77 | 2.16 | 1.04 | 0.94 | 1.15 | 2.99 | 0.73 | 12.27 | 0.35 | 0.05 | 2.47 | 1.27 | 0.80 | 2.02 | 1.21 | 0.95 | 1.54 | 1.06 | 0.90 | 1.24 | 0.94 | 0.80 | 1.11 |
| Raised ferritin | 1.33 | 1.04 | 1.70 | 1.28 | 1.01 | 1.63 | 5.01 | 0.93 | 27.07 | 2.83 | 1.10 | 7.31 | 1.29 | 0.68 | 2.47 | 1.28 | 0.78 | 2.10 | 1.12 | 0.70 | 1.81 | 1.14 | 0.72 | 1.79 |
| Raised platelets | 3.61 | 3.23 | 4.03 | 3.07 | 2.75 | 3.42 | 6.85 | 2.93 | 16.02 | 7.03 | 4.53 | 10.90 | 5.31 | 4.14 | 6.82 | 3.44 | 2.84 | 4.17 | 2.45 | 2.00 | 3.00 | 1.61 | 1.19 | 2.17 |
| Raised WBC count | 1.99 | 1.80 | 2.21 | 1.80 | 1.62 | 1.98 | 3.58 | 1.73 | 7.41 | 2.59 | 1.66 | 4.05 | 2.65 | 2.08 | 3.37 | 1.81 | 1.50 | 2.19 | 1.52 | 1.26 | 1.84 | 1.47 | 1.18 | 1.83 |
| Low ferritin | 2.37 | 1.90 | 2.96 | 1.69 | 1.35 | 2.12 | 10.91 | 2.04 | 58.35 | 3.00 | 0.89 | 10.10 | 2.82 | 1.55 | 5.14 | 1.68 | 1.02 | 2.78 | 1.29 | 0.85 | 1.96 | 1.60 | 1.11 | 2.33 |
| Low platelets | 1.36 | 1.17 | 1.59 | 1.01 | 0.86 | 1.18 | 0.85 | 0.12 | 6.16 | 2.73 | 1.48 | 5.03 | 1.14 | 0.69 | 1.86 | 1.07 | 0.78 | 1.46 | 0.91 | 0.69 | 1.18 | 0.90 | 0.67 | 1.21 |
| Low WBC count | 0.82 | 0.63 | 1.07 | 0.95 | 0.73 | 1.23 | 0.74 | 0.10 | 5.34 | 0.50 | 0.12 | 2.02 | 0.73 | 0.35 | 1.54 | 0.99 | 0.60 | 1.63 | 0.85 | 0.51 | 1.42 | 1.36 | 0.86 | 2.17 |
| Raised PSA | 3.50 | 3.05 | 4.01 | 2.54 | 2.19 | 2.96 | NA | NA |  | 20.29 | 5.61 | 73.40 | 5.38 | 3.52 | 8.20 | 3.35 | 2.62 | 4.26 | 2.14 | 1.71 | 2.69 | 1.38 | 1.02 | 1.86 |
| FEMALES | | | | | | | | | | | | | | | | | | | | | | | | |
| Low albumin | 3.77 | 3.41 | 4.17 | 2.83 | 2.55 | 3.14 | 2.33 | 1.08 | 5.02 | 3.17 | 1.94 | 5.16 | 3.34 | 2.40 | 4.64 | 3.43 | 2.77 | 4.24 | 2.96 | 2.46 | 3.56 | 2.31 | 1.93 | 2.77 |
| Raised ALT | 1.36 | 1.22 | 1.51 | 1.46 | 1.31 | 1.62 | 1.16 | 0.60 | 2.24 | 1.09 | 0.71 | 1.66 | 1.55 | 1.21 | 1.97 | 1.44 | 1.20 | 1.74 | 1.46 | 1.17 | 1.81 | 1.69 | 1.29 | 2.21 |
| Raised AST | 2.21 | 1.72 | 2.84 | 2.07 | 1.62 | 2.66 | 0.00 | 0.00 | 0.00 | 2.99 | 1.16 | 7.74 | 2.39 | 1.33 | 4.31 | 2.41 | 1.58 | 3.68 | 2.04 | 1.28 | 3.24 | 1.19 | 0.57 | 2.50 |
| Raised bilirubin | 1.58 | 1.35 | 1.84 | 1.55 | 1.33 | 1.80 | 0.24 | 0.03 | 1.75 | 1.80 | 1.07 | 3.03 | 1.86 | 1.24 | 2.79 | 1.57 | 1.16 | 2.12 | 1.37 | 1.02 | 1.84 | 1.73 | 1.29 | 2.34 |
| Raised CRP | 3.49 | 3.12 | 3.90 | 2.90 | 2.59 | 3.24 | 3.53 | 1.99 | 6.26 | 1.78 | 1.23 | 2.57 | 3.03 | 2.31 | 3.97 | 3.86 | 3.11 | 4.79 | 2.62 | 2.11 | 3.26 | 2.50 | 1.89 | 3.30 |
| Raised ESR | 2.53 | 2.24 | 2.85 | 1.85 | 1.63 | 2.09 | 1.77 | 0.91 | 3.42 | 1.69 | 1.15 | 2.49 | 2.21 | 1.62 | 3.01 | 2.10 | 1.67 | 2.65 | 1.67 | 1.32 | 2.11 | 1.51 | 1.12 | 2.04 |
| Raised HbA1c | 2.50 | 2.03 | 3.08 | 1.74 | 1.41 | 2.16 | 10.30 | 1.15 | 92.01 | 1.98 | 0.85 | 4.61 | 1.68 | 0.99 | 2.86 | 1.42 | 0.99 | 2.04 | 2.68 | 1.62 | 4.42 | 1.39 | 0.90 | 2.15 |
| Anaemia | 3.08 | 2.85 | 3.34 | 2.77 | 2.55 | 3.01 | 1.01 | 0.59 | 1.73 | 2.80 | 2.10 | 3.73 | 3.20 | 2.47 | 4.13 | 3.94 | 3.36 | 4.63 | 2.70 | 2.33 | 3.13 | 2.32 | 1.99 | 2.70 |
| Raised ALP | 3.06 | 2.81 | 3.33 | 2.42 | 2.23 | 2.64 | 1.25 | 0.55 | 2.86 | 2.30 | 1.55 | 3.43 | 3.02 | 2.42 | 3.76 | 2.78 | 2.39 | 3.24 | 2.22 | 1.89 | 2.61 | 2.09 | 1.75 | 2.49 |
| Raised calcium | 1.99 | 1.52 | 2.62 | 1.41 | 1.07 | 1.84 | 0.00 | 0.00 | 0.00 | 0.00 | 0.00 | 0.00 | 2.32 | 1.16 | 4.66 | 1.20 | 0.69 | 2.11 | 1.41 | 0.88 | 2.25 | 1.44 | 0.86 | 2.42 |
| Raised creatinine | 2.10 | 1.80 | 2.44 | 1.11 | 0.95 | 1.30 | 0.00 | 0.00 | 0.00 | 3.33 | 1.08 | 10.25 | 1.44 | 0.54 | 3.81 | 1.15 | 0.72 | 1.85 | 1.19 | 0.90 | 1.56 | 1.02 | 0.82 | 1.27 |
| Raised ferritin | 3.32 | 2.52 | 4.37 | 1.98 | 1.50 | 2.61 | 8.25 | 1.13 | 60.30 | 1.59 | 0.22 | 11.27 | 2.45 | 1.09 | 5.51 | 2.38 | 1.42 | 3.98 | 1.66 | 0.96 | 2.88 | 1.76 | 1.07 | 2.88 |
| Raised platelets | 3.91 | 3.60 | 4.25 | 3.39 | 3.12 | 3.68 | 1.73 | 0.91 | 3.30 | 3.54 | 2.62 | 4.77 | 3.77 | 3.00 | 4.73 | 4.18 | 3.59 | 4.86 | 3.21 | 2.74 | 3.75 | 2.78 | 2.33 | 3.33 |
| Raised WBC count | 2.09 | 1.89 | 2.30 | 2.03 | 1.84 | 2.24 | 1.77 | 1.09 | 2.86 | 2.20 | 1.56 | 3.09 | 2.13 | 1.61 | 2.83 | 2.46 | 2.04 | 2.96 | 1.86 | 1.53 | 2.26 | 1.79 | 1.46 | 2.18 |
| Low ferritin | 0.97 | 0.80 | 1.17 | 1.50 | 1.23 | 1.83 | 1.03 | 0.39 | 2.71 | 1.09 | 0.65 | 1.81 | 1.26 | 0.75 | 2.10 | 1.70 | 1.11 | 2.62 | 1.67 | 1.11 | 2.51 | 1.83 | 1.26 | 2.66 |
| Low platelets | 1.44 | 1.16 | 1.78 | 1.22 | 0.98 | 1.51 | 2.16 | 0.89 | 5.25 | 2.06 | 1.03 | 4.14 | 0.74 | 0.31 | 1.79 | 1.61 | 1.08 | 2.41 | 1.34 | 0.92 | 1.94 | 0.70 | 0.42 | 1.18 |
| Low WBC count | 0.62 | 0.49 | 0.79 | 0.64 | 0.51 | 0.82 | 0.77 | 0.25 | 2.42 | 0.97 | 0.50 | 1.88 | 0.69 | 0.40 | 1.19 | 0.48 | 0.29 | 0.80 | 0.79 | 0.50 | 1.25 | 0.48 | 0.24 | 0.96 |
| Raised CA125 | 13.80 | 11.50 | 16.56 | 14.87 | 12.38 | 17.85 | 11.81 | 3.47 | 40.23 | 15.66 | 9.02 | 27.17 | 18.25 | 12.16 | 27.39 | 18.94 | 13.82 | 25.96 | 13.97 | 9.87 | 19.76 | 6.38 | 3.83 | 10.64 |

##### Table N: Risk ratios (RR) comparing 1 year cancer risk in patient with abdominal bloating and a normal versus abnormal test result for 19 different blood test abnormalities. *adjusted for age

|  | Crude RR | | | Adjusted RR* | | | Age-stratified RR  (30-39 years) | | | Age-stratified RR  (40-49 years) | | | Age-stratified RR  (50-59 years) | | | Age-stratified RR  (60-69 years) | | | Age-stratified RR  (70-79 years) | | | Age-stratified RR  (≥80 years) | | |
| --- | --- | --- | --- | --- | --- | --- | --- | --- | --- | --- | --- | --- | --- | --- | --- | --- | --- | --- | --- | --- | --- | --- | --- | --- |
| Blood test abnormality | **RR** | **95%CI** | | **RR** | **95% CI** | | **RR** | **95% CI** | | **RR** | **95% CI** | | **RR** | **95% CI** | | **RR** | **95% CI** | | **RR** | **95% CI** | | **RR** | **95% CI** | |
| MALES | | | | | | | | | | | | | | | | | | | | | | | | |
| Low albumin | 4.09 | 2.97 | 5.63 | 2.83 | 2.03 | 3.95 | 0.00 | 0.00 | 0.00 | 5.93 | 0.78 | 45.29 | 4.92 | 1.97 | 12.25 | 4.58 | 2.55 | 8.25 | 2.17 | 1.21 | 3.90 | 1.97 | 1.05 | 3.71 |
| Raised ALT | 0.52 | 0.36 | 0.76 | 0.83 | 0.56 | 1.23 | 0.70 | 0.07 | 6.67 | 1.03 | 0.25 | 4.31 | 0.51 | 0.19 | 1.33 | 0.66 | 0.31 | 1.38 | 1.03 | 0.53 | 2.01 | 1.66 | 0.69 | 4.01 |
| Raised AST | 2.44 | 1.26 | 4.71 | 2.86 | 1.51 | 5.43 | 0.00 | 0.00 | 0.00 | 6.29 | 0.40 | 98.74 | 2.46 | 0.46 | 13.06 | 2.23 | 0.51 | 9.84 | 2.25 | 0.68 | 7.41 | 5.20 | 1.75 | 15.42 |
| Raised bilirubin | 1.65 | 1.15 | 2.37 | 1.54 | 1.07 | 2.20 | 0.00 | 0.00 | 0.00 | 5.03 | 1.32 | 19.22 | 1.26 | 0.39 | 4.06 | 1.81 | 0.91 | 3.58 | 1.48 | 0.83 | 2.64 | 1.15 | 0.48 | 2.79 |
| Raised CRP | 4.95 | 3.25 | 7.55 | 3.60 | 2.33 | 5.57 | 0.00 | 0.00 | 0.00 | 6.77 | 0.62 | 74.32 | 5.54 | 1.92 | 15.99 | 2.55 | 1.16 | 5.59 | 4.76 | 2.11 | 10.75 | 1.83 | 0.75 | 4.45 |
| Raised ESR | 3.78 | 2.48 | 5.74 | 2.49 | 1.57 | 3.95 | 6.90 | 0.44 | 109.01 | 6.86 | 1.16 | 40.68 | 6.78 | 2.11 | 21.73 | 2.50 | 1.03 | 6.04 | 1.80 | 0.91 | 3.55 | 1.22 | 0.47 | 3.16 |
| Raised HbA1c | 1.90 | 0.98 | 3.66 | 1.34 | 0.67 | 2.69 | NA | NA | NA | 0.00 | 0.00 | 0.00 | 0.00 | 0.00 | 0.00 | 0.85 | 0.23 | 3.17 | 0.81 | 0.31 | 2.13 | 1.10 | 0.30 | 3.96 |
| Anaemia | 3.22 | 2.48 | 4.17 | 1.98 | 1.47 | 2.66 | 0.00 | 0.00 | 0.00 | 9.92 | 3.13 | 31.40 | 3.48 | 1.64 | 7.40 | 2.74 | 1.57 | 4.79 | 1.55 | 1.01 | 2.39 | 1.34 | 0.77 | 2.36 |
| Raised ALP | 2.73 | 2.03 | 3.69 | 2.24 | 1.66 | 3.02 | 4.48 | 0.51 | 39.25 | 6.31 | 1.66 | 24.05 | 3.13 | 1.38 | 7.07 | 2.40 | 1.31 | 4.41 | 2.06 | 1.27 | 3.33 | 1.51 | 0.76 | 3.00 |
| Raised calcium | 4.12 | 1.79 | 9.50 | 4.33 | 1.92 | 9.74 | 0.00 | 0.00 | 0.00 | 0.00 | 0.00 | 0.00 | 7.77 | 1.16 | 52.01 | 5.86 | 2.14 | 16.02 | 0.00 | 0.00 | 0.00 | 3.39 | 0.58 | 19.82 |
| Raised creatinine | 1.66 | 1.18 | 2.35 | 0.94 | 0.65 | 1.35 | 0.00 | 0.00 | 0.00 | 3.09 | 0.41 | 23.45 | 2.54 | 0.80 | 8.04 | 0.22 | 0.03 | 1.54 | 1.22 | 0.75 | 1.99 | 0.70 | 0.37 | 1.32 |
| Raised ferritin | 0.90 | 0.28 | 2.95 | 0.88 | 0.27 | 2.86 | NA | NA | NA | 3.27 | 0.30 | 35.16 | 0.00 | 0.00 | 0.00 | 0.93 | 0.11 | 7.58 | 0.94 | 0.12 | 7.23 | 0.00 | 0.00 | 0.00 |
| Raised platelets | 3.81 | 2.50 | 5.80 | 3.23 | 2.12 | 4.93 | 0.00 | 0.00 | 0.00 | 8.65 | 2.00 | 37.48 | 4.17 | 1.35 | 12.87 | 4.54 | 2.19 | 9.41 | 1.75 | 0.69 | 4.45 | 3.00 | 1.30 | 6.92 |
| Raised WBC count | 2.51 | 1.77 | 3.55 | 2.16 | 1.53 | 3.05 | 5.59 | 0.64 | 48.89 | 1.57 | 0.21 | 11.97 | 2.88 | 1.16 | 7.16 | 2.20 | 1.08 | 4.49 | 2.46 | 1.48 | 4.09 | 1.15 | 0.43 | 3.06 |
| Low ferritin | 1.78 | 0.69 | 4.57 | 1.31 | 0.52 | 3.33 | NA | NA | NA | 0.00 | 0.00 | 0.00 | 0.00 | 0.00 | 0.00 | 1.53 | 0.19 | 12.23 | 1.25 | 0.27 | 5.78 | 2.44 | 0.48 | 12.38 |
| Low platelets | 1.28 | 0.80 | 2.05 | 0.94 | 0.58 | 1.51 | 0.00 | 0.00 | 0.00 | 4.87 | 1.10 | 21.45 | 0.58 | 0.08 | 4.18 | 1.42 | 0.58 | 3.47 | 1.08 | 0.56 | 2.09 | 0.19 | 0.03 | 1.36 |
| Low WBC count | 0.72 | 0.30 | 1.73 | 0.76 | 0.32 | 1.84 | 0.00 | 0.00 | 0.00 | 0.00 | 0.00 | 0.00 | 2.41 | 0.60 | 9.63 | 1.43 | 0.36 | 5.63 | 0.40 | 0.06 | 2.81 | 0.00 | 0.00 | 0.00 |
| Raised PSA | 2.63 | 1.68 | 4.12 | 1.68 | 1.02 | 2.76 | NA | NA | NA | 0.00 | 0.00 | 0.00 | 18.08 | 1.69 | 193.49 | 3.69 | 1.49 | 9.11 | 0.95 | 0.47 | 1.93 | 1.40 | 0.57 | 3.40 |
| FEMALES | | | | | | | | | | | | | | | | | | | | | | | | |
| Low albumin | 5.61 | 4.47 | 7.03 | 4.20 | 3.33 | 5.30 | 4.60 | 0.61 | 34.75 | 5.35 | 1.96 | 14.59 | 5.71 | 3.10 | 10.53 | 4.76 | 3.10 | 7.29 | 3.62 | 2.36 | 5.54 | 3.55 | 2.23 | 5.66 |
| Raised ALT | 1.43 | 1.07 | 1.90 | 1.46 | 1.09 | 1.95 | 0.00 | 0.00 | 0.00 | 2.10 | 0.89 | 4.98 | 1.03 | 0.50 | 2.14 | 1.35 | 0.84 | 2.16 | 1.75 | 0.99 | 3.12 | 2.08 | 0.94 | 4.61 |
| Raised AST | 1.51 | 0.66 | 3.44 | 1.55 | 0.69 | 3.50 | 0.00 | 0.00 | 0.00 | 0.00 | 0.00 | 0.00 | 0.00 | 0.00 | 0.00 | 2.30 | 0.51 | 10.36 | 0.95 | 0.13 | 6.73 | 7.57 | 2.61 | 21.98 |
| Raised bilirubin | 1.16 | 0.72 | 1.88 | 1.19 | 0.74 | 1.92 | 0.00 | 0.00 | 0.00 | 2.26 | 0.71 | 7.19 | 0.96 | 0.24 | 3.86 | 1.13 | 0.43 | 2.99 | 0.95 | 0.36 | 2.52 | 1.55 | 0.59 | 4.08 |
| Raised CRP | 4.97 | 3.75 | 6.59 | 4.06 | 3.05 | 5.39 | 7.71 | 0.70 | 84.80 | 3.14 | 1.24 | 7.90 | 7.65 | 3.74 | 15.64 | 3.44 | 2.16 | 5.48 | 3.32 | 1.86 | 5.92 | 4.23 | 1.82 | 9.79 |
| Raised ESR | 3.93 | 2.87 | 5.39 | 3.01 | 2.20 | 4.12 | 2.16 | 0.54 | 8.62 | 1.88 | 0.75 | 4.72 | 3.31 | 1.56 | 7.03 | 2.66 | 1.55 | 4.58 | 5.22 | 2.24 | 12.19 | 3.03 | 1.18 | 7.77 |
| Raised HbA1c | 2.07 | 1.32 | 3.26 | 1.47 | 0.93 | 2.32 | 3.26 | 0.21 | 51.72 | 1.36 | 0.31 | 6.04 | 0.88 | 0.24 | 3.27 | 1.21 | 0.49 | 3.00 | 1.32 | 0.62 | 2.80 | 6.10 | 0.81 | 45.67 |
| Anaemia | 2.82 | 2.26 | 3.53 | 2.45 | 1.96 | 3.07 | 0.00 | 0.00 | 0.00 | 2.51 | 1.19 | 5.30 | 2.35 | 1.19 | 4.64 | 2.26 | 1.38 | 3.72 | 2.35 | 1.50 | 3.69 | 3.01 | 1.99 | 4.54 |
| Raised ALP | 2.80 | 2.22 | 3.53 | 2.22 | 1.76 | 2.79 | 5.32 | 1.19 | 23.66 | 1.00 | 0.24 | 4.12 | 2.93 | 1.65 | 5.19 | 3.06 | 2.09 | 4.49 | 1.48 | 0.92 | 2.39 | 1.95 | 1.15 | 3.32 |
| Raised calcium | 1.20 | 0.50 | 2.88 | 0.84 | 0.35 | 2.01 | 0.00 | 0.00 | 0.00 | 0.00 | 0.00 | 0.00 | 1.72 | 0.24 | 12.33 | 0.74 | 0.11 | 5.17 | 0.51 | 0.07 | 3.58 | 1.06 | 0.27 | 4.24 |
| Raised creatinine | 2.18 | 1.41 | 3.36 | 1.18 | 0.75 | 1.86 | 0.00 | 0.00 | 0.00 | 0.00 | 0.00 | 0.00 | 7.27 | 1.95 | 27.06 | 1.03 | 0.26 | 4.05 | 1.56 | 0.75 | 3.24 | 0.87 | 0.44 | 1.70 |
| Raised ferritin | 3.87 | 1.74 | 8.63 | 2.69 | 1.21 | 6.01 | 0.00 | 0.00 | 0.00 | 0.00 | 0.00 | 0.00 | 5.30 | 1.31 | 21.42 | 1.51 | 0.21 | 10.57 | 4.27 | 1.06 | 17.25 | 1.59 | 0.23 | 11.00 |
| Raised platelets | 5.43 | 4.43 | 6.65 | 4.71 | 3.85 | 5.77 | 2.11 | 0.28 | 15.81 | 7.46 | 4.02 | 13.84 | 5.86 | 3.58 | 9.61 | 4.73 | 3.29 | 6.79 | 4.84 | 3.27 | 7.16 | 3.04 | 1.82 | 5.07 |
| Raised WBC count | 2.80 | 2.16 | 3.63 | 2.60 | 2.00 | 3.37 | 4.95 | 1.42 | 17.21 | 3.00 | 1.29 | 6.95 | 3.24 | 1.75 | 6.02 | 2.57 | 1.57 | 4.22 | 2.94 | 1.79 | 4.84 | 1.50 | 0.77 | 2.92 |
| Low ferritin | 0.80 | 0.47 | 1.38 | 1.29 | 0.76 | 2.19 | 4.50 | 0.41 | 49.44 | 2.92 | 0.66 | 13.02 | 0.92 | 0.27 | 3.10 | 0.34 | 0.05 | 2.51 | 2.69 | 0.80 | 9.06 | 1.12 | 0.33 | 3.73 |
| Low platelets | 1.21 | 0.69 | 2.14 | 1.02 | 0.58 | 1.79 | 3.54 | 0.47 | 26.46 | 1.47 | 0.21 | 10.49 | 0.68 | 0.10 | 4.85 | 0.64 | 0.16 | 2.52 | 1.49 | 0.63 | 3.55 | 0.70 | 0.18 | 2.77 |
| Low WBC count | 0.38 | 0.18 | 0.80 | 0.39 | 0.19 | 0.82 | 0.00 | 0.00 | 0.00 | 0.57 | 0.08 | 4.11 | 0.60 | 0.15 | 2.41 | 0.16 | 0.02 | 1.15 | 0.30 | 0.04 | 2.09 | 0.76 | 0.19 | 3.01 |
| Raised CA125 | 19.74 | 15.03 | 25.91 | 19.78 | 15.15 | 25.82 | 11.03 | 2.74 | 44.41 | 14.33 | 5.92 | 34.69 | 18.55 | 10.44 | 32.95 | 23.86 | 15.21 | 37.42 | 29.88 | 15.56 | 57.37 | 11.23 | 5.14 | 24.53 |

# Study analysis plan

*Study period for identification of symptomatic cohort:*

- 1/1/2006 – 31/10/2016
- As 12 months of data is needed pre-presentation date to ensure the symptomatic presentation (abdominal pain/bloating) is a new event, all symptom dates will be from 1/1/2007 – 31/10/2016

*Individual patient eligible study period:*

- Eligibility start date, the latest of: CPRD case registration date (CRD)+365 days, CPRD up to standard date (UTS), year of birth + 30*365.25 and 01/01/2007
- Eligibility end date, the earliest of: LCD, TOD, date of death and 31/10/2016

*Defining the symptomatic cohort:*

1. Extract all patients who during the eligible study period had a record in CPRD of abdominal pain or bloating
2. Flag all valid symptom events (i.e. no previous record of abdominal pain/bloating in the 365 days prior to the event) and keep patients with at least one valid symptom event during their eligible study period.
3. Select the first valid symptom event and allocate the date of this symptom as the symptom date
4. Exclusions: Drop any patients who:

- do not have a valid symptom during follow up
- have a diagnosis of any cancer before the symptom date
- have <1day of follow up
- Do not have a male/female gender recorded or year of birth recorded
- Are not eligible for linkage to the NCRAS cancer registry database

*Defining the exposure:*

1. A first cohort will be made of all patients with abdominal pain/bloating who had a blood test in the three months **before the index symptom presentation**.
2. Data will be extracted on all blood test use and findings during this window
3. Blood tests for inclusion will be selected *a priori* informed by clinical knowledge and previous work in this area (Nicholson et al, Watson et al.) and will include:
   - Platelets, albumin, ESR, CRP, WCC, Ferritin, hb, creatinine, bilirubin, AST, ALT, ALP, Calcium, HbA1c, PSA, CA125
4. Blood tests will be categorised as ‘normal’ or ‘abnormal’ based on standard laboratory reference ranges. For instances where there are multiple blood tests in the window the presence of any abnormal test will be recorded.
5. A second cohort will be made of all patients with a blood test who had an abdominal pain/bloating presentation in the three months **before the blood test**.
6. For eligible episodes the blood tests will be categorised as ‘normal’ or ‘abnormal’ using the method outlined above.
7. The two cohorts will be merged and the first eligible index date will be selected (either symptomatic presentation for cohort 1 or blood test for cohort 2)
8. Patients will be followed up for 12 months following the index date to see if they develop cancer of any site
9. PPV calculations will be done for each blood test abnormality in a symptomatic cohort of patients with abdominal pain or bloating (by age and sex).

*Defining the outcome:*

- Any new cancer diagnosis recorded in NCRAS in the 12 months following the index date will be flagged and the corresponding earliest diagnosis date and site will be recorded.
- Cumulative incidence curves of cancer incidence(or death) over time will be plotted starting from the symptom presentation to establish when the increased incidence plateaus and confirm 12 month follow up is appropriate.

*Co-variates:*

1. Variables will be created for the following demographics: year of birth, age at symptom date, sex and socioeconomic status (using IMD quintiles)

*Statistical analysis plan:*

Analyses will be stratified by sex and 10-year age bands and conducted separately for patients with abdominal pain vs abdominal bloating

In patients with abdominal pain/bloating, for the following scenarios diagnostic accuracy statistics (Positive and negative likelihood ratios, PPVs and NPVs with 95% confidence intervals) will be calculated for cancer in the 12 months following index date using 2x2 tables and the DIAGT Stata module:

- Abdominal pain / bloating symptom presentation
- Having a blood test request around symptom presentation
- Having an abnormal blood test result considering all blood tests combined (i.e. any abnormal test vs none)
- Having an individual abnormal blood test results (for each of the blood tests)
