# Supplementary material for: Predictive value of abnormal blood tests for detecting cancer in primary care patients with nonspecific abdominal symptoms: A population-based cohort study of 477,870 patients in England
Source: PLoS Med. 2024 Jul 30;21(7):e1004426. doi: 10.1371/journal.pmed.1004426 (PMC11288431; doi:10.1371/journal.pmed.1004426)
Supplement: Attachment — Submitted filename: Response to reviewers.docx [file pmed.1004426.s003.docx]

**Authors’ reply to Reviewer Comments, Manuscript PMEDICINE-D-24-00134R1, by Rafiq et al., “Predictive value of abnormal blood tests for detecting cancer in primary care patients with non-specific abdominal symptoms: A population-based cohort study of 477,870 patients in England”.**

Dear Dr Dodd,

Thank you very much for the constructive reviews of our paper. We have addressed each Editor and Reviewer comment below, and in addition summarised the three main themes arising from their reviews:

1.) Describing/explaining the aspects of the study design relating to handling immortal time bias. As appraised positively by the Academic Editor, our study was carefully designed to minimise immortal time bias. Yet we were inconsistent in the manuscript in describing this feature of the study design, which we have now addressed.
2.) Clarifying the intended translational pathway and clinical implications. We now explain our view that the clinical translation of the findings will be through enabling more informed use of the information arising from blood test results that GPs already order, and not through increasing blood test use, nor through requesting each of the examined blood tests in patients presenting with these symptoms. Until now, there has been no evidence explicitly quantifying the levels of risk associated with positive or negative test results, and our study addresses this evidence gap. These patients have already been selected by their GP to undergo the blood tests, but our findings can help to more accurately identify patients at highest risk of having underlying cancer. Prior research from our group indicates that GPs frequently use blood tests, with 62% and 64% of patients with cancer who initially presented with abdominal pain or abdominal bloating receiving common blood tests, respectively (Cranfield et al., Cancers, 2023). The Discussion section on implications of our findings has also been expanded, and includes: updating of current referral guidelines to encompass blood test results in practice recommendations; ranking of cancer sites by level or risk to guide the choice of onward investigations and choice of referral specialty in patients with abnormal blood test results; and consideration of healthcare system consequences that may result from these recommendations (increased referral numbers and testing, which may support future health economic analyses).
3.) The value of information from a blood test vs a multivariable predictive model. The study findings demonstrate that in patients in certain age groups who present with abdominal pain and bloating and are tested by a common blood test, the test result can help to classify them into categories above/below the 3% NICE threshold for recommending urgent cancer referral. We have elaborated on the clinical implications of this in the discussion and also explore potential future expansions of the findings, including combining multiple results/features in risk prediction models +/- integration into existing general practice IT systems, which could further support their implementation and improve cancer risk assessment in patients presenting with these symptoms.

These additional clarifications have now been added to the manuscript as suggested by the Reviewers. For ease of reading any altered text from the manuscript has been included in the point-to-point response to Reviewers’ with the new text highlighted in yellow.

| **Editorial comments** | **Response** |
| --- | --- |
| **E1-1:** We enjoyed reading your paper and agree that it offers some valuable insights.  However, we also agree with the reviewers that the lack of methodological detail included make it difficult to understand and that further consideration should be given to the relevance in clinical practice and implications for healthcare systems. Please revise your manuscript in light of reviewer and Academic Editor comments detailed below. | We thank the Editor for their positive assessment of our paper. In response to the Reviewer and Academic Editor comments, we have now added additional details to the methods including:   - Clarification of the study design to avoid immortal time bias - Providing further details on the modelling of numbers of referred patients - Calculation of Risk Ratios to assist the portability of the findings - Including codelists for the included blood tests in the Supplementary File and providing details of the methods for developing and validating the symptom codelists. - Information on patient follow up.   Additionally, we have also provided more details on the relevance to clinical practice and potential implications for healthcare systems, specifically:   - How the evidence can inform updates to national cancer referral guidelines to include results from these blood tests (without recommending increased test use). - How the evidence presented on ranking of cancer sites by highest risk can be used by clinicians to guide onward investigation strategies / referrals in symptomatic patients with these abnormal blood test results. - How abnormal blood test results can increase clinician confidence in the need for further cancer assessment in patients with abdominal pain and bloating, and thereby increase guideline concordance. - Consideration of potential healthcare system consequences that may result from these recommendations relating to costs, referral numbers and increased testing in primary and secondary care. |
| **E1-2:** Did your study have a prospective protocol or analysis plan? Please state this (either way) early in the Methods section. a) If a prospective analysis plan (from your funding proposal, IRB or other ethics committee submission, study protocol, or other planning document written before analyzing the data) was used in designing the study, please include the relevant prospectively written document with your revised manuscript as a Supporting Information file to be published alongside your study, and cite it in the Methods section. A legend for this file should be included at the end of your manuscript.  b) If no such document exists, please make sure that the Methods section transparently describes when analyses were planned, and when/why any data-driven changes to analyses took place.  c) In either case, changes in the analysis-- including those made in response to peer review comments-- should be identified as such in the Methods section of the paper, with rationale. | Our study relates to a protocol aimed at examining the positive predictive value for cancer of different presenting features, such as symptoms combined with common blood test results. We further specified this protocol into an analysis plan and have added this as a Supporting Information file as requested. Additionally, we now clarify in the methods any changes / additions to the analysis. This has been added to the manuscript as follows:  Methods (page 5 line 22): *(the analysis plan can be found in the Supplementary file).*  Methods (page 8 line 25): *“*Negative predictive value (NPV), sensitivity, specificity and positive and negative likelihood ratios *were also estimated, alongside risk ratios comparing cancer risk in patients with a normal versus abnormal test result using Poisson regression (risk ratios component was a post hoc analysis).* *Cancer-site specific PPVs were then calculated for each blood test (post hoc analysis) and used to rank cancer sites in order of likelihood to guide subsequent investigation / referral strategies. To estimate the potential impact of incorporating information from blood test results into cancer referral recommendations, the baseline number of patients where an urgent referral would be recommended based on their presenting symptom, age and sex was modelled and compared to a model additionally using information from blood test results (post hoc analysis).”* |
| **Academic Editor (AE) comments** | **Response** |
| **AE-1:** This is an intriguing, methodologically well-done analysis that could potentially influence clinical practice. It is also an excellent example of how integrated medical practices with large datasets can use such data to better serve their clientele. | Thank you for this positive assessment of our paper and its methodological quality, and your reflections on how the findings could impact clinical practice. |
| **AE-2A:** I have just a few thoughts to offer:  First, I greatly appreciate the care that the authors put into correctly determining how to count person-time and avoid immortality bias. The authors note two events that must occur before follow-up can start: 1) a blood test and 2) a symptom report of abdominal pain. The date by which both events have occurred is the index date for follow up. Logically speaking, cancer diagnoses between events should not be counted, else results could be affected by “immortality bias”.  While they appear to correctly handle this issue, there are inconsistencies in their descriptions of the analysis. For example, on page 6, the manuscript states that each patient was followed up for 12 months after their first symptom date (line 2), which is not a technically accurate description (they were followed 12 months after the index date). On page 7 (line 20), it states that the PPV was calculated for cancer diagnosis after the first symptom date (again, it should read as index date). | Thank you for the apt summary and positive assessment of the method we applied to avoid immortal time bias.  The inconsistency in the use of first symptom date vs index date arises as the study has two components:   1.) Calculation of symptom PPVs: The PPV of new abdominal pain or bloating for cancer in the following year is estimated. In this scenario there is no issue of immortal time bias as the symptom event is being considered in isolation and cancer diagnosis is measured in the 1 year after the ‘first symptom date’. 2.) Calculation of symptom + blood test PPVs: This is the scenario described by the Editor, where blood tests occurring around the time of symptom presentation are taken into consideration and the date by which both events have occurred is the index date (to avoid immortal time bias).   We have now added a paragraph to clearly describe these two stages and clarify the description of the study design to ensure consistency throughout the manuscript as follows:  Methods (page 5 line 24) *“Study population: This study involved two different study populations. Firstly, a population of patients who present in primary care with new abdominal pain and bloating (the symptomatic population); this population was used to estimate the predictive value of each symptom (considered in isolation) for detecting underlying cancer in the following year. Secondly, a sub-group of the above population, comprising the symptomatic patients (as above) who also had blood tests around the time of their presentation with the studied symptoms (the tested symptomatic population); this population was used to the estimate the predictive value of symptoms + blood tests for cancer in the following year. The two study populations were identified as follows.”*  Methods (page 6 line 31) *“Only patients who had primary care blood tests occurring around the time of symptomatic presentation were included in the ‘tested’ symptomatic population.”*  Methods (page 7 line 15) *“Each patient was followed up for 12 months after their ~~first symptom~~ index date to identify any new cancer diagnoses”* |
| **AE-2B:** Additionally, reporting the results for two separate groups (those who had the blood test first and those who the symptom first) seems unnecessary to me and has the potential to confuse the reader. The use of the index date is just the appropriate way to count person time. If the authors think that a sensitivity analysis separating these groups is necessary, that is fine. But it should be described briefly as a sensitivity analysis.  I must also add that it is possible that I have misread how analyses were done, given these apparent inconsistencies. If so, that is a more serious problem, indicating a true lack of clarity in their descriptions. In this scenario, the authors would need to take strong steps to clarify the statistical analysis. | We feel it is useful to report results for both the ‘test then symptom’ and the ‘symptom then test’ subgroups, and find it both interesting and important to observe that the two subgroups had a similar sample size, and that cancer risk was of similar order of magnitude. However, we agree there is a risk of confusion and following the Academic Editor’s suggestion this has now been specified as a sensitivity analysis and included in the Supplementary Tables S8 and S9. We have now added these details in the methods and results as follows:  Methods (page 9 line 9): *“Sensitivity analyses were conducted using chi-squared tests to compare patient demographics and 1 year cancer incidence in patients identified from the two scenarios (blood test followed by symptom vs symptom followed by blood test) to ensure the populations and outcomes were similar.”*  Results (page 13 line 11): “Sensitivity analysis comparing tested symptomatic patients identified from the two scenarios (blood test before symptom vs blood test after symptom) showed that the populations were similar in size, broadly comparable with regards to demographics and had similar 1 year cancer risk (Supplementary Tables S8 and S9).” |
| **AE-3:** Second, the authors should consider reporting hazard ratios (or risk ratios) for the different blood test results, at least in a supplement. Hazard ratios tend to be more transportable from cohort to cohort as they depend far less on the prevalence of the exposure. Reporting hazard ratios would facilitate comparisons across studies. I do not see it as necessary, but it would be helpful. | We thank the Editor for this useful suggestion, which we agree will increase the portability of the study findings. In diagnostic research (such as this study where we are looking for whether there is an undetected cancer present rather than the future risk of developing cancer) the hazards are often strongly non-proportional (being particularly strong soon after the presentation and decreasing in size thereafter) and so hazard ratios are typically of limited use. For this reason, following what we believe is consistent with the spirit of the Editor’s suggestion we have now calculated and include risk ratios for each blood test result by age and sex instead. This has been included as supplementary tables 12a and 12b and added to the manuscript as follows:  Methods (page 8 line 25): *“Negative predictive value (NPV), sensitivity, specificity and positive and negative likelihood ratios were also estimated, alongside risk ratios comparing cancer risk in patients with a normal versus abnormal test result using Poisson regression (the risk ratios component was a post hoc analysis).”*  Results (page 14 line 6): *“Risk ratios comparing 1 year cancer risk in symptomatic patients with a normal versus abnormal blood test result are shown in Supplementary Table S12 for each of the 19 blood test abnormalities examined.”* |
| **AE-4:** Third, the screening method proposed here would likely entail a great expansion in cancer testing regimes. The authors should provide some sense of how expanded and what burden this might place on the medical system. | This is an important issue. We have now clarified the clinical implications in the text, emphasising that we are not proposing an increase in blood test use, but rather better utilisation of data from existing blood test results that have already been requested by GPs. This is an important clarification as we know that patients who are selected for blood testing in primary care have a higher cancer risk that untested patients, and if all symptomatic patients were to undergo ‘screening’ blood tests in primary care the PPVs of abnormal results in practice will be lower (1) as the background risk of cancer in (presently / historically) not tested patients is lower than that in tested patients. This has been expanded on in the manuscript as follows:  Discussion (page 20 line 14): *“Incorporating these blood tests into referral recommendations could help to better utilise data from existing blood test results and detect additional patients with underlying cancer via expedited routes.”*  Discussion (page 21 line 25): *“a key limitation is that the predictive value of blood tests abnormalities presented in this study relates to patients who have already been selected by their general practitioner to have blood tests. The findings of this and previous studies have shown that patients selected for testing are more likely to have cancer than untested patients (2-6)…. The estimates presented in this study therefore apply to patients who are already selected for blood testing in primary care and will be lower in the broader population of all patients presenting with abdominal pain or bloating. We are therefore not recommending an increase in the use of blood tests in primary care, but rather better use of existing results from blood tests in the patient population selected for testing.”*  Discussion (page 24 line 5): *“Our findings show that existing results from readily available primary care blood tests could be better used to improve triaging and risk stratification of patients with abdominal pain and bloating in primary care, especially patients aged 30-59 where there is uncertainty regarding onward management.”*  Discussion (page 24 line 12): *“As primary care clinicians are already selecting patients with abdominal pain and bloating who are higher risk of serious disease for testing (1) and around two thirds cancer patients with these symptoms are known to have routine blood tests done in primary care (7), we are not proposing an increase in blood test use. Rather, the findings present an efficient and affordable way to identify those at highest risk of underlying cancer using information from existing blood tests.”*  Related to this, blood test use in primary care is changing over time and progressively increasing which could impact the PPVs in practice. Periodic re-estimation of PPVs will therefore be needed to take into account changes in blood test usage over time. This has been included in the manuscript as follows:  Discussion (page 22 line 11): *“caution is needed when translating our findings to other settings or timeframes with different rates of primary care blood tests, and periodic re-estimation of the PPVs from this study will be needed to take into account possible changes in blood test use over time.”*  It is possible that an increase in blood testing may occur as an unintended consequence of including the findings of this study in clinical guidelines. This would place an increased burden on the medical system both in terms of primary care testing and follow up appointments. There will also be an anticipated additional burden on the secondary care system due to increased specialist referrals for patients with a cancer risk >3% (modelled in this study) to detect a small number of additional cancers. These healthcare system impacts have now been considered in the manuscript as follows:  Discussion (page 22 line 6): *“Increased testing may also occur as a potential unintended consequence of increased inclusion of blood test results in national cancer guidance. If testing rates are higher and more patients are tested, the pre-test risk of cancer in these less selective tested populations will be lower (if cancer incidence remains stable). The predictive value of blood test abnormalities for cancer in these lower risk populations will therefore be reduced (1).”*  Discussion (page 25 line 14): “*The study findings have potential health system implications. Inclusion of blood test results in cancer referral clinical guidelines could have the unintended consequence of increasing primary care blood test requests, which would place an additional burden on phlebotomy and primary care services. Additionally, as modelled in this study, the expected increase in cancer patients detected through urgent referral pathways will come at the cost of increased secondary care referrals and specialist investigations. Consistent with most available evidence in our field and NICE guideline recommendations that do not incorporate consideration of stage at diagnosis, we have not stratified by stage at diagnosis. However, as cancers diagnosed through urgent referral routes have substantially higher 1-year survival (8) and diagnostic intervals are shorter for patients on urgent pathways (9) (10), we can posit that if the implementation of our study findings result in a greater number of cancers diagnosed through such referrals, this will also result in more cancers being diagnosed at an earlier stage improving prognosis. Formal modelling of cancer stage is however needed to confirm this alongside health economic analysis to evaluate health system effects and ensure appropriate infrastructure and resources are in place*.” |
| **AE-5:** Fourth, the numbers for abdominal bloating are sometimes small and the results variable (Tables 3b). Could this be collapsed with abdominal pain? Why or why not? | In both men and women, abdominal pain and abdominal bloating are distinct clinical entities that do not typically co-occur and often have different aetiologies, differentials and management options. Accordingly, the PPVs reported in this study for abnormal blood tests differ between these two symptoms, for example a 40 year old female with abdominal pain and raised platelets has a 2.6% risk of cancer which is below the referral threshold, whereas the same female patient with abdominal bloating and raised platelets has a 4.9% cancer risk and referral would be recommended. Furthermore, when cancer is diagnosed, the composition of the sample of cancers of different organs (the ‘cancer signature’) of either symptom is quite distinct, particularly in women, where abdominal bloating shows an affinity with ovarian cancer, in a way that is not encountered for abdominal pain. We appreciate this decision comes at the cost of a smaller sample size for some of the less common blood tests. This issue has now been discussed in the manuscript as follows:  Discussion (page 21 line 10): *“As abdominal bloating is less common than abdominal pain, for some blood tests the number of abdominal bloating patients tested is small. These two symptoms have different aetiologies, management and cancer site risk profiles (11) and were therefore considered separately.”* |
| **AE-6:** Fifth, with respect to the discussion, is it not possible for these calculations to be automated in a large health care system? I think this bears mentioning in the discussion, even if just for a sentence or two. Maybe the lesson here is not that we should conduct additional tests but rather better use the data we already have to make better referrals. | We completely agree with the Editor that automating the findings of this study by integrating them into existing GP IT systems would support their implementation into clinical practice and assist GPs to better use the data from existing results (see also response to comment **AE-4** above). This suggestion has now been added to the discussion as follows:  Discussion (page 26 line 1): *“It may therefore be challenging for clinicians to identify relevant abnormalities in practice unless supported by automated clinical decision support tools integrated into existing IT systems to help process and interpret the wealth of information in blood test data (12).”*  We also strongly agree with the Academic Editors point: “*Maybe the lesson here is not that we should conduct additional tests but rather better use the data we already have to make better referrals*.” Indeed, this is the key translational aspect of the findings and we have clarified this as per reply to Academic Editors’ comment **AE-4**. |
| **AE-7:** Lastly, I greatly appreciated the authors’ careful description of their study’s limitations. | We appreciate the Academic Editor’s useful comments and positive assessment of the paper. |

| **Reviewer 1 comments** | **Response** |
| --- | --- |
| **R1-1:** TITLE & ABSTRACT The abstract should specify all types of data used - please make reference to the HES APC and IMD databases here. | The abstract has now been updated to specify all datasets used as follows:  *“Using data from the UK Clinical Practice Research Datalink linked to the National Cancer Registry, Hospital Episode Statistics and Index of Multiple Deprivation”* |
| **R1-2:** Were there any prespecified hypotheses? If so, these should be stated in the objectives. | While it is conventional to consider such hypotheses, contemporary methodologists argue against such statements. Instead, they advocate that specifying the targeted effect or association that the study aims to quantify is the preferred approach and avoids introducing bias in interpretation of the study findings. They suggest that research ought to be: “ *...evaluated by .... relevance and methodological quality rather than by qualitative assertions about reality before the study is conducted.*” See: Hernán MA, Greenland S. Why Stating Hypotheses in Grant Applications Is Unnecessary. JAMA. 2024 ;331(4):285-286. Consequently, we have not altered the objectives. |
| **R1-3:** METHODS Were any patients censored before 12 months (potentially due to death or migration)? If so, how was their follow-up managed? | We thank the Reviewer for highlighting this issue. All patients were followed up for 12 months after the index date to determine if a cancer diagnosis was recorded in the cancer registry.  Patients who died (without cancer) during follow-up were not censored, but instead were kept in the sample and analysed as ‘not having cancer’.  The logic of this is that – in our view – the symptoms and blood test results examined in this study are not prognostic markers of patients at high risk of developing cancer, but diagnostic markers indicating the presence of a clinically-relevant but as-yet-undiagnosed tumour. Hence, we are interested in the cumulative incidence of cancer in the short-term after presentation, which given minimal censoring rates can be accurately estimated as the proportion of patients who have a recorded cancer in the year after index.  There are two limitations with this approach. First, some patients who die during follow-up without being diagnosed may have had an undiagnosed tumour, despite the registry capturing tumours that are recorded only on the death certificate. This may bias down the apparent cancer risk in groups with high competing mortality risk. Second, some patients may migrate out of England and so the registry would not capture their cancer diagnosis. This may reduce the apparent cancer risk in groups with higher rates of out-migration, which we assume will largely be younger patients who are typically at lower cancer risk in any case. We could potentially address this bias by adopting an approach that measured cumulative incidence, but to use such approaches we would need to know which patients migrated outside England and when – and this information was not available. |
| **R1-4:** Was any validation conducted of the codelist for abdominal pain and bloating? | A limitation of the CPRD dataset is that we do not have access to the free-text data so formal validation of the codelists against the complete GP record were not possible. However, established methods were used to create the codelists (13, 14) and each code list was cross checked by three clinicians, with any inconsistencies discussed and resolved to ensure accuracy. This has been summarized in the manuscript as follows:  Discussion (page 21 line3): *“comprehensive code lists for abdominal pain and bloating, developed using established methods (13, 14) and cross checked by three clinicians, were used to identify symptomatic patients in CPRD.”* |
| **R1-5:** Please include the codelist for the included blood tests. | This has now been added to the Supplementary File as requested and is mentioned in the manuscript as follows: Methods (page 6 line 28): “(see Supplementary Table S2 for codelists and evidence / rationale for inclusion of blood tests)” Example row of supplementary table S2: Supplementary Table S2: Evidence and rationale for blood tests included in the study *readterms assigned to each code were manually reviewed by a clinician and irrelevant terms were removed. **Blood test category** \| **Individual blood tests** \| **Rationale for inclusion** \| **Medcode** \| **CPRD test entity number***; Tumour markers \| Prostate specific antigen (PSA) and CA125 \| Established tumour markers used in primary care (15, 16). \| 5013 14054 17671 22598 108070 18455 108374 96961 98487 10363 7977 9228 14565 108230 \| 436, 276 Similar rows have been added for each of the 7 blood test categories. |
| **R1-6:** Please describe the extent to which you had access to the database populations used to create the study population. If this was conducted by others, please include them in the acknowledgements. | The CPRD team were provided with the symptom code lists, inclusion and exclusion criteria by the co-authors. CPRD used this to create a database of eligible patients aged ≥30 who had a recorded episode of abdominal pain or bloating recorded in CPRD. As is customary and according to our agreement, CPRD have been acknowledged in the data sharing statement as follows:  *“This study is based in part on data from the Clinical Practice Research Datalink obtained under licence from the UK Medicines and Healthcare products Regulatory Agency. The data is provided by patients and collected by the NHS as part of their care and support.”*  The study population was further refined by ourselves applying the described inclusion criteria. |
| **R1-7:** 5. Please confirm that the study included person-level data linkage. | We confirm the study included person-level data linkage. This has now been added to the manuscript as follows:  Methods (page 5 line 16): *“using primary care data from the UK Clinical Practice Research Datalink (CPRD) (17) linked at the person-level to English National Cancer Registry (18), Hospital Episode Statistics Admitted Patient Care (HES APC) (19) and Index of Multiple Deprivation (IMD) datasets.”* |
| **R1-8:** RESULTS The selection of included patients, including filtering based on data quality and linkage, should be described in detail. This would be best placed in the study flow diagram. | Please see response to comment **R1-6**. As is customary with the CPRD dataset, the inclusion and exclusion criteria specified by the co-authors was applied by data analysts from the CPRD team to produce the initial cohort of eligible patients aged ≥30 who had a new presentation of abdominal pain or bloating recorded in CPRD. We provide details of the selection process in the manuscript, but we are not provided with the number of patients excluded at each step. This study population was then further refined by ourselves to identify tested symptomatic patients, using the specified inclusion criteria. The selection of tested symptomatic patients, including our filtering/exclusion of patients, is presented in the study flow diagram (Figure 1). |
| **R1-9:** 2. Assuming censoring occurred, please summarise the average and total amount of follow-up time | Please see comment **R1-3** above. |
| **R1-10:** DISCUSSION  Please provide some discussion on the potential cost-effectiveness of changes to guidance. | Please see response to comment **AE-4** where this is now addressed. |
| **R1-11:** OTHER INFORMATION Please provide information on how to access the study protocol and programming code. | As per response to comment **E1-2,** we have now added the analysis plan as a Supplementary File and a sentence to the data sharing statement advising that the programming code can be provided on request.  Data sharing statement: *“Code is available from the Authors on request”* |
| **Reviewer 2 comments** | **Response** |
| **R2-1:** This research on abdominal pain/bloating, blood tests and PPV for cancers by age and sex has significant implications for general practice. | We thank the Reviewer for their positive assessment of our paper and its important clinical implications for general practice. |
| **R2-2:** In abstract, methods and findings (line 12): The authors state PPV, NPV, sensitivity and specificity were calculated; however, the focus was on PPVs. | PPVs were the focus of this study as this is the measure used by NICE to determine the criteria for recommending urgent cancer referral (using a PPV threshold of >3%), and therefore underpin the clinical translation and implications of the study findings. However, we do report NPV and additional diagnostic accuracy statistics (sensitivity, specificity and likelihood ratios) in the Tables and supplementary material to provide further context.  As the tests examined have low sensitivity for cancer, we concentrated on their potential rule in triage value (using PPVs), particularly where they increase a borderline pre-test risk (based on symptoms) to above the NICE 3% referral threshold. No single normal blood test result would suffice to rule out cancer. This is reflected in the negative likelihood ratios being >0.2. Negative likelihood ratios are considered to be one of the best measures of the rule out value of a test (20, 21).  This has now been specified more clearly in the manuscript as follows:  Methods (page 8, line 22): *“The predictive value of each individual blood test abnormality for cancer overall was then examined by age and sex using diagnostic accuracy statistics: We focused on PPVs as they are currently used by NICE to determine criteria for recommending urgent cancer assessment, using a PPV threshold of over 3% (22), and therefore have most clinical relevance for translation of the findings. Additionally, most of the relevant evidence also focuses on PPVs. Negative predictive value (NPV), sensitivity, specificity and positive and negative likelihood ratios were also estimated, alongside risk ratios comparing cancer risk in patients with a normal versus abnormal test result using Poisson regression.”*  Results (page 13 line 3): *“Normal results in no single blood test were sufficient to be used as a safe ‘rule-out’ test for cancer alone as reflected by the negative likelihood ratios >0.2 (20, 21, 23).”*  Discussion (page 24 line 25): *“No single blood test had sufficient rule-out value for cancer based on a normal result.”* |
| **R2-3:** Introduction (page 4, line 11): It would be good to understand what the NICE guidelines recommend. How urgent do the referrals need to be made? How urgent do the patients need to be seen by the referred? | Details of what the current NICE guidelines recommend have now been added to the manuscript to provide context for the international readership. This includes the expected timeframes following patient presentation with features of possible cancer and a referral / diagnosis.  Introduction (page 4 line 12): *“Faced with this challenge, policymakers such as the UK’s National Institute for Health and Care Excellence (NICE) have issued guidelines that recommend urgent cancer referral in patients with a cancer risk of ≥3% (to be made at the time of first clinical presentation with features of suspected cancer) and set the Faster Diagnosis Standard (FDS) specifying that a definitive diagnosis or ruling out of cancer should occur within 28 days of referral (24).”* |
| **R2-4:** Methods (page 5, line 12-15): Some context on the CPRD and English primary care health system would be useful to the international readership. | Details on CPRD and the context of the English primary care health system have now been added as follows:  Methods (page 5 line 18): *“In the UK healthcare system general practitioners (GPs) act as gatekeepers to secondary care and primary care is the setting where most symptomatic patients with underlying cancer first present (25). CPRD contains anonymised electronic primary care records from approximately 9% of all UK practices (17), including coded information on patient consultations, laboratory results and demographics.”* |
| **R2-5:** Results (page 8, line 28-29): "Cancer incidence was higher in males….." The authors are referring to PPV for cancer, not cancer incidence. It may also be worthwhile rearranging the sentence to replace "or" with another conjunction. (page 12, line 30 and elsewhere): The authors use "cancer risk >2%" to refer to "positive predictive value". These are different concepts. (page 13, line 12):"…the predictive value of blood test abnormalities was lower…". Could the authors be possibly referring to "…the PPV for cancers associated with blood test abnormalities…"? | We thank the Reviewer for raising the potential for confusion arising from the use of these, seemingly different, terms. We have now clarified their use / meaning in the text. We report the 1-year cancer incidence as a percentage for patients who present with abdominal pain or bloating. This is equivalent to both the PPV of the symptom for cancer and the one-year cancer risk(5). These terms are therefore used interchangeably throughout the manuscript. However, this has now been clearly specified in the manuscript to avoid confusion and assist interpretation as follows:  Methods (page 8 line 5): *“For each symptom, the 1-year cancer incidence was calculated from the first symptom date, stratifying by sex. This is equivalent to the positive predictive value (PPV) of abdominal pain or abdominal bloating for cancer, also referred to as the cancer risk* (5)*.”*  The sentence referred to has also been reworded to replace ‘or’ with ‘and’ as suggested:  Results (page 9 line 24): *“Cancer incidence was higher in males with either abdominal symptom than in females (PPV 2.8% and 2.6% in males compared to 1.9% and 2.1% in females, respectively for abdominal pain and bloating) (Table 2).”*  The suggested rewording has been implemented:  Results (page 14 line 17): *“In symptomatic patients aged 30 to 39 the PPVs for cancer associated with blood test abnormalities were lower”* |
| **R2-6:** Discussion (page 19, line 3-4 and page 19, line 25-26): Could the authors be possibly referring to the PPV for cancers again? | Please see response to comment **R2-5** above. |
| **R2-7:** (page 20, line 6-7): It would be helpful to know how GP decisions around blood testing could impact on PPVs. | Please see response to comment **AE-4** where this is now addressed. |
| **R2-8:** (page 22, line 18-19): The PPVs for cancers would be helpful for triaging;  however PPVs and cancer risks are different concepts. There may be a better term than "risk stratification". | Please see response to comment **R2-5** above. |
| **Reviewer 3 comments** | **Response** |
| **R3-1:** This is an interesting paper with clear background, mostly comprehensive description of methods and results, with plenty of relevant supplementary data. I enjoyed reading the manuscript.  Due to the amount of information, at times I struggled a bit to understand the key message from the paper, and what research is still needed if blood tests are to be used in primary care to triage patients. Would the team recommend all the investigated tests, for all aged 30-59, with a suspicion of (any) cancer? Further information on the clinical and practical implications of this would be useful. Therefore, I would recommend further revisions to the manuscript. | We thank the Reviewer for this encouraging assessment of our paper and the methods applied, and their constructive review.  We agree that further clarity of the key messages and clinical implications of the findings is needed and have now addressed this as per the response to comment **AE-4** above. |
| **R3-2:** Further clarifications on the model would be useful: Methods, page 8, line 14 - I presume that 100% guideline concordance was used as this is the gold standard, but is the gold standard realist? (see [https://eur01.safelinks.protection.outlook.com/?url=https%3A%2F%2Fbjgp.org%2Fcontent%2F66%2F643%2Fe106&data=05%7C02%7Cmeena.rafiq%40ucl.ac.uk%7C3868d611861e43eb7c1d08dc3c84fbe8%7C1faf88fea9984c5b93c9210a11d9a5c2%7C0%7C0%7C638451789057868387%7CUnknown%7CTWFpbGZsb3d8eyJWIjoiMC4wLjAwMDAiLCJQIjoiV2luMzIiLCJBTiI6Ik1haWwiLCJXVCI6Mn0%3D%7C0%7C%7C%7C&sdata=CX6LIsS2bsVp%2BomLteIbx0HOS0kONZJmprQAhdRYVpU%3D&reserved=0)/](https://bjgp.org/content/66/643/e106)  Would results change if estimated concordance was closer to real-life practice? This is briefly approached in the discussion, but the model was only done to show the "maximum potential impact" | Thank you for signposting this reference, which has now been added to the manuscript; further we have added citation to Wiering et al., which is another key study indicating that guideline concordance for suspected cancer referral is often way below 100%. Specifically, the two references are:   - Nicholson BD, Mant D, Neal RD, Hart N, Hamilton W, Shinkins B, et al. International variation in adherence to referral guidelines for suspected cancer: a secondary analysis of survey data. British Journal of General Practice. 2016;66(643):e106-e1 - Wiering B, Lyratzopoulos G, Hamilton W, Campbell J, Abel G. Concordance with urgent referral guidelines in patients presenting with any of six ‘alarm’ features of possible cancer: a retrospective cohort study using linked primary care records. BMJ Quality & Safety. 2022;31(8):579-89   As the Reviewer correctly points out, although we assumed 100% guideline concordance when modelling the maximum potential impact of implementing the findings on urgent referral numbers, real-life guideline adherence will likely be lower. This would likely result in fewer urgent referrals and potentially fewer additional cancers detected. This has now been expanded on in the limitations section of the discussion as follows:  Discussion (page 22 line 16): *“The estimates of potential changes in urgent referral numbers, and subsequent cancers detected, assume that proposed referral guidelines would be fully adhered to and therefore represent the maximum potential impact of including blood test results. This will however be lower in reality, as adherence to referral guidelines for suspected cancer is sub-optimal in clinical practice (26, 27), which would result in fewer urgent referrals and potentially fewer additional cancers detected than estimated*.” |
| **R3-3:** Results, page 18, lines 1-9: More information on the clinical relevance of the results would be useful. Using the tests would result in many more referrals, and a few extra cancers. At an individual level, one would want their cancer diagnosed despite resource implications. What about from a clinical perspective (both primary and secondary care)?  Because the model is also for any cancer and is a hypothetical model, there is no information on stage at diagnosis/tumour aggressiveness, what age groups would benefit, or whether these cancers would have been identified anyway, or later on (without impact on outcomes). Therefore, discussing possible clinical implications would be very helpful. | Please see response to comment **AE-4** where this is now addressed.  Stage at diagnosis:  We agree with the Reviewer’s consideration regarding stage at diagnosis, and this is a subject of recent ongoing interest in our community and our research group. For example, we have recently published a study examining the stage-specificity of risk of cancer after change in bowel habit and after rectal bleeding (Zakkak N et al., Cancer Epidemiology 2023(28)) and previously reported on the percentage of cancer patients who are diagnosed at stage 4 for 20 common presenting symptoms (Koo M et al., Lancet Oncology 2020(29)).  However, the great majority of currently available evidence and clinical practice recommendations regarding urgent referrals for suspected cancer (e.g. from NICE) do not take into account stage-specificity of the positive predictive value. There may be good arguments for not incorporating stage in such assessments of cancer risk, as both patients and clinicians prefer a more (as opposed to a less) prompt diagnosis, no matter the disease stage and associated prognosis. However, we do agree that stratification of risk estimates by disease stage will be important for modelling the public health impact and health economic analysis, as, exactly as the Reviewer discusses, some patients with the studied symptoms and positive test results will be diagnosed at advanced stage. We have added such a recommendation for future research in Discussion-Implications.  Age groups:  We thank the Reviewer for their comment which has helped us to further clarify the implications with regard to age groups. Patients aged 30-59 are expected to benefit the most from inclusion of blood tests in cancer guidelines as their prior (pre-blood test) referral risk based on their symptom-age-sex are below or near the 3% referral threshold. Patients aged ≥60 would meet referral thresholds based on their symptom presentation alone, but abnormal blood test results could increase clinician confidence in the need for further cancer assessment, thereby increasing guideline concordance.  These points have now been addressed in the discussion as follows:  Discussion (page 25 line 19): *“Consistent with most available evidence in our field and NICE guideline recommendations that do not incorporate consideration of stage at diagnosis, we have not stratified by stage at diagnosis. However, as cancers diagnosed through urgent referral routes have substantially higher 1-year survival (8) and diagnostic intervals are shorter for patients on urgent pathways (9) (10), we can posit that if the implementation of our study findings results in a greater number of cancers diagnosed through such referrals, this will also result in more cancers being diagnosed at an earlier stage improving prognosis. Formal modelling of cancer stage is however needed to confirm this.”*  Discussion (page 23 line 23): *“We found in patients of both sexes aged 60 years or over presenting with either of these symptoms, PPVs for cancer exceed the NICE risk threshold of 3%, and a potential cancer diagnosis and further urgent assessment should be considered in these patients regardless of the presence of other symptoms or blood test results. Detection of abnormal blood test results in this age group will increase clinician confidence in the decision for further cancer assessment, thereby increasing adherence to referral guidelines as it further strengthens the pre-test symptom-based assessment of cancer risk. This is important as adherence to cancer referral recommendations is currently sub-optimal, even when alarm (red-flag) symptoms are present (26, 27).”*  Discussion (page 24 line 5): *“Our findings show that existing results from readily available primary care blood tests could be better used to improve triaging and risk stratification of patients with abdominal pain and bloating in primary care, especially patients aged 30-59 where there is uncertainty regarding onward management because pre-blood test risk is below or near the 3% risk threshold above which urgent specialist assessment for suspected cancer is recommended.”*  Conclusion (page 26 line 14): *“The findings support the development of guideline recommendations, particularly in patients aged 30 to 59 with the studied symptoms who have a baseline risk of undiagnosed cancer below referral thresholds, where consideration of concurrent blood test abnormalities can enable specialist assessment where warranted”* |
| **R3-4:** Results, Box 1. Further clarifications on how these calculations were made would be quite helpful.  For example, for abdominal bloating we have 371 (symptom+age+sex model), 410 (as before + selected for test), and 473 (post-test risk, i.e as before + test results). How do these numbers result in 74 new referrals, 400 existing referrals and 10 referrals avoided?  Furthermore, I am not sure if the number of extra referrals in the final column is the difference between the pre-test and the post-test risk, or the difference between symptom+age+sex (i.e. first column) and post-test risk? Methods seem to indicate the latter, but I am unsure of it based on the results.  Finally, why is pre-test risk shown on top of the second column for abdominal bloating, but is the title for both column 1 and column 2 for abdominal pain? | We thank the Reviewer for highlighting this point. Details of how the calculations were made are as follows:     - Column 1: For each patient in the study population who presented with abdominal pain or bloating a referral recommendation (yes / no) was determined based on their symptom, age and sex by applying the age-sex specific PPVs for cancer (provided in column 2 of Table 2) using a referral threshold of >3%. - Column 2: The same process above was applied to the sub-cohort of patients in the study population who had an abdominal pain / bloating presentation + a blood test i.e. the ‘tested symptomatic cohort’. - Column 3: For each patient in the tested symptomatic cohort, a referral recommendation was made based on their symptom, age, sex and blood test results, by applying the age-sex specific PPVs for cancer for each blood test abnormality (provided in Table 3). - Column 4: For each individual patient in the tested symptomatic cohort any changes in referral recommendations from applying the process in columns 2 or 3 was summarized in column 4. In the example provided by the Reviewer, the 473 post-test referrals represents 400 existing referrals + ~74 new referrals and the 410 pre-test referrals represents 400 existing referrals + 10 referrals avoided. As the numbers are provided per 1,000 patients and provided as whole numbers, rounding means the numbers will not exactly equate to columns 2 and 3. - Column 5: extra referrals = post-test referrals (column 3) – pre-test referrals (column 2) e.g. 473 – 410 = 63 extra referrals.   These further clarifications have now been included in the legend to Box 1 as follows:  Box 1: Flow chart of 1,000 patients with abdominal bloating or pain modelling who would be recommended for an urgent cancer referral based on a pre-test vs post-test risk of cancer >3%.  Column 1 shows referral recommendations for our study population based on symptom (+ age and sex), applying the PPVs from Table 2; Column 2 shows recommendations based on symptom (+ age and sex) for patients in the study population who had a blood test around the time of abdominal pain/bloating presentation, applying the PPVs from Table 2; Column 3 shows updated referral recommendations for the tested study population based on symptom + blood test results (+ age and sex), applying the PPVs from Table 3; Column 4 shows the number of study patients who will experience a change in recommendation based on applying post-test risk (Yes / No) vs pre-test risk (Yes / No) (note as estimates are presented per 1,000 patients and rounded to whole number these figures will not exactly correspond to the numbers in columns 2 and 3); Column 5 shows the potential overall change in referral numbers and cancer diagnoses via urgent referral route using post-test risk (derived by comparing numbers from columns 2 and 3).  Regarding the location of the ‘pre-test risk’ title, this has now been moved so it is consistent across the abdominal pain and bloating panels. |
| **R3-5:** Discussion, page 20, line 18 - The authors raise the challenge of spectrum bias/effect here. It may be worth adding a caveat to the discussion: before using these blood tests for triage, or deciding the best way to use these tests in primary care, it is important that prospective studies are carried out in primary care so estimates are accurate for this population. What do authors think should be done next (i.e. recommendations for future research)? | Please see response to comment **AE-4** where this is now addressed.  The findings are based on prospectively collected primary care electronic health record data from ~500,000 patients in England. As the Reviewer points out, changes in blood test use in primary care over time (which could occur as an unintended consequence of including the study findings in NICE guidelines) would impact the PPVs in practice. Periodic re-estimation of PPVs will therefore be needed to account for this to ensure estimates remain accurate for the population. This has been included in the manuscript as follows:  Discussion (page 22 line 11): *“caution is needed when translating our findings to other settings or timeframes with different rates of primary care blood tests, and periodic re-estimation of the PPVs from this study will be needed to take into account possible changes in blood test use over time.”* |
| **R3-6:** Several tables in supplementary data: When each cancer site is looked at separately, we can then see that the value of tests (and different tests) vary a lot by cancer site. The PPVs are quite low for many cancers. The very high PPVs are for specific tumour types and specific tests e.g. ovarian cancer for raised CA125 or raised PSA and prostate cancer. What does that tell us in terms of the benefits of using all the studied blood tests, for all tumour types? Could the authors explore further the clinical implications, and the applicability of the results? | Please see response to comment **AE-4**, where we now clarify that we are not suggesting increased blood test use in patients presenting with these symptoms, but rather better use of existing test results.  This will include taking into account PPVs for different cancer sites by using the ranking of risk provided, regarding the most likely cancer sites associated with each blood test abnormality, which can be used to guide subsequent management (investigation/referral) decisions, including in rapid diagnostic centers where patients with non-specific symptoms are often referred. For example, it can inform the order of specific specialist tests to be considered (e.g. endoscopy and different imaging modalities of different sites) or the referral specialty (e.g. upper or lower gastroenterology or, in women, gynaecology). |
| ***Minor issues***  **R3-7:** Results, Table 1: There is a note about the blood tests being ordered for 15-pre-specified symptoms - could this be clarified? Is this this about the medical indication (in general) for such tests? | Thank you for highlighting this error in the legend. It has now been corrected as follows:  Table 1 footnote: *“*for ~~15 pre-specified symptoms and~~ 16 pre-specified blood tests in the 3 months before the index date.”* |
| **R3-8:** Results, page 9, lines 1-3. For those aged 60+ PPVs for either symptom already resulted in PPV above the NICE referral threshold. What would be the main benefit of such tests for this age group? Would easily accessible blood tests give confidence to the professional that this patient should be tested further? | As the Reviewer points out, our study provides estimates for the age and sex specific PPVs of abdominal pain and bloating for cancer. These estimates update previous evidence included in existing NICE guidelines and based on these findings we would now recommend all patients with these symptoms aged ≥60 be considered for urgent referral, regardless of blood test results.  Previous work by our co-authors has shown that a third of cancer patients who present with symptoms that meet the NICE 3% threshold are not urgently referred (Wiering et al., BMJ Qual & Safety 2022(27)). As the Reviewer highlights, abnormal blood test results in patients aged over 60 will have the added benefit of increasing clinician confidence in the need for further cancer assessment, thereby increasing guideline concordance as it further strengthens the pre-test assessment of cancer risk.  This important clinical implication has now been added to the manuscript as follows:  Discussion (page 23 line 23): *“We found in patients of both sexes aged 60 years or over presenting with either of these symptoms, PPVs for cancer exceed the NICE risk threshold of 3%, and a potential cancer diagnosis and further urgent assessment should be considered in these patients regardless of the presence of other symptoms or blood test results. Detection of abnormal blood test results in this age group will increase clinician confidence in the decision for further cancer assessment, thereby increasing adherence to referral guidelines as it further strengthens the pre-test symptom-based assessment of cancer risk. This is important as adherence to cancer referral recommendations is currently sub-optimal, even when alarm (red-flag) symptoms are present (26, 27).”*  As alluded to by the Reviewer, the age group which would benefit most from consideration of blood test results would be those aged 30-59. These patients have a ‘pre-test’ cancer risk below the referral threshold and limited guidelines currently exist in primary care on their management. Consideration of easily accessible blood test results in this group of patients would assist clinicians in identifying which of these patients should be tested further. This has now been clarified in the discussion as follows:  Discussion (page 24 line 5): *“Our findings show that existing results from readily available primary care blood tests could be better used to improve triaging and risk stratification of patients with abdominal pain and bloating in primary care, especially patients aged 30-59 where there is uncertainty regarding onward management because pre-blood test risk is below or near the 3% risk threshold above which urgent specialist assessment for suspected cancer is recommended”*  Conclusion (page 26 line 14): *“These findings support the development of guideline recommendations, particularly in patients aged 30 to 59 with the studied symptoms who have a baseline risk of undiagnosed cancer below referral thresholds, where consideration of concurrent blood test abnormalities can enable specialist assessment where warranted”* |
| **R3-9:** Could the authors justify why they chose to focus on abdominal pain and bloating among other possible non-specific symptoms? I understand abdominal pain is a non-specific symptom that can occur in many symptomatic presentations that may indicate different types of cancer (particularly GI cancers), but bloating is less common (although a more recognised risk for ovarian cancer). Since almost all cancer types were included, why these two? | We agree with the Reviewer that more profiling of additional symptoms would be useful and it is likely that the blood tests examined in this study could provide similar improvements in cancer risk assessment for patients with other non-specific symptoms. Indeed, our Group is about to engage in such follow-on studies. The decision to initially focus on abdominal pain and bloating was practical: We need to start from somewhere, and those two symptoms seem an excellent start, as they are known to have a relatively low (pre-test) cancer risk and are both associated with a range of different cancer sites (posing dilemmas as to diagnostic management) (11).  With regard to abdominal bloating, although it is conventionally considered to be a sign of ovarian cancer in women, both previous (11) and our own research indicates that a range of other cancers should also be considered in women, and indeed that risk is also appreciable (for different cancers) in men. We have now added this justification and area of potential future research to the manuscript as follows:  Introduction (page 4 line 4): *“Most non-specific abdominal symptoms, like pain or bloating, have a benign cause (30, 31), but in a small proportion of patients they are features of undiagnosed cancer of different sites (11, 32, 33).”*  Discussion (page 26 line 6): *“Finally, we focused on two non-specific abdominal symptoms in this study which pose a diagnostic challenge in primary care and are associated with risk of different cancer sites (11). It is likely consideration of blood test data will also enhance assessment of cancer risk for other non-specific symptoms (34, 35), which should be explored in follow-on research.”* |
| **R3-10:** The study included patients aged ≥30. Cancer risk increases with older age, so it makes sense not to include younger populations. However, could the chosen threshold be justified? | This justification of the age group has now been added to the manuscript as suggested:  Methods (page 6 line 8): *“Patients registered at a CPRD practice, aged ≥30 years, and with a new primary care record of either abdominal pain or bloating between 1^st^ January 2007 and 31^st^ October 2016 were identified. This age group was selected as cancer risk is considerably lower in patients aged <30 years, moreover cancer aetiology and cancer-site case mix different substantially, meriting a different, bespoke, study explicitly addressing cancer risk in symptomatic adolescents and younger adults.”* |
| **R3-11:** Results, Table 1 - It would have been interesting to have one line for male sex and another for female sex, particularly to see differences in testing for bloating | The number of blood test requested in patients with abdominal pain and bloating has now been presented separately for males and females as requested in Supplementary Table S7: Supplementary Table S7: Blood test use in the 3 months pre / post GP presentation with new onset abdominal pain or bloating in patients with ≥1 blood test, by sex SD, standard deviation; IQR, Interquartile range; *for 16 pre-specified blood tests in the 3 months before the index date; \| **Abdominal pain patients** \| \| **Abdominal bloating patients** \|; \| **Tested males** **N= 98,687** \| **Tested females** **N= 174,468** \| **Tested males** **N= 9,667** \| **Tested females** **N= 26,966**; Number of blood tests* \| \| \| \|; 1-4 \| 17,706 (18%) \| 38,956 (22%) \| 1,645 (17%) \| 5,204 (19%); 5-8 \| 26,078 (26%) \| 43,090 (25%) \| 2,539 (26%) \| 5,951 (22%); 9-12 \| 47,359 (48%) \| 79,141 (45%) \| 4,635 (48%) \| 13,241 (49%); ≥13 \| 7,544 (8%) \| 13,281 (8%) \| 848 (9%) \| 2,570 (10%); Mean (SD, range) \| 8.4 (4.4, 1-112) \| 8.3 (4.5, 1-114) \| 8.6 (4.4, 1-60) \| 8.6 (4.4, 1-70); Median (IQR) \| 9 (6-11) \| 9 (5-10) \| 9 (6-11) \| 9 (5-11) |
| **R3-12:** Table 3a is interesting and has a lot of information. It is good to see the cancer sites listed from highest to lowest risk. Is there any way to highlight amongst these the ones with a PPV of 3% and higher (maybe using the same colours as in the age cells (i.e. orange or red) for the font)? | We are pleased that the Reviewer found Table 3a informative. We have implemented their useful suggestion of colour coding cancer sites according to their risk with an example shown below. Table 3b: Positive predictive values (PPVs) of blood test abnormalities for cancer within 12 months in female patients with abdominal pain who had a blood test within 3 months of symptom presentation by sex and age group Red = PPV of ≥10%; Orange = PPV of ≥3% (NICE threshold for recommending cancer investigation); Yellow = PPV ≥2%. Cancer sites in bold have PPVs >1%; all listed cancer sites have PPVs >0.1%; CUP, cancer of unknown primary; other heam = other haematological cancer. **PPV in females with abdominal pain (95%CI) by age group (years)** \| \| \| \| \| \| \| **Cancer sites from highest to lowest risk**; **Blood abnormality** \| **30–39** \| **40–49** \| **50–59** \| **60–69** \| **70–79** \| **≥80** \|; **Baseline risk** \| **0.4 (0.4 – 0.5)** \| **0.8 (0.7 – 0.9)** \| **1.6 (1.4 – 1.7)** \| **3.4 (3.2 – 3.6)** \| **4.4 (4.2 – 4.7)** \| **5.2 (4.8 – 5.6)** \|; Raised CA125 \| 4.2 (1.4 – 9.5) \| 9.2 (6.4 – 12.6) \| 22.5 (16.6 – 29.3) \| 44.8 (35.9 – 54.0) \| 46.0 (37.1 – 55.1) \| 33.9 (22.3 – 47.0) \| **Ovary, bowel, pancreas, CUP,** uterus, other, sarcoma, lung, gastro-oesophageal, lymphoma, renal tract, liver, breast, melanoma, myeloma, cervix |
| **R3-13:** Conclusion section: Authors mention that the results from blood tests can substantially improve the clinical triage of patients, but no NPVs (very important is this scenario) were discussed (although they are available in Supplementary Table S9a and S9b). As before, spectrum bias is an issue.  Do we have sufficient evidence to write the suggested guidelines for those aged 30-59? | Please see response to comment **R2-2** where NPVs are addressed and comment **AE-4** where spectrum bias is addressed.  Regarding the question of whether the study provides sufficient evidence: The findings are based on prospectively collected routine electronic health record data from almost 500,000 patients in English primary care. The methodology and sample size are comparable to other study evidence used by NICE guidelines(5, 34). We are therefore confident that our findings provide sufficient evidence to inform guidelines in patients aged 30-59 presenting to UK primary care with these symptoms. |
| **R3-14:** Discussion, page 22, lines 1-4. This paragraph talks about using the results to help prioritise malignancies. Could this be clarified further? How would this be done? | In addition to providing PPVs for cancer overall we also estimated PPVs for different cancer sites and used this to provide rankings of the most likely cancer sites associated with each blood test abnormality. These ranking of level of risk pertaining to cancer of different organs (provided in Table 3) can be used by clinicians to determine which malignancies are most likely, to decide the order of subsequent investigation or referrals to different specialties. This has now been expanded on in the manuscript as follows:  Methods (page 8 line 28): *“Cancer-site specific PPVs were then calculated for each blood test (post hoc analysis) and used to rank cancer sites in order of likelihood to guide subsequent investigation / referral strategies.”*  Discussion (page 20, line 16): “Additionally, by ranking cancer-sites according to risk for each blood test abnormality, the findings can help guide investigation strategies post-referral.”  Discussion (page 21 line 17): *“This allowed identification of the cancer sites most strongly associated with each abnormal result. By providing rankings of the cancer sites most likely in patients with each blood test abnormality, the findings enable clinicians to prioritise investigations for malignancies in order of risk after referral”* |
| **R3-15:** Supplementary Table S10 and S11: I understand that the focus was on PPV due to the threshold for urgent referral, but NPVs may be more relevant here if the aim is to triage in primary care (as mentioned in the conclusion).  It would also be helpful to highlight the PPVs over 3%, as they show which blood tests are more relevant here, for which tumour type (e.g. anaemia, low albumin and low ferritin for bowel (male), PSA for prostate, and CA 125 for ovarian cancers. | Please also see response to comment **R2-2** above. Thank you for this suggestion. We have now highlighted cancer site specific PPVs >3% as shown in the example row below: Supplementary Table S11: Positive predictive values (PPVs) for different cancer sites within 12 months by sex in tested patients with abdominal pain or bloating and a blood test abnormality: males with abdominal pain, females with abdominal pain, males with abdominal bloating, females with abdominal bloating. PPVs >10% highlighted in red, >3% highlighted in orange, >2% highlighted in yellow, >1% highlighted in blue. **PPVs by cancer site in males with abdominal pain** \| \| \| \| \| \| \| \| \| \| \| \| \| \| \| \| \| \|; **cancer_site_final** \| **Anaemia** \| **Low albumin** \| **Low ferritin** \| **Low platelets** \| **Low WBC count** \| **Raised ALP** \| **Raised ALT** \| **Raised AST** \| **Raised bilirubin** \| **Raised calcium** \| **Raised creatinine** \| **Raised CRP** \| **Raised ESR** \| **Raised ferritin** \| **Raised HbA1c** \| **Raised platelets** \| **Raised PSA** \| **Raised WBC count**; **Bowel** \| 3.41 \| 3.05 \| 5.93 \| 0.30 \| 0.44 \| 1.72 \| 0.35 \| 0.55 \| 0.51 \| 1.88 \| 1.22 \| 1.89 \| 2.20 \| 0.39 \| 1.06 \| 4.48 \| 1.50 \| 1.78 |
| **R3-16:** Results, Table 1 - Numbers for 2015-2016 seem to be much smaller compared to other years (even when considering that data are only available up to October 2016). Was there also a time lag in the data (due to delays in CRPD receiving primary care information) when extraction happened? | Thank you for highlighting this. The progressive decrease of our analysis sample over time is due to two factors: Firstly, there is a progressive decrease in patients included in CPRD Gold, as it was progressively replaced by CPRD Aurum during our study period, with practices moving out of the Vision IT system which supported CPRD Gold (dropping from 674 practices in July 2013(17) to 403 in February 2022(36)); Additionally, as we selected the first eligible symptom presentation in a patient’s record it is expected that the sample will be skewed towards the start of the study period. We have explained this in the manuscript and as a footnote of Table 1 as follows:  Methods (page 6 line 15): *“The first eligible episode of each symptom was selected (index date) and used to create two cohorts (abdominal pain and abdominal bloating).”*  Table 1 footnote: *“^Ψ^ The progressive decrease of our analysis sample over time reflects the progressive decrease of patients included in CPRD Gold (as it was progressively replaced by CPRD Aurum during our study period, with practices moving out of the Vision IT system which supported CPRD Gold), and the selection of the first eligible symptom presentation in the study period.”* |
| **R3-17:** Figure 2 is a bit blurred, but I checked the original submission and it is much more visible so it may have just been an issue with resubmission. I would suggest replacing "abdominal symptoms" in the title with "abdominal pain/bloating". | This Figure has now been reloaded and the suggested change in the title has been made as follows:  *“Figure 2: Pre-test and Post-test Positive predictive values (PPVs) for cancer in 12 months by sex in patients with abdominal pain / bloating using results of commonly used blood tests.”* |
| **R3-18:** Supplementary Figure S2: Consider making it clear that the "cohorts" here refer to the "Scenarios" described in the paper | This useful suggestion has now been implemented as follows:  Supplementary Figure S1: Creation of the final tested symptomatic population by combining two subgroups of patients who had a blood test request around the time of abdominal symptom presentation identified by two different presentation scenarios.   |
| **Reviewer 4 comments** | **Response** |
| **R4-1:** This is an interesting study on the predictive value of abnormal blood tests for detecting cancer in primary care patients with non-specific abdominal symptoms using the UK CPRD data linked to the National Cancer Registry. However, there are a few major issues needing attention. | We thank the Reviewer for their concise and accurate summary of our study, and their constructive comments. |
| **R4-2:** The result and interpretation of PPV and NPV are difficult to follow. In a typical prediction study, we would expect PPV and NPV to be 80%-90% so that the rules can be applied in practice. However, here in the paper, we are talking about 2-3% then improved to 8%-10% when add the information of the blood tests.  It's obvious that the PPV will improve when add more information such as these blood tests. We know this.  More importantly, it would be good to use these blood tests to bulid a comprehensive multivariate prediction model to predict either referral or incidence of cancer. So far the paper is mostly exploratory on extra information from blood test and hasn't given a solid and overall solution. | Because the symptoms of cancer are frequently encountered in other diseases and cancer is not particularly common, PPVs of between 0-10% are typically reported for symptoms of possible cancer (37). A threshold of 3% has been chosen by NICE to define the threshold for clinically significant risk, above which an urgent referral for specialist assessment of suspected cancer is recommended. Given this background, an increase from 2% to 8-10% is critically important as it makes a difference as to whether a patient will be further investigated and referred to secondary care or not, according to current guidelines.  Adding information from blood tests to PPV estimation could either increase or decrease the value of the PPV depending on the predictive value of the additional data. The magnitude of any increase in PPV is also clinically important, particularly if it reclassifies a patient with a pre-test risk below the referral threshold to a post-test risk where urgent referral would be indicated. We demonstrate this to be the case for several blood test abnormalities in this study, even considering results from one blood test at a time.  We agree that extending this work by combining results from multiple pre-diagnostic features could further increase the estimated PPVs and have highlighted this as an area of further research, although the extent to which multiple abnormal tests will improve the PPV beyond the value of a single abnormal test is not known and needs to first be established empirically. However, the PPVs of symptom-single test combinations demonstrated in this study provide evidence to reclassify some patients aged 30-59 who present with abdominal pain and bloating and therefore to inform current updates to guidelines which have potential for further improvement in the future.  We would not agree with the assertion that the findings are not useful unless included in a ‘multivariate prediction model’. The current NICE guidelines operate on the basis of simple clinical scenarios characterised by the presence / absence of presenting features (typically symptoms, and following our findings, this can be extended to symptom-blood test result combinations). This is the most practical way by which our evidence can be implemented, i.e. through inclusion in clinical guideline recommendations. We do agree that added usefulness can be gained through prediction modelling, but it is quite well-established in our community that the clinical implementation of such models is very problematic and they are rarely used in practice. There should be no doubt about the importance of our findings with regard to clinical practice translation and relevance to guideline recommendations. |
| **R4-3:** The statistical analyses are a bit simplistic and mainly univariate. The combined impact of 19 blood tests would be preferred using the multivariate analysis. | Please see response to comment **R4-2** above where this is addressed. |
| **R4-4:** Overall, the paper pointed out these blood tests were useful, which is mostly known. However, it doesn't go further to build a useful and practical model to be used in the clinical settings. | Please see response to comment **R4-2** above where this is addressed.  We do not agree with the Reviewer that descriptive risk studies such as ours are not as useful as risk prediction studies. This question can be the subject of a debate article, but our community is by no means united in the confidence shown by the Reviewer regarding the usefulness and clinical impact of risk prediction models, which are rarely used in practice, even when the C statistic or other measures of discrimination are high. |

1.	Watson J, Hamilton F, Bailey S, Mounce L, Hamilton W. Clinical implications of increased testing in primary care. 2019.

2.	Watson J. Lessons from biases in electronic health record data: the importance of clinical vigilance with negative test results 2018 [Available from: <https://www.bmj.com/content/361/bmj.k1479/rr-0>

.

3.	Watson J, Salisbury C, Banks J, Whiting P, Hamilton W. Predictive value of inflammatory markers for cancer diagnosis in primary care: a prospective cohort study using electronic health records. British journal of cancer. 2019;120(11):1045.

4.	Ankus E, Price SJ, Ukoumunne OC, Hamilton W, Bailey SE. Cancer incidence in patients with a high normal platelet count: a cohort study using primary care data. Family practice. 2018;35(6):671-5.

5.	Bailey SE, Ukoumunne OC, Shephard EA, Hamilton W. Clinical relevance of thrombocytosis in primary care: a prospective cohort study of cancer incidence using English electronic medical records and cancer registry data. Br J Gen Pract. 2017;67(659):e405-e13.

6.	Mounce LT, Hamilton W, Bailey SE. Cancer incidence following a high-normal platelet count: cohort study using electronic healthcare records from English primary care. British Journal of General Practice. 2020;70(698):e622-e8.

7.	Cranfield BM, Abel GA, Swann R, Moore SF, McPhail S, Rubin GP, Lyratzopoulos G. Pre-Referral Primary Care Blood Tests and Symptom Presentation before Cancer Diagnosis: National Cancer Diagnosis Audit Data. Cancers. 2023;15(14):3587.

8.	Elliss-Brookes L, McPhail S, Ives A, Greenslade M, Shelton J, Hiom S, Richards M. Routes to diagnosis for cancer–determining the patient journey using multiple routine data sets. British journal of cancer. 2012;107(8):1220.

9.	Neal RD, Din NU, Hamilton W, Ukoumunne O, Carter B, Stapley S, Rubin G. Comparison of cancer diagnostic intervals before and after implementation of NICE guidelines: analysis of data from the UK General Practice Research Database. British journal of cancer. 2014;110(3):584-92.

10.	Neal RD, Allgar VL, Ali N, Leese B, Heywood P, Proctor G, Evans J. Stage, survival and delays in lung, colorectal, prostate and ovarian cancer: comparison between diagnostic routes. British Journal of General Practice. 2007;57(536):212-9.

11.	Herbert A, Rafiq M, Pham TM, Renzi C, Abel GA, Price S, et al. Predictive values for different cancers and inflammatory bowel disease of 6 common abdominal symptoms among more than 1.9 million primary care patients in the UK: A cohort study. PLoS medicine. 2021;18(8):e1003708.

12.	Chima S, Reece JC, Milley K, Milton S, McIntosh JG, Emery JD. Decision support tools to improve cancer diagnostic decision making in primary care: a systematic review. British Journal of General Practice. 2019;69(689):e809-e18.

13.	Watson J, Nicholson BD, Hamilton W, Price S. Identifying clinical features in primary care electronic health record studies: methods for codelist development. BMJ open. 2017;7(11):e019637.

14.	Davé S, Petersen I. Creating medical and drug code lists to identify cases in primary care databases. Pharmacoepidemiology and drug safety. 2009;18(8):704-7.

15.	Young GJ, Harrison S, Turner EL, Walsh EI, Oliver SE, Ben-Shlomo Y, et al. Prostate-specific antigen (PSA) testing of men in UK general practice: a 10-year longitudinal cohort study. BMJ open. 2017;7(10):e017729.

16.	Crawford SM, Evans C. Outcome of elevated CA125 values from primary care following implementation of ovarian cancer guidelines. Family practice. 2017;35(2):199-202.

17.	Herrett E, Gallagher AM, Bhaskaran K, Forbes H, Mathur R, van Staa T, Smeeth L. Data Resource Profile: Clinical Practice Research Datalink (CPRD). Int J Epidemiol. 2015;44(3):827-36.

18.	Henson KE, Elliss-Brookes L, Coupland VH, Payne E, Vernon S, Rous B, Rashbass J. Data resource profile: National cancer registration dataset in England. International journal of epidemiology. 2020;49(1):16-h.

19.	Herbert A, Wijlaars L, Zylbersztejn A, Cromwell D, Hardelid P. Data resource profile: hospital episode statistics admitted patient care (HES APC). International journal of epidemiology. 2017;46(4):1093-i.

20.	Jaeschke R, Guyatt GH, Sackett DL, Guyatt G, Bass E, Brill-Edwards P, et al. Users' guides to the medical literature: III. How to use an article about a diagnostic test B. What are the results and will they help me in caring for my patients? Jama. 1994;271(9):703-7.

21.	Thompson M, Van den Bruel A, Verbakel J, Lakhanpaul M, Haj-Hassan T, Stevens R, et al. Systematic review and validation of prediction rules for identifying children with serious infections in emergency departments and urgent-access primary care. Health Technol Assess. 2012;16(15):1-100.

22.	Moore SF, Price SJ, Chowienczyk S, Bostock J, Hamilton W. The impact of changing risk thresholds on the number of people in England eligible for urgent investigation for possible cancer: an observational cross-sectional study. British Journal of Cancer. 2021;125(11):1593-7.

23.	Koshiaris C, Van den Bruel A, Oke JL, Nicholson BD, Shephard E, Braddick M, Hamilton W. Early detection of multiple myeloma in primary care using blood tests: a case–control study in primary care. Br J Gen Pract. 2018;68(674):e586-e93.

24.	NHS England. Faster Diagnosis Framework and the Faster Diagnostic Standard 2021 [Available from: <https://www.england.nhs.uk/cancer/faster-diagnosis/#fds>.

25.	Hamilton W. Cancer diagnosis in primary care. British Journal of General Practice. 2010;60(571):121-8.

26.	Nicholson BD, Mant D, Neal RD, Hart N, Hamilton W, Shinkins B, et al. International variation in adherence to referral guidelines for suspected cancer: a secondary analysis of survey data. British Journal of General Practice. 2016;66(643):e106-e13.

27.	Wiering B, Lyratzopoulos G, Hamilton W, Campbell J, Abel G. Concordance with urgent referral guidelines in patients presenting with any of six ‘alarm’features of possible cancer: a retrospective cohort study using linked primary care records. BMJ Quality & Safety. 2022;31(8):579-89.

28.	Zakkak N, Lyratzopoulos G, Barclay M. Stage-specific risk of colon and rectal cancer in patients presenting with rectal bleeding or change in bowel habit in primary care: A population-based cohort study. Cancer Epidemiology. 2023;87:102484.

29.	Koo MM, Swann R, McPhail S, Abel GA, Elliss-Brookes L, Rubin GP, Lyratzopoulos G. Presenting symptoms of cancer and stage at diagnosis: evidence from a cross-sectional, population-based study. The Lancet Oncology. 2020;21(1):73-9.

30.	Agreus L, Svärdsudd K, Nyren O, Tibblin G. The epidemiology of abdominal symptoms: prevalence and demographic characteristics in a Swedish adult population: a report from the Abdominal Symptom Study. Scandinavian journal of gastroenterology. 1994;29(2):102-9.

31.	Kay L, Jørgensen T, Jensen KH. Epidemiology of abdominal symptoms in a random population: prevalence, incidence, and natural history. European journal of epidemiology. 1994;10:559-66.

32.	Koo MM, Hamilton W, Walter FM, Rubin GP, Lyratzopoulos G. Symptom signatures and diagnostic timeliness in cancer patients: a review of current evidence. Neoplasia. 2018;20(2):165-74.

33.	Price SJ, Gibson N, Hamilton WT, King A, Shephard EA. Intra-abdominal cancer risk with abdominal pain: a prospective cohort primary care study. British Journal of General Practice. 2022;72(718):e361-e8.

34.	Nicholson BD, Aveyard P, Price SJ, Hobbs FR, Koshiaris C, Hamilton W. Prioritising primary care patients with unexpected weight loss for cancer investigation: diagnostic accuracy study. bmj. 2020;370.

35.	White B, Rafiq M, Gonzalez-Izquierdo A, Hamilton W, Price S, Lyratzopoulos G. Risk of cancer following primary care presentation with fatigue: a population-based cohort study of a quarter of a million patients. British Journal of Cancer. 2022;126(11):1627-36.

36.	Clinical Practice Research Datalink. CPRD GOLD February 2022 dataset 2022 [Available from: <https://doi.org/10.48329/kxsp-t315>.

37.	Hamilton W. The CAPER studies: five case-control studies aimed at identifying and quantifying the risk of cancer in symptomatic primary care patients. British journal of cancer. 2009;101(S2):S80.
