# Supplementary material for: Predictive value of abnormal blood tests for detecting cancer in primary care patients with nonspecific abdominal symptoms: A population-based cohort study of 477,870 patients in England
Source: PLoS Med. 2024 Jul 30;21(7):e1004426. doi: 10.1371/journal.pmed.1004426 (PMC11288431; doi:10.1371/journal.pmed.1004426)
Supplement: Attachment — Submitted filename: Response to reviewers.docx [file pmed.1004426.s004.docx]

**Authors’ reply to Reviewer Comments, Manuscript PMEDICINE-D-24-00134R1, by Rafiq et al., “Predictive value of abnormal blood tests for detecting cancer in primary care patients with non-specific abdominal symptoms: A population-based cohort study of 477,870 patients in England”.**

Dear Dr Dodd,

Thank you for accepting the above paper for publication in PLOS Medicine.

As requested I have now addressed the remaining minor issues listed in your email. For ease of reading any altered text from the manuscript has been included in the point-to-point response to Reviewers’ with the new text highlighted in yellow alongside a CLEAN and TRACKED version of the manuscript.

| **Editorial comments** | **Response** |
| --- | --- |
| GENERAL Thank you for your detailed and considered responses to previous comments. We agree with reviewer #4 (please see below) that including additional rationale within the manuscript (as in your rebuttal) regarding the chosen analytical approach would be helpful to the reader. | We thank the Editor for their positive assessment of our reviewer responses. As recommended we have now added the rationale for our chosen analytical approach and a discussion about the potential role of multivariable risk prediction models as follows:  Discussion, page 27 line 3: *“The predictive accuracy of individual blood test abnormalities examined in this study may also be further enhanced by combining effects from different blood test results, their trends over time and other clinical features using multivariable prediction models (1-3). The extent that multiple abnormal tests will improve the PPV beyond the value of a single abnormal test is not known and needs to first be established empirically, accompanied by evidence on how to implement these models and ensure their uptake in clinical practice(4). The current NICE guidelines operate on the basis of simpler clinical scenarios characterised by the presence / absence of presenting features. Our study findings of various symptom-single test combinations that can reclassify some patients aged 30-59 who present with abdominal pain and bloating as having a risk of underlying cancer >3% could inform updates to these clinical guidelines, with potential for further improvement in the future as evidence emerges.”* |
| AUTHOR SUMMARY At this stage, we ask that you include a short, non-technical Author Summary of your research to make findings accessible to a wide audience that includes both scientists and non-scientists. The authors summary should consist of 2-3 succinct bullet points under each of the following headings: • Why Was This Study Done? Authors should reflect on what was known about the topic before the research was published and why the research was needed. • What Did the Researchers Do and Find? Authors should briefly describe the study design that was used and the study’s major findings. Do include the headline numbers from the study, such as the sample size and key findings. • What Do These Findings Mean? Authors should reflect on the new knowledge generated by the research and the implications for practice, research, policy, or public health. Authors should also consider how the interpretation of the study’s findings may be affected by the study limitations. In the final bullet point of ‘What Do These Findings Mean?’, please describe the main limitations of the study in non-technical language. | This has now been added to the manuscript as follows:  **Why Was This Study Done?**   - Half of all patients with as-yet-undetected cancer will first present with non-specific symptoms that can be challenging to diagnose. - Many of these patients are investigated in primary care with commonly used blood tests that could help to identify which patients are most likely to have underlying cancer (to prioritise them for referral) and which patients can be safely monitored in primary care. - This study aimed to assess the predictive value of abnormal blood tests for detecting cancer in primary care patients presenting with two non-specific abdominal symptoms.   **What Did the Researchers Do and Find?**   - Using linked UK primary care data (CPRD) we conducted a cohort study of 477,870 patients aged ≥30 years presenting with new abdominal pain or bloating and calculated the predictive value of 19 abnormal blood test results for detecting cancer by age and sex. - Males and females aged ≥60 presenting with either symptom had a risk of underlying cancer exceeding the 3% threshold used by the UK National Institute for Health and Care Excellence for recommending urgent cancer referral. - In patients aged 30-59 with abdominal pain or bloating, several blood abnormalities updated a patient’s cancer risk to above the 3% threshold and they should be considered for urgent cancer referral.   **What Do These Findings Mean?**   - Commonly used primary care blood test results can improve the detection of underlying cancer in patients consulting with non-specific abdominal symptoms. - These findings can inform updates to clinical guidelines to allow detection of additional patients with underlying cancer through expedited referral routes and can guide decisions on specialist referrals and investigation strategies for different cancer sites. - Limitations include the results applying to patients who had been recorded as having abdominal pain and bloating by their clinician and who had been selected by the clinician for blood testing (and therefore have a higher cancer risk that all patients with abdominal pain and bloating). |
| STUDY REPORTING Thank you for including the STROBE checklist. Please amend to refer to section and paragraph numbers, rather than page numbers as the latter often change at publication. | The STROBE checklist has now been updated as requested with page numbers replaced with section names and paragraph numbers. |
| DATA AVAILABILITY  Authors are responsible for providing the source code needed to replicate the study's findings in a repository (such as GitHub, SourceForge or Bitbucket). Please explain in the manuscript’s Data Availability Statement how readers can access the shared code. Please note that an author cannot serve as the point of contact for requests. | The code has now been added to Open Science Framework (OSF) and is publicly available. The data availability statement has been updated to reflect this as follows:  Data sharing statement  This study is based in part on data from the Clinical Practice Research Datalink obtained under licence from the UK Medicines and Healthcare products Regulatory Agency. The data is provided by patients and collected by the NHS as part of their care and support. The interpretation and conclusions contained in this study are those of the author/s alone. Code is available from https://osf.io/cqzwd/ . Due to privacy laws and the data user agreement between UCL and CPRD, researchers are not authorised to share individual patient data.  Access to CPRD data, including UK Primary Care Data, and linked data such as Hospital Episode Statistics, is subject to protocol approval via CPRD’s Research Data Governance (RDG) Process, see https://cprd.com/data-access for further details. |
| STATISTICAL REPORTING Throughout all subsections, including tables and figures, please quantify the main results with 95% CIs and p values. When reporting p values please report as <0.001 and where higher as p=0.002, for example. If not reporting p values, for the purpose of transparent data reporting, please clearly state the reasons why not. When reporting 95% CIs please separate upper and lower bounds with commas instead of hyphens as the latter can be confused with reporting of negative values. Please include the actual amounts and/or absolute risk(s) of relevant outcomes (including NNT or NNH where appropriate), not just relative risks or correlation coefficients. (example for absolute risks: PMID: 28399126). | The content and style of the results has been checked to make it is in line with the PLOS medicine reporting style. |
| ABSTRACT Abstract Methods and Findings: Please ensure that all numbers presented in the abstract are present and identical to numbers presented in the main manuscript text. Please include the study design, population and setting, number of participants, years during which the study took place, length of follow up, and main outcome measures. Please quantify the main results (with 95% CIs and p values). Please include the important dependent variables that are adjusted for in the analyses. Please include the actual amounts and/or absolute risk(s) of relevant outcomes (including NNT or NNH where appropriate), not just relative risks or correlation coefficients. (example for absolute risks: PMID: 28399126). Please include a summary of adverse events if these were assessed in the study. In the last sentence of the Abstract Methods and Findings section, please describe the main limitation(s) of the study's methodology. | The content and style of the abstract has been checked to make it is in line with the PLOS medicine reporting style. |
| INTRODUCTION Please ensure that you address past research and explain the need for and potential importance of your study. Indicate whether your study is novel and how you determined that. If there has been a systematic review of the evidence related to your study (or you have conducted one), please refer to and reference that review and indicate whether it supports the need for your study. | The content and style of the introduction has been checked to make it is in line with the PLOS medicine reporting style. |
| METHODS and RESULTS In the manuscript text, please ensure that you have indicated: (1) the specific hypotheses you intended to test, (2) the analytical methods by which you planned to test them, (3) the analyses you actually performed, and (4) when reported analyses differ from those that were planned, transparent explanations for differences that affect the reliability of the study's results. If a reported analysis was performed based on an interesting but unanticipated pattern in the data, please be clear that the analysis was data-driven. Please remove the word ‘retrospective’ and refer to the study as simply a population-based cohort study (page 5, line 15, for example). Please check and amend throughout all sub-sections of the manuscript and supporting files. Please ensure that you report the number of [patients, samples, etc] and dates of recruitment, and account for all methods used in your study. Please provide the actual numbers of events for the outcomes, not just summary statistics or ORs. Please ensure to present numerators and denominators used to derive percentages. Please ensure to follow the statistical reporting guidance as detailed above, reporting 95% CIs alongside p values. When reporting p values please also provide the statistical test used to determine them. | The content and style of the methods and results has been checked to make it is in line with the PLOS medicine reporting style. As requested, the word ‘retrospective’ has been removed from cohort study throughout. |
| TABLES and FIGURES Please ensure all tables/figures are affiliated to an appropriate title/caption/footnote which clearly describes their content without the need to refer to the text. The current table titles are long and at times it is difficult to fully understand what the tables are showing. Please ensure all abbreviations are clearly defined within the footnote, including those used for statistical reporting. Please separate upper and lower bounds of 95% CIs with commas as opposed to hyphens (to prevent confusion with reporting of negative values). Where 95% CIs are reported, please also report p values as detailed above (see under statistical reporting). Please ensure that you indicate whether analyses are adjusted or unadjusted and, where adjusted analyses are presented, please also present the unadjusted analyses for comparison. Where relevant please indicate which factors are adjusted for. Please ensure that you indicate for the reader the meaning the meaning of all dots, lines and bars used in the figures. Figure 2, for example, would be benefit from some additional information. To make your figures more accessible to those with colour blindness please consider avoiding the use of green and/or red. | The content and style of the methods and results has been checked to make it is in line with the PLOS medicine reporting style. As requested, the table titles have been made more concise and the legend for Figure 2 has been made more informative. The colours in Figure 2 have now been altered to be accessible to colour blind readers and checked using the following resources to ensure they are colour-blind friendly:   - [**https://colorbrewer2.org/#type=qualitative&scheme=Dark2&n=3**](https://colorbrewer2.org/#type=qualitative&scheme=Dark2&n=3) - [**https://davidmathlogic.com/colorblind/#%231B9E77-%23D95F02-%237570B3-%2388CCEE**](https://davidmathlogic.com/colorblind/#%231B9E77-%23D95F02-%237570B3-%2388CCEE) |
| DISCUSSION Please remove all sub-headings from the discussion such that it reads as continuous prose. Page 25, line 1 – suggest instead, ‘We report the relative risk of cancer at different sites…’ | The content and style of the discussion has been checked to make it is in line with the PLOS medicine reporting style and the highlighted line edited as suggested. |
| REFERENCES For in-text reference callouts please place citations in square parentheses separate by commas. For example, [1,3,6] or [1-3]. Please check and amend throughout all sub-sections of the manuscript and supporting files. In the bibliography, please ensure that you list up to but no more than 6 author names followed by et al. For all web references please ensure you include an, ‘Accessed [date].’ Journal name abbreviations should be those listed in the National Center for Biotechnology Information (NCBI) databases. | The content and style of the references is in line with the PLOS medicine style. The access dates for the websites have now been added. |
| SOCIAL MEDIA To help us extend the reach of your research, please detail any X (formerly Twitter) handles you wish to be included when we tweet this paper (including your own, your coauthors’, your institution, funder, or lab) in the manuscript submission form when you re-submit the manuscript. | @DrMeenaRafiq @UCL_BSH |
| **Reviewer 3 comments** |  |
| I believe that the authors have comprehensively addressed all my comments. I have read through all responses, and checked the manuscript again. It reads well, and its message is much clearer now. I have no further queries about it. | We thank the Reviewer for their comments and positive assessment of our paper. |
| Please see below some very minor issues I noticed when re-reading the paper just in case the authors wish to address these. They are mostly cosmetic:  1. Methods, Page 5, Line 30: You may wish to add a single quotation mark to the word 'tested' here as this is how it seems to be shown elsewhere (it may be worth doing a quick search to confirm the term is shown consistently throughout the manuscript). | Thank you for pointing out these minor amendments. We have now addressed each of them as follows:  Secondly, a sub-group of the above population, comprising symptomatic patients (as above) who also had blood tests around the time of their presentation with the studies symptoms (*the ‘tested’ symptomatic population*) |
| 2. Table 1: Notes added here end with double quotation marks (perhaps when the text was copied from the response to reviewers?). Should these quotation marks be removed? | This typo has now been removed. |
| 3. Table 3a: Should the abbreviation be "haem" instead of "heam"? If so, the same issue needs to be corrected in the last column of table 3b (row for low albumin) | The Reviewer is correct and this typo has now been corrected. |
| 4. Supplementary Table S10a - A zero may be missing after 67.7 (Sensitivity for Raised ESR) as all other values have two decimal points | Thank you for your thorough review and pointing out this typo which has now been corrected. |
| **Reviewer 4 comments** |  |
| I am mostly satisfied with the responses. However, some of the responses are useful to readers and would be good to appear in the discussion section of the paper, such as the debate on multivariable prediction model vs simple/single tests and how they are related to and recommended in guidelines (R4-2 and R4-4). | We are glad that our responses have addressed the comments made by the Reviewer. As suggested, we have now included some of the text from responses R4-2 and R4-4 to the discussion section of the manuscript  Discussion, page 27 line 3: *“The predictive accuracy of individual blood test abnormalities examined in this study may also be further enhanced by combining effects from different blood test results, their trends over time and other clinical features using multivariable prediction models (1-3). The extent that multiple abnormal tests will improve the PPV beyond the value of a single abnormal test is not known and needs to first be established empirically, accompanied by evidence on how to implement these models and ensure their uptake in clinical practice(4). The current NICE guidelines operate on the basis of simpler clinical scenarios characterised by the presence / absence of presenting features. Our study findings of various symptom-single test combinations that can reclassify some patients aged 30-59 who present with abdominal pain and bloating as having a risk of underlying cancer >3% could inform updates to these clinical guidelines, with potential for further improvement in the future as evidence emerges.”* |

1.	Nicholson BD, Aveyard P, Koshiaris C, Perera R, Hamilton W, Oke J, Hobbs FR. Combining simple blood tests to identify primary care patients with unexpected weight loss for cancer investigation: Clinical risk score development, internal validation, and net benefit analysis. PLoS medicine. 2021;18(8):e1003728.

2.	Nicholson BD, Aveyard P, Price SJ, Hobbs FR, Koshiaris C, Hamilton W. Prioritising primary care patients with unexpected weight loss for cancer investigation: diagnostic accuracy study. bmj. 2020;370.

3.	Watson J, Salisbury C, Banks J, Whiting P, Hamilton W. Predictive value of inflammatory markers for cancer diagnosis in primary care: a prospective cohort study using electronic health records. British journal of cancer. 2019;120(11):1045.

4.	Usher-Smith J, Emery J, Hamilton W, Griffin SJ, Walter FM. Risk prediction tools for cancer in primary care. British journal of cancer. 2015;113(12):1645-50.
